# Uncovering the potent antimicrobial activity of squaramide based anionophores – chloride transport and membrane disruption†

**DOI:** 10.1039/d4sc01693a

**Published:** 2025-01-22

**Authors:** Luke E. Brennan, Xuanyang Luo, Farhad Ali Mohammed, Kevin Kavanagh, Robert B. P. Elmes

**Affiliations:** a Department of Chemistry, Maynooth University Maynooth Co. Kildare Ireland; b Synthesis and Solid-State Pharmaceutical Centre (SSPC) Ireland; c Department of Biology, Maynooth University Maynooth Co. Kildare Ireland; d Kathleen Lonsdale Institute for Human Health Research, Maynooth University Maynooth Co. Kildare Ireland Robert.elmes@mu.ie

## Abstract

Antimicrobial resistance (AMR) – often referred to as a silent pandemic, is at present the most serious threat to medicine, and with constantly emerging resistance to novel drugs, combined with the paucity of their development, is likely to worsen. To circumvent this, supramolecular chemists have proposed the applicability of synthetic anion transporters in the fight against AMR. In this article we discuss the synthesis, supramolecular characterisation and biological profiling of six structurally simple squaramide anion transporters. Through a combination of spectroscopic techniques, and cellular assays we have deduced the mode of action of these antimicrobial agents to be as a result of both anion transport and membrane disruption. Furthermore, through the synthesis of two fluorescent analogues we verified this membrane-localised activity using Super-Resolution nanoscopy methods. These compounds represent particularly active antimicrobial anionophores and compliment similar reports showing the applicability of agents such as these in the fight against AMR.

## Abstract 2

Squaramide derivatives that can efficiently bind to Cl^−^ and exhibit potent Cl^−^ transport in model liposomes demonstrate potent antimicrobial activity through a combination of disrupted chloride homeostasis and membrane disruption.

## Graphical abstract

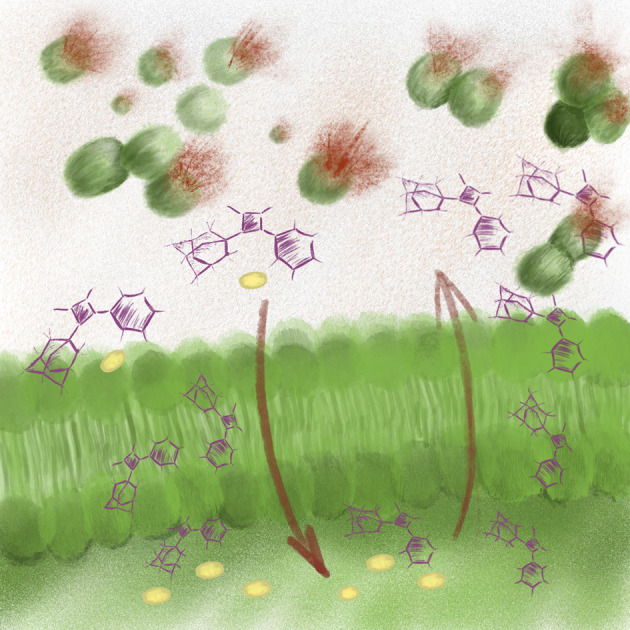

## Introduction

Antimicrobial resistance (AMR) is often referred to as a silent pandemic, and is the most serious threat to medicine in the modern age.^1–5^ Despite its lack of publicity, the global plight of antimicrobial resistance is expected to cause upwards of 10 million deaths annually, by 2050.^6^ Therefore, there is an urgent need for antimicrobial drugs that exhibit a distinct mechanism of action than those currently in circulation.

As the fundamental field of host–guest chemistry matures,^7^ research interest in its applications, particularly in the medicinal chemistry of supramolecular systems, continues to increase – most notably in the utilisation of anion transport in the development of novel therapeutics.^8–12^ Anionophores have seen a multitude of applications in the medicinal chemistry space, from their use in the development of therapeutics for cystic fibrosis pathologies,^13^ to disruptors of anion homeostasis *in cellulo*.^14–16^ Gale and co-workers have concentrated considerable effort to the development of anionophores that exhibit interesting biological activity. This focus on the medicinal chemistry of anion transporters has given rise to numerous examples of highly active compounds, ranging from squaramides that disrupt autophagy through alteration of lysosomal pH, leading to cell death,^17^ to 1,8-naphthalimide-urea conjugates with potent cytotoxicity.^18^ Despite the clear utility of supramolecular chemistry in the development of novel therapeutics, there has been little focus on the application of host–guest chemistry in the development of novel antimicrobials. This is somewhat surprising, due to the use of, and knowledge of the antimicrobial activity of natural product cation transporters such as monensin, salinomycin, valinomycin, and lasalocid.^19,20^ In this regard, there have been recent reports of compounds demonstrating antimicrobial activity, and have also had their anion transport capabilities explored.^21–26^ However, it is of note that the intricacies of the mode of action (MoA) of these compounds were not fully elucidated in these reports, and while there appears to be a link between anionophorism and antimicrobial activity, a direct correlation had not been established.

More recently, Busschaert and co-workers reported a urea-based anion transporter, exhibiting high levels of antimicrobial activity. In an effort to elucidate the mechanism of action, bacterial cytological profiling was employed as a high throughput method for the study of the MoA, which was concluded to most likely be as a result of anion transport.^27^ This constitutes a major step towards rational design of antimicrobial anionophores. Following this, our group reported the synthesis of a series of four “Squindoles” that demonstrate potent anion transport through CH–NH bonding and high levels of antimicrobial activity against *S. aureus* and MRSA, and indeed the ability to evade resistance over multiple generations.^28^ Using a combination of high-throughput Omics platforms, and chemical biology tools, we were able to verify the link between anion transport and antimicrobial activity, while also providing evidence to suggest additional modes of action.

In addition to this report of squaramide-based antimicrobials, our group has utilised this functionality in the development of anion receptors, sensors, and transporters.^28–33^ Squaramides are particularly attractive in this regard for their synthetic accessibility,^12^ strong hydrogen-bond donating ability,^34^ planar structure, and for the proposed increase in aromaticity upon ion binding providing a thermodynamic driving force for recognition.^35^ Indeed, recent interest in the cyclobutene derivative has significantly increased with a multitude of applications being reported across the chemical sciences.^12^

**Scheme 1:**
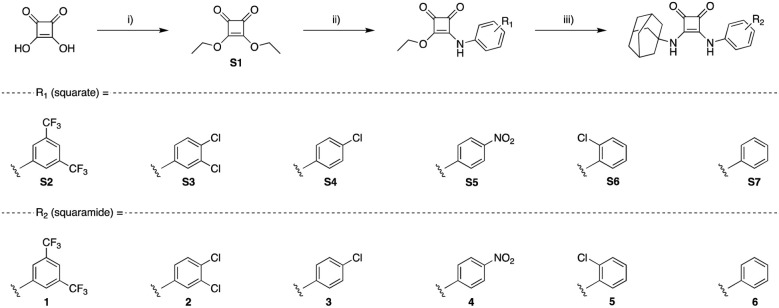
Synthetic pathways towards adamantyl-squaramides. Reagents and conditions: (i) triethyl orthoformate, EtOH, reflux, 72 h, 90%; (ii) substituted aniline, Zn(OTF)_2_ (20 mol%), EtOH, rt, 24 h, 23–79%; (iii) adamantylamine, TEA, EtOH, rt, 24 h, 21–67%.

**Fig. 1:**
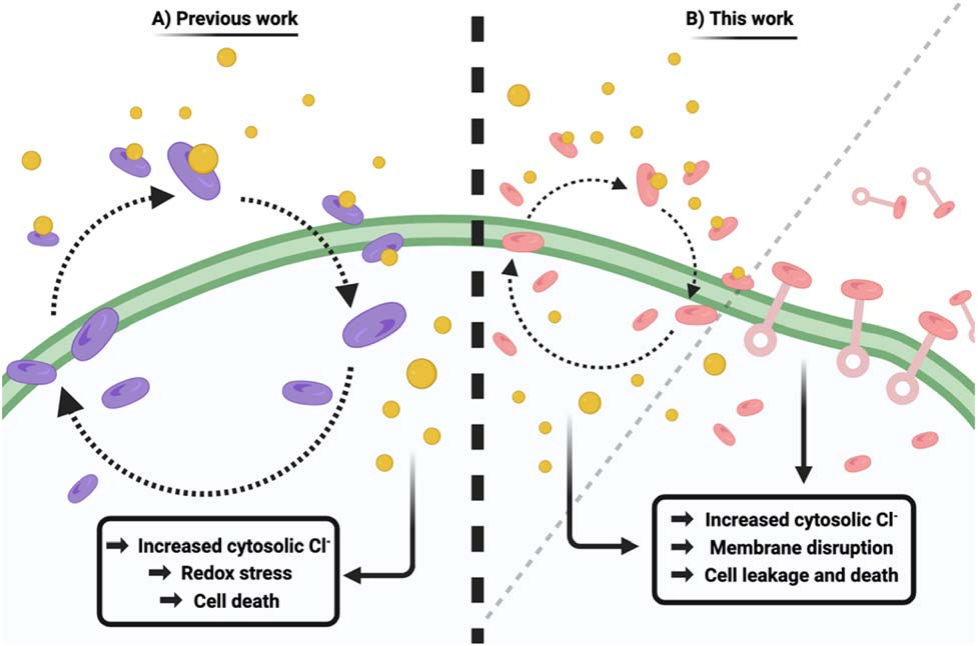
Schematic illustrating previous and current approaches to supramolecular antimicrobial development. (A) “Squindole” anion transporters can carry out anion transport “*in cellulo*” resulting in potent antimicrobial activity through increasing cytosolic Cl^−^ concentration, which yields redox stress and ultimately cell death. (B) Adamantyl-squaramides act as antimicrobials through a combination of anion transport, and disruption of membrane integrity.

In this work we report a series of structurally simple squaramides (1–6, Scheme 1) that can efficiently bind to Cl^−^ and exhibit potent anion transport *in cellulo*. This class of adamantyl-squaramides differ in their aromatic substitution patterns, in an effort to further unravel the contributions of aniline substitution to their antimicrobial activity, whilst all containing an adamantyl moiety to yield a higher lipid partitioning ability. These compounds, synthesised through a facile protocol, demonstrate antimicrobial activity against Gram-positive bacteria, and are observed to be non-toxic *in vivo*. In order to further increase our understanding of the underlying chemical biology, we have also synthesised two fluorescent analogues (7 and 8, Fig. 5), and used a range of assays to better understand the mechanism of action. Our results suggest a combination of anion transport and membrane disruption are responsible for the potent antimicrobial effect (Fig. 1).

## Results and discussion

### Synthesis of receptors 1–6

Adamantyl squaramides 1–6 were accessed through a divergent synthetic approach (Scheme 1), where the synthesis diverges in steps toward *N*-phenyl derivatives. Initially, diethyl squarate (S1) was reacted with the appropriate aniline in the presence of the Lewis-acid catalyst Zn(OTf)_2_, to afford each *N*-aryl squarate S2–S7 in yields ranging from 23–79%, depending on the steric or electronic influence of substitution patterns of the aniline. 1–6, the desired *N-*aryl adamantyl squaramides were successfully isolated through a nucleophilic substitution reaction of the respective squarate, and adamantylamine, where the isolated yields also varied across the series depending on the steric and electronic influences of the aniline substituents. The successful synthesis of each compound under study was confirmed in all cases by ^1^H, ^13^C NMR, and high-resolution mass spectrometry (HRMS) (see ESI† for further details).

### Anion binding and anion transport behaviour

**Fig. 2:**
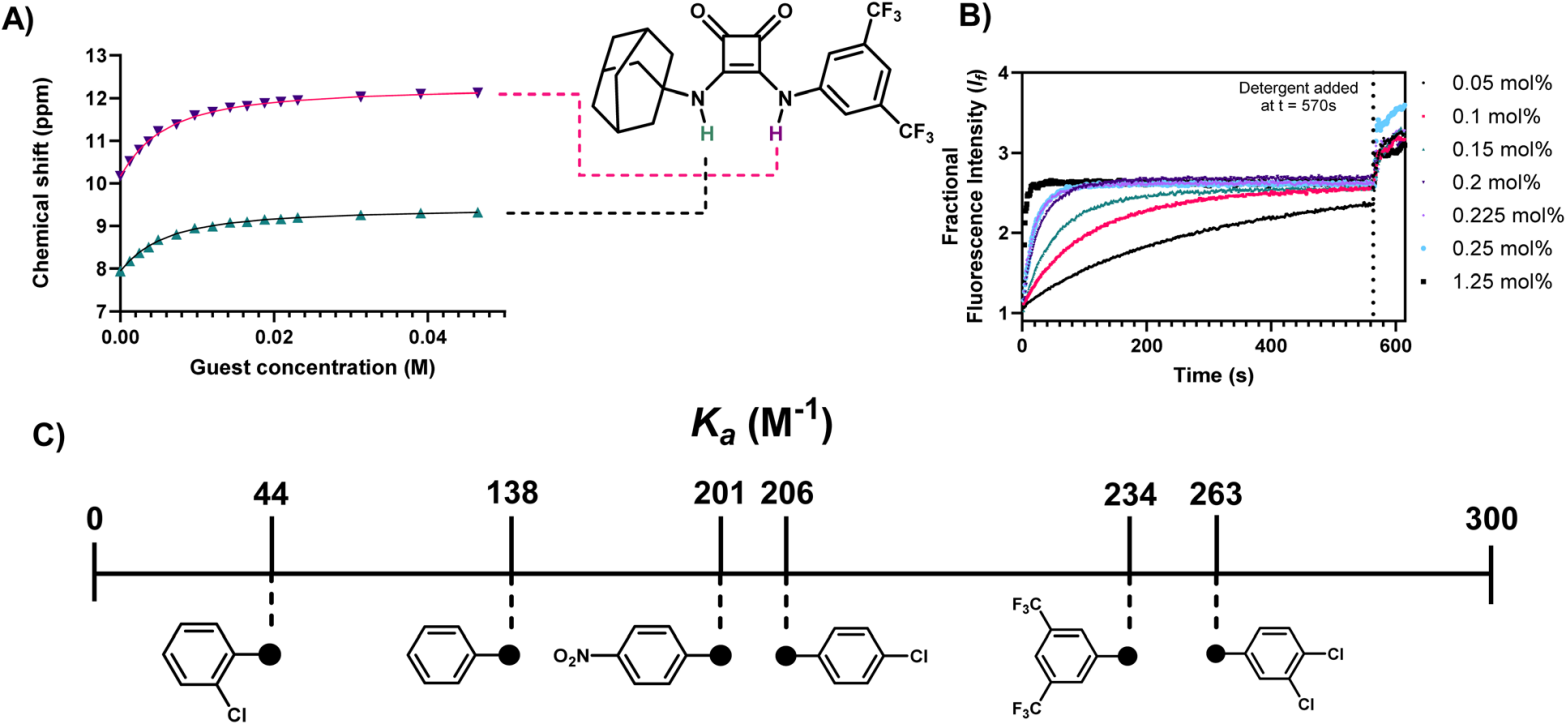
Cl^−^ binding and transport results for 1 and a schematic summary of overall binding propensities for each receptor. (A) ^1^H NMR Fitted binding isotherm for the titration of 1 (2.5 mM) in the presence of increasing concentrations of Cl^−^ (0–22 eq., TBACl) in DMSO-*d*_6_/0.5% H_2_O where data is fitted to a 1 : 1 binding model and illustrates the migration of NH signals throughout the titration. Relevant protons constituting the binding cleft are annotated within the structure of 1; (B) results of the lucigenin Cl^−^/NO_3_^−^ exchange assay carried out in the presence of incrementally increasing concentrations of 1 (molar% relative to LUV's at a fixed concentration of 0.4 mM); (C) schematic summary of the overall binding affinities of each receptor.

To verify if 1–6 formed host–guest complexes with Cl^−^ in solution, ^1^H NMR titrations of each compound (2.5 mM) with TBACl were carried out in DMSO-*d*_6_. Increasing equivalents of Cl^−^ were incrementally added, with spectral information obtained upon addition of each molar equivalent of Cl^−^. After addition of 10 eq. of TBACl, each compound showed significant downfield shifts in protons correlating to the squaramide NHs, and additional minor shifts for *ortho*-aryl protons, for 1–6*.* Taking 1 as an example (Fig. 2), upon addition of sequential aliquots of Cl^−^ there is a clear downfield shift of both squaramide NHs, with minor contributions to the binding cleft derived from the *ortho*-aryl protons of the aniline of 1. The binding data from each titration was fitted to a 1 : 1 binding model using the *Bindfit*^36^ open access software, and the results are tabulated below (Table 1), alongside the respective Hammett constant for the relevant substitution pattern.^37^ The results clearly indicate the influence of the substitution pattern around the *N*-aryl substituent where, unsurprisingly, compounds 1–4 (with strongly electron withdrawing substituents) displayed the highest binding affinities. This aligns with previously reported receptors,^38^ where compounds containing CF_3_ substituents in particular are known to have increased anion binding affinity as well as transport ability.^39^

**Table 1:** Association constants and transport properties of compounds 1–6 towards Cl^−^ determined using ^1^H NMR spectroscopic titrations in 95% DMSO-*d*_6_, and Cl^−^/NO_3_^−^ exchange assays, respectively. For binding titrations, errors were below 10% in all cases, and all data was fitted to a 1 : 1 binding model using the *Bindfit* open access software^a^

Compound	Binding mode	*K*_a_ (M^−1^)	Hammett parameter (*σ*)^37^	EC_50_ (mol%)
1	1 : 1	235	0.86	0.084
2	1 : 1	263	0.6	0.01
3	1 : 1	207	0.23	0.260
4	1 : 1	202	0.78	0.031^a^
5	1 : 1	44	—	1.349
6	1 : 1	138	0.0	—

a Reported by Gale and co-workers.^38^

Gale and co-workers have recently reported a series of lipophilic adamantyl based anion transporters that were shown to successfully transport Cl^−^across prototypical POPC membranes, including 4.^38^ Given our objective was to use such transport behaviour as a method to disrupt anion homeostasis in bacteria, we also conducted anion transport assays in Large Unilamellar Liposomes (LUVs) using the well-established Lucigenin-based Cl^−^/NO_3_^−^ exchange assay.^40^ Briefly, LUVs containing the chloride sensitive dye lucigenin in a NaNO_3_ solution were prepared and a NaCl pulse was added to create a chloride concentration gradient. Upon addition of the putative transporter, the quenching of the fluorescence of lucigenin was monitored over time to assess Cl^−^ influx. Under these conditions, each receptor showed a concentration dependent capacity for Cl^−^ transport, with the exception of 6 (see ESI†), where upon fitting to the Hill equation, EC_50_ concentrations for Cl^−^ transport were resolved in most cases. These values (Table 1) show clear congruency with the obtained anion binding results, where the strongest binders (1–4) also showed the most efficient transport capacity with EC_50_ values ranging from 0.031 to 0.26 mol%.

### Antimicrobial susceptibility and toxicity testing

With the knowledge that each compound can bind to and transport Cl^−^ in LUVs (with the exception of 6), we sought to rapidly assess the potential antimicrobial activity of each compound. To do this, a zone of inhibition assay was used as a qualitative measure of activity. To do so, 5 μL of a 1 mM stock of the respective compound was pipetted atop nutrient agar which was previously spread with a panel of Gram-positive and -negative pathogens of interest. Following incubation at 37 °C for 24 h, zones of inhibition were measured and recorded for each compound. Zones with a radius ≤ 2 mm were deemed as not active, and thus eliminated from further study. Compounds 1, 2, 3, 4, and 5 all showed promising bioactivity against both Gram-positive and -negative pathogens and their activity was quantified, where possible, through determination of IC_50/80_ values, using a procedure in line with those established as standard by the Clinical and Laboratory Standards Institute (CLSI).

To determine IC_50/80_ values, growth (optical density) of *S. aureus*, methicillin-resistant *S. aureus* (MRSA), *P. aeruginosa*, and *K. pneumoniae* when treated with a range of concentrations of each active compound *in vitro* was measured at 600 nm after a 24 h incubation period at 37 °C. Each compound was studied for activity below 500 μM, and those showing little activity above 200 μM, were noted as such, and omitted from further study.

From these assays it became apparent that 3 compounds were highly active; 1, 2, and 4 (see Table 2). For 1, the most active compound against both pathogens of interest, an IC_50_ = 781 nM (*S. aureus*) & 1.56 μM (MRSA) was determined. Furthermore, for both pathogens a 200 μM concentration of 1 resulted in approximately 80% inhibition of bacterial growth, thus representing an IC_80_ value against both *S. aureus* & MRSA. Less potent were the remaining two lead compounds, 2 and 4, yet all still retained a measurable level of activity. The remaining IC_50_ values against *S. aureus* for 2, and 4 were determined to be 62.5 μM, and 170 μM, respectively. For each, the IC_80_ values for each are as follows; 250 μM for 2, and >500 μM for 4 (see ESI† for further details). It was also observed that 1, 2 and 4 retained a high degree of activity against MRSA, with varying IC_50/80_ values for each. 1 was less potent against MRSA, having an IC_50_ = 1.56 μM and an IC_80_ = 200 μM. Less potent was 2, which has an IC_50_ = 25 μM for MRSA but an IC_80_ value was not determined, as it exceeded the testing range. Compound 4 exhibited an IC_50_ = 50 μM against MRSA (IC_80_ lies above 200 μM) and still presented a high degree of activity against both pathogens (see ESI† for further details).

**Table 2:** Minimum inhibitory concentrations for compounds 1–6 as determined by growth inhibition assays. All IC_50/80_ concentrations (μM) represented were determined from mean (±SEM) percentage growth of bacteria relative to control. n.d = out of tested range (>200 μM). n.a = no observed activity

Compound	*S. aureus*		MRSA	
	IC_50_	IC_80_	IC_50_	IC_80_
1	0.781	200	1.6	200
2	62.5	250	25	n.d
3	n.d	n.d	n.d	n.d
4	170	n.d	50	n.d
5	n.a	n.a	n.a	n.a
6	n.a	n.a	n.d	n.d

Surprisingly, compound 3, which had shown efficient transport ability in LUVs, did not show potent antimicrobial activity in either strain. Despite some observed activity in the inhibition assays, the IC_50_ values for 3, 5 and 6 could not be determined as they lay outside the concentration range tested for each. Hence, these compounds were omitted from further study. Furthermore, despite promising qualitative antimicrobial results against both Gram-negative pathogens tested, the IC_50/80_ values could not be determined as they also lay outside the concentration range tested. We ascribe this limited activity to the presence of a secondary cell membrane in Gram-negative bacteria, which may inhibit the compounds capacity to enter the cell.

Next, to assess the medicinal validity of compounds 1, 2, and 4 we investigated the *in vivo* toxicity of these compounds using the *Galleria mellonella* model organism. This model has been introduced as an alternative model to study microbial infections as it can be easily and inexpensively obtained and doesn't require specialised lab equipment or impose significant ethical constraints. Moreover, although they lack an adaptive immune response, their innate immune response shows remarkable similarities with the immune response in vertebrates.^28,41–43^ Thus, *G. mellonella* larvae were dosed with a concentration of each compound at 10× IC_50_ and monitored for signs of toxicity or death over a 72 hour window. Following treatment with 1, 2, and 4, there were no appreciable signs of acute/chronic toxicity arising from any of the compounds tested. After 72 h, each larval group showed ordinary levels of movement, response to external stimuli, no visible signs of melanisation, or death, when compared to untreated control populations (see ESI†).

With the demonstrated high levels of antimicrobial activity of 1, 2, and 4, and this verification of *in vivo* non-toxicity, we believe this class of compound offers some promise in drug development and sought to elucidate whether the mechanism of action is directly related to the anion transport activity.

### Determining the mode of action

Given our previous finding that efficient anion transport can lead to potent antimicrobial effect^28^ and the above results that suggest that compounds 1, 2 and 4 are both efficient anion transporters and potent anti microbials, we next sought to ascertain if the mode of action was related to their anion transport behaviour and whether the observed transport behaviour in LUVs translated *in cellulo.*

**Fig. 3:**
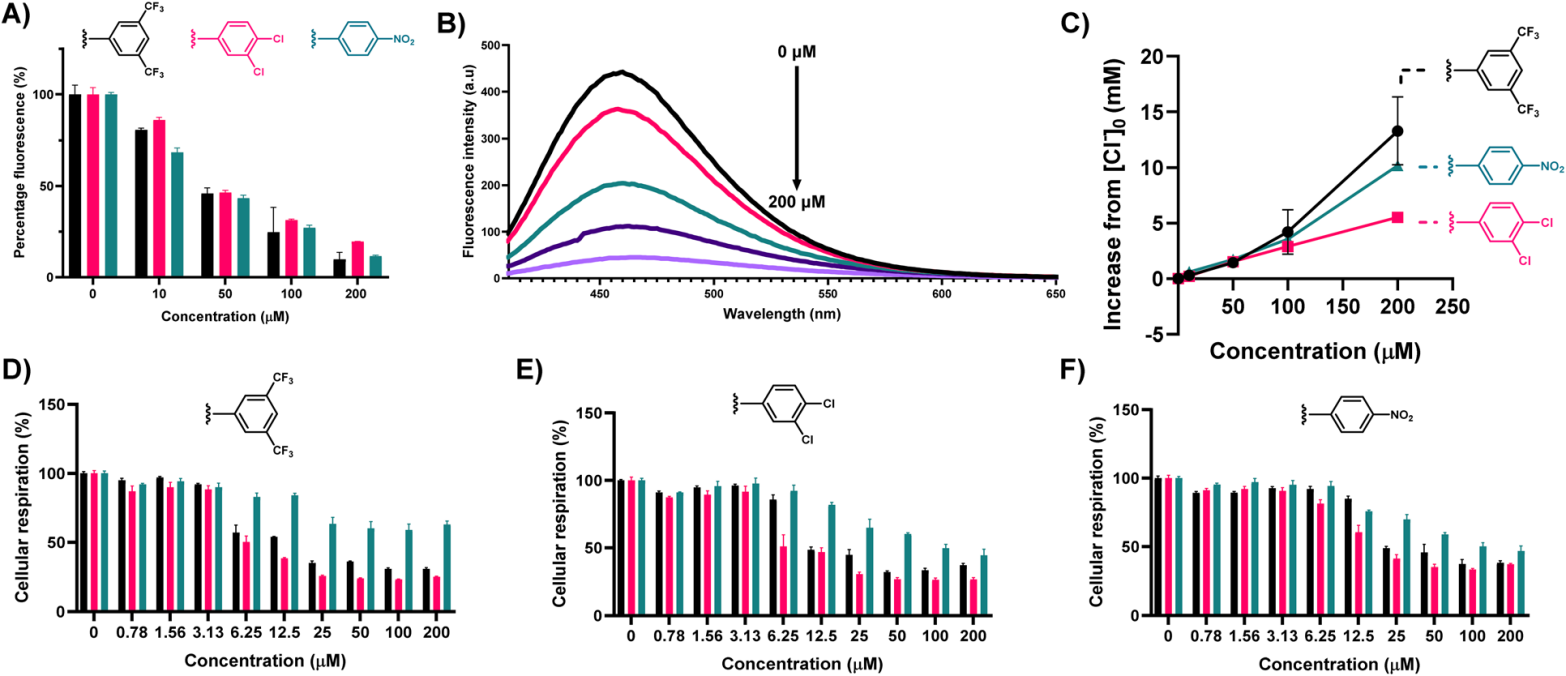
Compounds 1, 2, and 4 can effectively influx chloride into *S. aureus* cells, and removal of Na^+^ and Cl^−^ from solution does not alter the effect of 1, 2, and 4 on cellular respiration. (A) Percentage fluorescence of MQAE compared to control, when treated with a series of concentrations of each compound. Blue = 1, red = 2, green = 4; (B) fluorescence spectrum of MQAE upon addition of 1 at varying concentrations, illustrating the collisional quenching effect of chloride influx, mediated by 1; (C) plot of increasing Cl^−^ concentration from [Cl^−^]_0_, upon increasing concentration of 1, 2, and 4; (D) MTT assay monitoring the effect of 1 on cellular respiration in *S. aureus*; (E) MTT assay monitoring the effect of 2 on cellular respiration in *S. aureus*; (F) MTT assay monitoring the effect of 4 on cellular respiration in *S. aureus*. For MTT assays, black = HBSS buffer, red = Cl^−^ free HBSS buffer, green = Na^+^ free HBSS buffer.

To study this, we utilised *N*-(ethoxycarbonylmethyl)-6-methoxyquinolinium bromide (MQAE) as a fluorescent reporter for cellular Cl^−^ levels. MQAE fluorescence is highly sensitive to Cl^−^ flux, where increased cytosolic Cl^−^ concentration gives rise to a collisional quenching effect. We have previously demonstrated the utility of this technique for the study of anion transport mechanisms in *S. aureus*.^28^ To do this, cells were pre-treated with MQAE and then exposed to varying concentrations of 1, 2 and 4, whereafter MQAE fluorescence was read at *T* = 5 min, and plotted relative to control (Fig. 3).

In all cases, MQAE fluorescence was rapidly quenched, within a matter of minutes, in a concentration dependent manner. At all concentrations tested, clear decreases in the level of emission relative to untreated controls were observed. This clear reduction in MQAE emission provides clear evidence for the capacity of these compounds possessing antimicrobial activity to transport Cl^−^*in cellulo*, and is largely congruent with results obtained in LUV's.

Furthermore, using the fluorescence data acquired from these assays, and fitting to the Stern–Volmer equation, we could approximate the increased concentration of Cl^−^ with respect to basal fluorescence for each of the concentrations studied (Table 3).

**Table 3:** Summary of the increase in Cl^−^ concentration upon addition of 1, 2, and 4 at varying concentrations, determined through fitting MQAE fluorescence to the Stern–Volmer equation

Compound	Increase in chloride concentration from basal level [Cl_*θ*_] (mM)			
	10 μM	50 μM	100 μM	200 μM
1	+0.31 (±0.02)	+1.44 (±0.16)	+4.22 (±2.01)	+13.31 (±3.05)
2	+0.21 (±0.02)	+1.53 (±0.07)	+2.91 (±0.02)	+5.49 (±0.01)
4	+0.61 (±0.07)	+1.74 (±0.02)	+3.57 (±0.18)	+10.19 (±0.08)

Each of the compounds tested could effectively increase the cellular chloride concentration within 5 min of treatment by up to 13.31 mM (+[Cl_*θ*_]), which is a dramatic increase, and despite the relative osmotolerant nature of *S. aureus*,^44^ where intracellular Cl^−^ concentration can reach 152.9 mM,^45^ this provides a rationale for the observed level of activity for each. Such a dramatic increase (in a short time) in cellular chloride concentration as a result of the activity of these compounds is likely an arbitrator of the observed inhibitory effect.

To further probe the relationship between anion homeostasis and antimicrobial effect, a modified MTT assay was used next to monitor disruption of cellular respiration in the presence of 1, 2, or 4 either with abundant Cl^−^ and Na^+^, in the absence of Cl^−^, or in the absence of Na^+^ (Fig. 3). Surprisingly, in the absence of either Cl^−^ or Na^+^ for 1, 2, and 4 - their removal did not reduce activity to any large extent. In all cases, a concentration dependent decrease in cellular respiration occurred regardless of whether there is an abundance of Cl^−^, Na^+^, or neither. Whilst this result was surprising, it suggested that there may be additional mechanisms of action unrelated to anion transport. Chew and co-workers have reported on the behaviours, and dynamics of adamantylamines in the lipid bilayers, and discussed that adamantylamines show a preferential accumulation in the interfacial layer of lipid bilayers^46^ and thus we suspected that these compounds, with an identical pharmacophore may behave in a similar manner accumulating in lipid bilayers of bacterial membranes.^47,48^

**Fig. 4:**
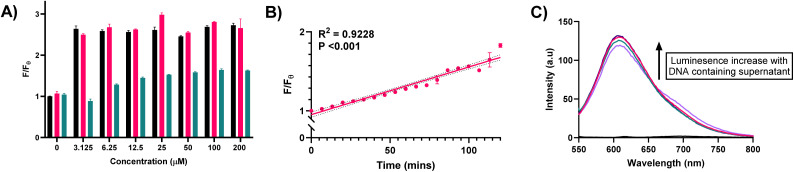
Determination of the ability of xompounds 1, 2, and 4 to disrupt the membrane integrity of *S. aureus.* (A) Quantification of propidium iodide fluorescence from *S. aureus* cells upon treatment with various concentrations of 1, 2, and 4. Black = 1, red = 2, green = 4; (B) graph of propidium iodide fluorescence from *S. aureus* cells over time upon treatment with 50 μM 1, with fitted linear regression of mean data points, with 95% CI shown (dotted line); (C) DNA leakage assay quantified by Ru(Phen)_2_(DPPZ)Cl_2_ luminescence turn-on in culture supernatant. Black = sterile nutrient broth (baseline signal), purple = no treatment, pink = 100 μM 1, green = 100 μM 2, violet = 100 μM 4.

To study whether compounds 1, 2, and 4 do indeed accumulate in the membrane, and yield a depolarising effect, or compromise its integrity we utilised propidium iodide (PI), a red-emissive (*λ*_max_(em) = 617 nm) cell-impermeable fluorophore. PI undergoes a fluorescence increase upon internalisation and DNA intercalation, and thus under ordinary circumstances PI fluorescence is quenched, but if membrane integrity is compromised, a fluorescence “turn-on” occurs. After two hours of treatment with 1, 2, and 4, PI fluorescence was compared to basal fluorescence from the fluorophore. In the case of 1, and 2 emission centred around 615 nm was greatly increased irrespective of concentration, indicating that membrane perturbation is rapid and effective for these compounds (Fig. 4A). This clear membrane depolarising effect was less obvious for 4, and follows the trend of the observed transport, and toxicity results. However, it is of note that each compound can effectively promote an influx of PI through membrane depolarisation.

To further investigate the rate of this phenomenon, PI fluorescence was monitored as a function of time, upon treatment of *S. aureus* with 1 at a concentration of 50 μM (Fig. 4B). Over the course of two-hours, it can clearly be observed an increase in the fluorescence of PI, as a result of internalisation promoted by membrane depolarisation, likely caused by 1.

In addition to assays monitoring the uptake of PI, luminescence assays to monitor the release of cellular components such as DNA/RNA were also undertaken. Using Ru(phen)_2_(dppz)Cl_2_, which acts as a highly sensitive, “off-to-on” emissive probe for nucleic acids (NA),^49–51^ we assessed the levels of NA release from cells upon treatment with 1, 2, and 4 (100 μM) by comparing emission intensity of the complex in the absence of nucleic acids, to untreated controls, and treated samples (Fig. 4C). When Ru(phen)_2_(dppz)Cl_2_ was added to sterile nutrient broth, which lacks nucleic acids, emission from the ^3^MLCT band (*ca.* 620 nm) is highly quenched, however, when added to culture supernatant in the absence of treatment with 1, 2, or 4 we see a large increase in ^3^MLCT derived luminescence indicating the presence of nucleic acids in the solution. This initial luminescence increase is unsurprising, as *S. aureus* is known to release DNA under active growth conditions, in the form of eDNA,^52^ which acts as a chemical messenger for microbial species.^53^ However, importantly, luminescence was further increased in cultures pre-exposed to 1, 2 and 4 for 1 h at 100 μM. This additional increase in luminescence indicates a heightened level of extracellular NA and may be another indication of membrane disruption. While this increase from control is minor, it provides sufficient evidence to suggest the release of NA upon treatment. Indeed, a significant increase in luminescence would not be expected, as the release of large architectures such as those observed for nucleic acids is slow, due to the bulk of the biomolecules. If a sharp, fast increase in luminescence was observed, this would instead implicate cellular lysis in the mechanism of action, which does not appear to be the case.

### Utilising fluorescent probes to study cellular uptake

**Fig. 5:**
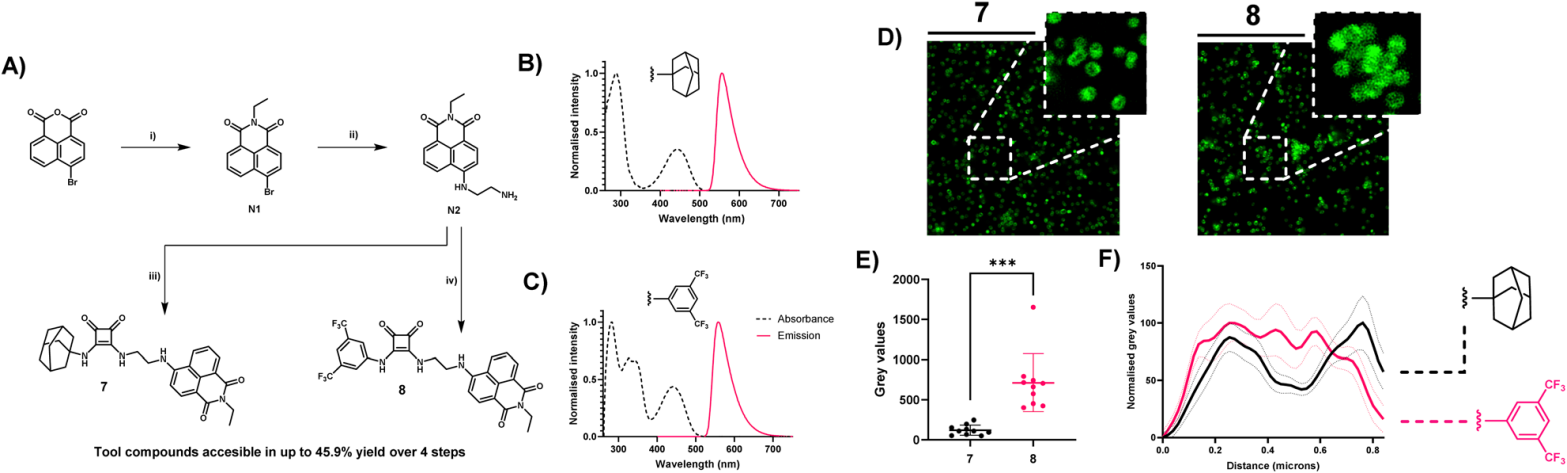
(A) Synthesis and photophysical characteristics of compounds 7 and 8. Reagents and conditions: (i) ethylamine hydrochloride, 1,4-dioxane, reflux, 18 h, 90%; (ii) 1,2-diaminoethane, toluene, reflux, 18 h, 75%; (iii) S8, TEA, EtOH, reflux, 18 h, 68%; (iv) S2, TEA, EtOH, reflux, 18 h, 65%; (B) normalised excitation and emission spectrum of compound 7; (C) normalised excitation and emission spectrum of Compound 8. (D) stimulated emission depletion nanoscopy (STED) analysis of the cellular distribution of 7 and 8 derived fluorescence in *S. aureus*. STED images are of *S. aureus* treated with 3 μM 7 or 8 for 30 min, with an overlay of a zoomed representative region of interest (ROI) illustrating cellular distribution of 7 in single cells (E) mean grey value abundance of 7 and 8 at the centre of single cells (0.5 μm) previously identified as ROI's. (F) Normalised mean grey value distribution of both 7 (black) and 8 (red) within ROI's, which were identified as single cells. Mean grey values are represented as mean distribution ± SEM of 10 individual cells selected at random from three distinct nanoscopy images acquired from differing regions of the slide.

In an effort to further underpin the apparent membrane compromising nature of 1, 2, and 4, we sought to ascertain if preferential accumulation of similar compounds occurs on the cellular periphery – at the cell membrane, or indeed in other sub cellular compartments. To study this phenomenon, two additional compounds were synthesised (7 and 8; Fig. 5). Each of these compounds differ from 1, their parent compound, through incorporation of a fluorescent reporter group, replacing either the adamantyl or 3,5-bis(trifluoromethyl)phenyl motif. The intention here was to elucidate if these motifs tailor the activity, or yield a sub cellular directionality, as we hypothesised due to the activity of 1. For the fluorescent handle, the 1,8-naphthalimide scaffold was chosen due to its minimal structural obstruction to the central pharmacophore and minimal participation in host–guest association complexes.^54,55^ Furthermore, this motif was chosen for its favourable emissive characteristics, in the context of fluorescence imaging where it has also recently been exploited for subcellular targeted anion transporters.^56^ Moreover, with particularly large stokes shifts, and resistance to photobleaching, these generally cell-permeable motifs have many of the desired characteristics required for microscopy.^57^

The syntheses of 7 and 8 were achieved in 3–4 steps (Fig. 5(A)), from 4-bromo-1,8-naphthalic anhydride where the appropriate *N*-ethyl derivative (N1) was formed prior to reaction with ethylene diamine to produce N2. Subsequent reaction with either S2 or S8 (see ESI†) led to the successful formation of 7 and 8 in 68–65% yield, respectively. The full details of synthetic procedures and characterisation are provided in the ESI.† With 7 and 8 in hand, their absorption and emission properties were evaluated in aqueous solution (10 μM) where both compounds exhibited broad absorbance bands characteristic of 1,8-naphthalimides with maxima at ∼450 nm (Fig. 5(B)). Similarly, the fluorescence emission spectra confirmed green emission also characteristic of 1,8-naphthalimides centred at ∼550 nm and confirmed their utility for use in fluorescence microscopy (Fig. 5(C)). Thus, each compound was used to stain *S. aureus* cells by treatment (3 μM) for 30 min, before the cells were fixed, and slides were prepared for super-resolution nanoscopy. STimulated-Emission Depletion (STED) nanoscopy was used to analyse the cellular fate of 7 and 8 where the results clearly showed that incorporation of the adamantyl group yielded a membrane accumulation of 7. Comparing single cell nanoscopy images for those stained with 7 to those stained with 8, it is visually apparent the membrane accumulation occurs for 7 while more uniform fluorescence intensity across the cells is observed for 8 (Fig. 5(D)–(F)). Single *S. aureus* cells could be easily identified using this technique, and with a minimal resolution limit of approximately 20 μm,^58^ the intricacies of fluorescence localisation could easily be identified. Indeed, when these intensity values were plotted across the diameter of single cells, it was apparent that fluorescence intensity from the centre of cells was decreased for 7, when compared to the intensity of 8 in the same region. When intensity values at the centre of cells were directly compared, a significantly lower intensity for 7 was observed, compared to 8 (*p* > 0.001) further confirming the propensity of the adamantly derivatives to accumulate at the membrane of *S. aureus* cells.

## Conclusions

In summary, a series of structurally analogous adamantyl squaramides have been designed, synthesised and characterised in addition to their chloride recognition and transport behaviour being studied using a combination of spectroscopic and photophysical measurements. The original goal of the work was to link anion transport behaviour and antimicrobial efficacy, however, while these compounds have been shown to effectively bind and transport anions and can also display antimicrobial activity, a comprehensive study of the mechanism of action has shown that three of the most active compounds; 1, 2, and 4 act through two distinct mechanisms. Using an array of chemical biology techniques, we have illustrated that these non-toxic compounds display antimicrobial activity both through transporting Cl^−^ across biological membranes, and by disrupting the membrane stability through accumulation at the cellular frontier.

MTT assays indicated additional modes of action, and the uptake and release of otherwise membrane impermeable compounds and biomolecules confirmed it. In the final section of the paper, two additional fluorescent reporter compounds were synthesised to probe the cellular fate of the adamantly anionophores, and the resulting super-resolution nanoscopy experiments rationalised the mechanism of action of these compounds to likely be a combination of anion transport and membrane disruption. This work builds upon foundational results in the development of rationally designed supramolecular receptors as antimicrobial agents and provides further key insights that may aid in the rational design of new anti-infective compounds. Moreover, the work also provided novel fluorescent reporter compounds that can be used to study the subcellular fate of such receptors in bacteria. We continue to explore this area and are working towards the targeted delivery of supramolecular receptors to specific bacterial strains.

## Data availability

All relevant experimental details, characterisation data and binding titration data are presented in the main text and ESI.† The raw titration data is available for download at http://app.supramolecular.org/bindfit/.

## Author contributions

R. B. P. E, K. K., and L. E. B conceived and designed the study, and wrote the manuscript. R. B. P. E and K. K. supervised the study. L. E. B. synthesised the compounds under study, carried out spectroscopic titrations, performed all biological experiments, and carried out all statistical analyses. X. L. and F. M. carried out lucigenin assays. All authors discussed the results and commented on the manuscript.

## Conflicts of interest

There are no conflicts to declare.

## Supplementary Material

SC-016-D4SC01693A-s001
