# Supplementary material for: Uncovering the potent antimicrobial activity of squaramide based anionophores – chloride transport and membrane disruption
Source: Chem Sci. 2025 Jan 22;16(9):4075–84. doi: 10.1039/d4sc01693a (PMC11788822; doi:10.1039/d4sc01693a)
Supplement: SC-016-D4SC01693A-s001 [file SC-016-D4SC01693A-s001.pdf]

Supplementary Information (SI) for Chemical Science.
This journal is © The Royal Society of Chemistry 2025

Uncovering the potent antimicrobial activity of squaramide based anionophores – chloride transport and membrane disruption

Luke E. Brennan,[a,b]† Xuanyang Luo,[a] Farhad Ali Mohammed,[a] Kevin Kavanagh[b,c] and Robert B. P. Elmes *[a,b,d]

[a] Department of Chemistry, Maynooth University, Maynooth, Co. Kildare, Ireland.

[b] Synthesis and Solid-State Pharmaceutical Centre (SSPC), Ireland.

[c] Department of Biology, Maynooth University, Maynooth, Co. Kildare, Ireland.

[d] Kathleen Lonsdale Institute for Human Health, Maynooth University, Maynooth, Co. Kildare.

† Current address: Leibniz-Forschungsinstitut für Molekulare Pharmakologie (FMP), Robert-Rössle-Strasse 10, Berlin, Germany.

*robert.elmes@mu.ie

Supplementary Information:

General experimental

Commercial materials were supplied by TCI Europe, Fluorochem Ireland or Sigma Aldrich and were used without further purification. HPLC grade solvents were used as received. Anhydrous solvents were used as received (DMF, THF, 1,4-dioxane) or generated by storage over molecular sieves under an atmosphere of N_2 (3/4 Å – DMF, MeCN, THF, 1,4-dioxane). Anhydrous DCM was generated by distillation over CaH, and was generated as needed. ^{1}H NMR spectra were recorded using a Bruker Avance III 500 at a frequency of 500.13 MHz, and

are reported as parts per million (ppm) with $CDCl_3$ (δH 7.26 ppm) or DMSO-d_6 (δH 2.50 ppm) as an internal reference. The data are reported as chemical shift (δ), multiplicity (br = broad (prefix), s = singlet, d = doublet, t = triplet, q = quartet, dd = doublet of doublets, td = triplet of doublets, dt = doublet of triplets, m = multiplet), coupling constant (*J*, Hz) and relative integral. ^{13}C NMR spectra were recorded using a Bruker Avance III 500 at a frequency of 125 MHz and are reported as parts per million (ppm) with $CDCl_3$ (δH 77.1 ppm) or DMSO-d_6 (δH 39.5 ppm) as an internal reference. High resolution ESI spectra were recorded on an Agilent 6310 LCMS TOF. Analytical TLC was performed using pre-coated silica gel plates (Merck Kieselgel 60 F254), and was visualised via UV, or with an appropriate TLC stain (e.g. Ninhydrin, $KMnO_4$, etc.). Flash chromatography was performed using silica gel 40-63 µM, 60 Å. All compounds were loaded to the flash column as a solute in the less polar solvent unless otherwise stated. Infrared (IR) spectra were obtained *via* ATR as a solid on a zinc selenide crystal in the region of 4000 – 400 cm^{-1} using a Perkin Elmer Spectrum 100 FT-IR spectrophotometer. Microwave (MW) experiments were carried out in sealed vessels in a CEM Discovery MW, with transversal IR sensor for reaction temperature monitoring. UV-visible spectroscopy measurements were made at 25 ºC on a Lambda 365 Perkin Elmer UV-vis spectrophotometer. Fluorescence emission spectra were performed at 25 ºC and 37 ºC on an Agilent Spectrofluorometer equipped with a 450 W xenon lamp for excitation. Starna and Hellma quartz cuvettes of 1 cm path length and several volumes were employed. All biological experiments were carried out using pre-sterilised materials, or were sterilised prior to use. All measurements taken in 96-well format were done so using a BMG Labtech Clariostar Plus plate reader.

Synthetic schemes:

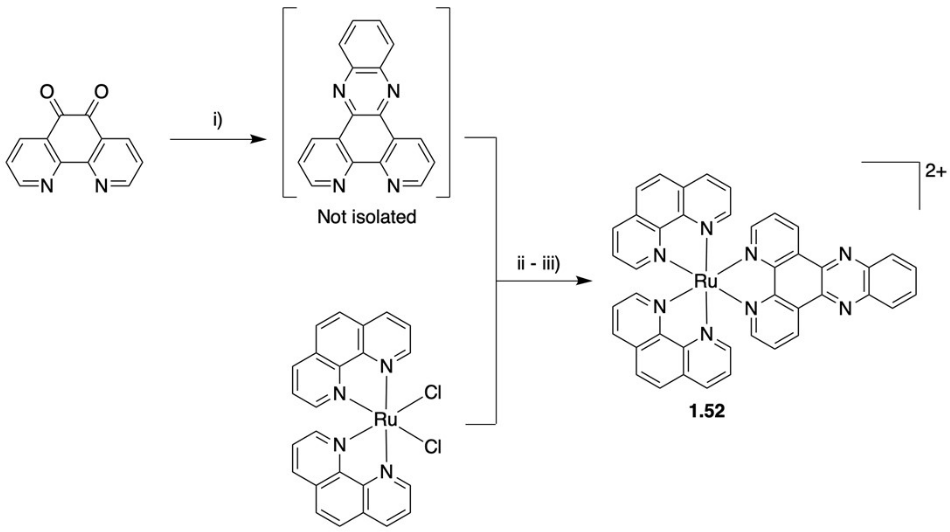

Scheme S1. the synthesis of Ru(Phen)$_2$(dppz)Cl$_2$. *Reagents and conditions*: i) *ortho*-phenylene diamine, *para*-toluenesulfonic acid, EtOH, reflux, 4 hr, telescoped; ii) H_2O/EtOH (1:1), MW, 140 ^oC, 40 mins, TBAPF$_6$, 28%; iii) Amberlyst-Cl ion exchange resin (excess), MeOH, rt, 1 hr, quant.

<u>Synthetic methods:</u>

3,4-diethoxyl-cyclobut-3-ene-1,2-dione (S1):

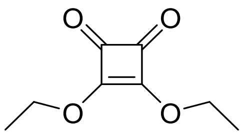

2,3-dihydroxy-cyclobut-3-ene-1,2-dione (10 g, 87.6 mmol) was dissolved in EtOH (90 mL) and refluxed at 80˚C for three hours. The solvent was subsequently removed *in vacuo*. The resultant white deposit was redissolved in a fresh portion of EtOH (90 mL) and refluxed for a further hour. The solvent was once more removed *in vacuo*, and the same procedure was repeated 4 times to afford a crude yellow slurry. The crude product was purified by column chromatography, using a 0 - 2% EtOH:DCM gradient as eluent to afford desired product as a yellow oil in a 90% yield. All spectral data is in good agreement with literature.[1]

3-ethoxy-4-(3,5-bis(trifluoromethyl)phenyl)amino-cyclobut-3-ene-1,2-dione (S2):

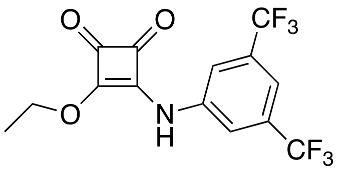

Compound **S2** was synthesised according to established procedures, from 3,5-bis(trifluoromethyl)aniline, and **S1**, and all spectral data shows good agreement with literature.[2]

3-(3,4-dichlorophenylamino)-4-ethoxy-cyclobut-3-ene-1,2-dione (S3):

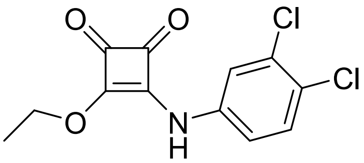

S1 (796 mg, 4.5 mmol, 1 eq) was suspended in EtOH (10 mL), followed by the addition of $Zn(OTf)_2$ (328 mg, 0.9 mmol, 0.2 eq). Once dissolved, a solution of 3,4-dichloroaniline (800 mg, 4.95 mmol, 1.1 eq) in EtOH (5 mL) was added to the reaction vessel and allowed to stir overnight at room temperature. The afforded precipitate was isolated via vacuum filtration and washed extensively with cold EtOH, and Et_2O to afford the title compound as a pale-yellow solid in a 29% yield. All spectral data is in good agreement with literature.[3]

3-(4-chlorophenyl)-4-ethoxy-cyclobut-3-ene-1,2-dione (S4):

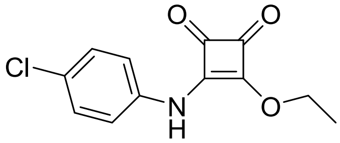

S1 (231 μL, 0.86 mmol, 1 eq), and $Zn(OTf)_2$ (63 mg, 0.172 mmol, 0.2 eq) were dissolved in EtOH (10 mL). To this was added, a solution of *p*-chloroaniline (120 mg, 0.946 mmol, 1.1 eq) in EtOH (2 mL). The resultant reaction mixture was allowed to stir at room temperature for 18 hr whereafter the solvent was removed *in-vacuo* to afford a crude slurry. Following extensive trituration with Et_2O the title compound was resolved as a beige solid in a 79% yield. All spectral data is in good agreement with literature.[4]

3-(4-nitrophenylamino)-4-ethoxy-cyclobut-3-ene-1,2-dione (S5):

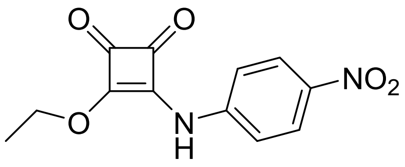

S1 (750 mg, 4.5 mmol, 1 eq) and $Zn(OTf)_2$ (328 mg, 0.9 mmol, 0.2 eq) were dissolved in EtOH (15 mL). To this was added dropwise, a solution of *p*-nitroaniline (697 mg, 5 mmol, 1.1 eq) in EtOH (10 mL). The resultant mixture was stirred at room temperature for 18 hr whereafter the precipitate was isolated via vacuum filtration, washed with cold EtOH, and dried under a stream of N_2 to afford the title compound as a bright orange solid in a 65% yield. All spectral data is in good agreement with literature.[2]

3-(2-chlorophenylamino)-4-ethoxy-cyclobut-3-ene-1,2-dione (S6):

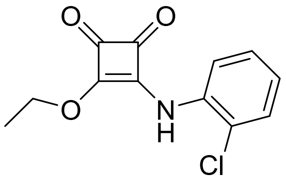

S1 (350 mg, 2.05 mmol, 1 eq), and $Zn(OTf)_2$ (149 mg, 0.41 mmol, 0.2 eq) were dissolved in EtOH (10 mL). To this was added 2-chloroaniline (0.35 mL, 2.2 mmol, 1.1 eq), dropwise. The resultant mixture was allowed to stir at room temperature for 72 hr, whereafter the solvent was removed *in-vacuo*, to afford a brown slurry. This slurry was resuspended in a minimal amount of MeCN, and placed in a sealed vessel containing Et_2O (3x v/v MeCN), to facilitate the formation of crystals. Once an adequate amount of isolable material was afforded (24 hr), the mother liquor was decanted, and the precipitate was washed with cold Et_2O, to afford the title compound as a yellow crystalline solid in a 23% yield. **^{1}H NMR** (500 MHz, DMSO) δ 10.67 (s, 1H), 7.53 (d, *J* = 7.8 Hz, 1H), 7.37 (dd, *J* = 4.9, 1.0 Hz, 2H), 7.32 – 7.26 (m, 1H), 4.69 (q, *J* = 7.1 Hz, 2H), 1.35 (t, *J* = 7.1 Hz, 3H). **^{13}C NMR** (126 MHz, DMSO) δ 188.3, 184.9, 178.9, 171.2, 134.6, 130.1, 128.0, 126.9, 69.8, 16.0.

3-ethoxy-4-phenylamino-cyclobut-3-ene-1,2-dione (S7):

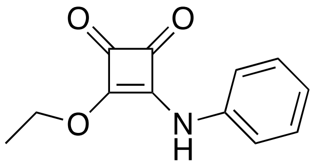

Compound **S7** was synthesised according to established procedures from **S1** and aniline, to afford the title compound, which showed good spectral agreement with literature.[2]

3-(tricyclo[3.3.1.1(3,7)]decyl-1-amino)-4-ethoxy-cyclobut-3-ene-1,2-dione (S8):

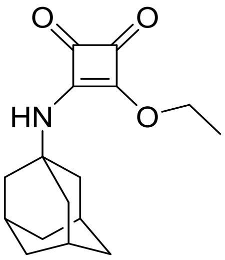

S1 (2.1 g, 12.3 mmol, 1 eq) was suspended in EtOH (30 mL). To this was added, 1-adamantylamine (2.23 g, 14.76 mmol, 1.2 eq) dissolved in EtOH (30 mL), dropwise over 5 mins. This solution was allowed to stir at room temperature for 24hr. The formed precipitate was isolated via vacuum filtration & washed with 3 x 2 ml portions of EtOH to afford the desired product as a white solid in a 78% yield. All spectral data is in good agreement with literature.[5]

3-((3,5-bis-trifluoromethyl)-phenylamino)-4-(tricyclo[3.3.1.1(3,7)]decyl-1-amino)-cyclobut-3-ene-1,2-dione (1):

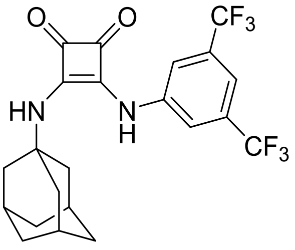

S2 (538 mg, 1.52 mmol, 1 eq), and $Zn(OTf)_2$ (112 mg, 0.31 mmol, 0.2 eq) were dissolved in EtOH (20 mL). To this was added 1-adamantylamine (252mg, 1.67 mmol, 1.1 eq), dissolved in EtOH (2 mL). This solution was added dropwise to the reaction vessel over a five-minute period. The reaction was stirred at room temperature for 24hrs. The resultant mixture was concentrated *in-vacuo* to resolve a yellow solid. The crude product mixture was redissolved in EtOAc (20 ml), washed with H_2O (3 x 30 ml), & dried over anhydrous $MgSO_4$, & concentrated partially *in-vacuo* to yield a White precipitate. The precipitate was isolated via filtration, washed with EtOAc & lyophilised, to afford the title compound as a white solid in a

45% yield. **^{1}H NMR** (500 MHz, DMSO) δ 10.15 (s, 1H), 8.08 (s, 2H), 7.92 (s, 1H), 7.69 (s, 1H), 2.13 (s, 3H), 2.00 (s, 6H), 1.67 (s, 6H).**^{13}C NMR** (126 MHz, DMSO) δ 183.4, 170.2, 163.8, 131.9 (q, C-CF_3), 129.4 (q, CF_3) 118.5, 60.2, 53.5, 42.8, 35.6, 29.4, 21.2, 14.5. **HRMS** (ESI-TOF), calculated for $C_{22}H_{20}F_6N_2O_2$, *m/z:* 459.15 $[M+H]^+$; found 459.1502, also found [M+Na]+; 481.1325.

3-(3,4-dichlorophenylamino)-4-(tricyclo[3.3.1.1(3,7)]decyl-1-amino)-cyclobut-3-ene-1,2-dione (2):

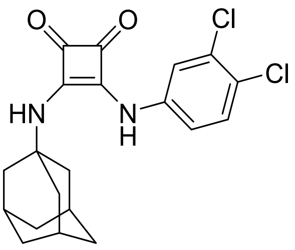

S3 (50 mg, 0.17 mmol, 1 eq), and $Zn(OTf)_2$ (10 mg, 0.03 mmol, 0.2 eq) were dissolved in EtOH (10 mL). 1-adamantylamine (30 mg, 0.19 mmol, 1.1 eq), dissolved inEtOH (2 mL) was added dropwise to the reaction mixture and was stirred at room temperature for 24hrs. The formed precipitate was isolated via vacuum filtration, with washings of cold EtOH, to resolve the title compound as a pale-yellow solid in a 58% yield. **^{1}H NMR** (400 MHz, DMSO) δ 9.82 (s, 1H), 7.89 (d, *J* = 2.7 Hz, 2H), 7.58 (d, *J* = 8.7 Hz, 1H), 7.32 (dd, *J* = 8.8, 2.7 Hz, 1H), 2.10 (d, *J* = 10.1 Hz, 3H), 1.98 (d, *J* = 2.6 Hz, 6H), 1.66 (s, 6H). **^{13}C NMR** (126 MHz, DMSO) δ 182.8, 179.8, 169.4, 163.8, 138.9, 131.7, 131.0, 123.8, 119.9, 118.2, 53.0, 42.5, 35.4, 28.8. **HRMS** (ESI-TOF), calculated for $C_{22}H_{20}Cl_2N_2O_2$, *m/z:* 391.02 $[M+H]^+$; found 391.0976, also found [M+Na]+; 413.0794.

3-(4-chlorophenylamino)-4-(tricyclo[3.3.1.1(3,7)]decyl-1-amino)-cyclobut-3-ene-1,2-dione (3):

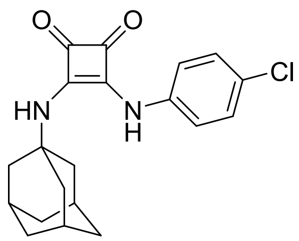

S4 (125 mg, 0.5 mmol, 1 eq), and $Zn(OTf)_2$ (44 mg, 0.1 mmol, 0.2 eq) were dissolved in EtOH (10 mL). To this stirring mixture was added adamantylamine (95 mg, 0.5 mmol, 1.1 eq), and the resultant mixture was allowed to stir at room temperature for 18 hr, whereafter the solvent was concentrated *in-vacuo*, to afford a crude deposit which was triturated extensively with Et_2O, to afford the title compound as a beige solid in a 56% yield. **^{1}H NMR** (400 MHz, DMSO) δ 9.69 (s, 1H), 7.86 (s, 1H), 7.49 (dd, *J* = 8.8 Hz, 2H), 7.38 (dd, *J* = 8.8 Hz, 2H), 2.10 (s, 3H), 1.98 (s, 6H), 1.65 (s, 6H). **^{13}C NMR** (101 MHz, DMSO) δ 169.2, 163.9, 137.9, 129.1, 126.5, 119.6, 52.7, 42.4, 35.2, 28.9. **IR** (ATR): ν_{max} (cm^{-1}) = 3231, 2907, 2849, 1787, 1670, 1621, 1603, 1563, 1522, 1498, 1459, 1407, 1358, 1297, 1255, 1190, 1122, 1108, 1091, 1012, 933, 872, 838, 822, 717, 697, 676, 650, 641, 614, 567, 501, 464, 452. **HRMS** (ESI-TOF), calculated for $C_{20}H_{21}N_3O_4$, *m/z:* 357.13 $[M+H]^+$; found 357.1408.

3-(4-nitrophenylamino)-(tricyclo[3.3.1.1(3,7)]decyl-1amino)-cyclobut-3-ene-1,2-dione (4):

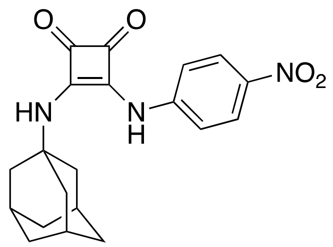

S5 (175 mg, 0.665 mmol, 1 eq), and $Zn(OTf)_2$ (49 mg, 0.133 mmol, 0.2 eq),were dissolved in EtOH (15 mL). This mixture was brought to reflux. To this, was added – dropwise, a solution of 1-adamantylamine (110 mg, 0.73 mmol, 1.1 eq), dissolved in EtOH (5 mL), over the course of five minutes. Reaction was stirred at reflux for 5hrs & subsequently room temperature, overnight. The observed precipitate was isolated by vacuum filtration to resolve the crude material. Crude product was washed with 3 x 2 ml portions of EtOH, to afford the title compound as a brick red solid in a 38% yield. **^{1}H NMR** (500 MHz, DMSO) δ

10.08 (s, 1H), 8.22 (br d, 2H), 8.03 (s, 1H), 7.66 (br d, *J* = 6.7 Hz, 2H), 2.12 (br s, 3H), 1.99 (br s, 6H), 1.66 (br s, 6H). **^{13}C NMR** (126 MHz, DMSO) δ 183.9, 180.2, 170.6, 163.7, 145.7, 141.9, 126.1, 118.2, 53.6, 42.8, 35.6, 29.4. **IR** (ATR): ν_{max} (cm^{-1}) = 3302, 2907, 1802, 1715, 1625, 1593, 1531, 1507, 1493, 1414, 1381, 1331, 1310, 1283, 1187, 1113, 1056, 994, 885, 842, 793, 750, 678, 640, 607, 502, 460. **HRMS** (ESI-TOF), calculated for $C_{20}H_{21}N_3O_4$, *m/z:* 368.16 [M+H]$^+$; found 368.1608, also found [M+Na]+; 390.1426.

3-(2-chlorophenylamino)-4-(tricyclo[3.3.1.1(3,7)]decyl-1-amino)-cyclobut-3-ene-1,2-dione (5):

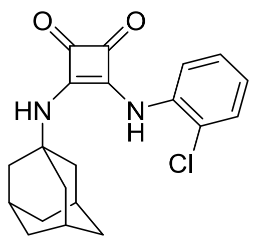

S6 (76 mg, 0.31 mmol, 1 eq), and Zn(OTf)$_2$ (22 mg, 0.062 mmol, 0.2 eq) were dissolved in EtOH (10 mL). Whilst stirring, a solution of 1-adamantylamine (51 mg, 0.34 mmol, 1.1 eq) in EtOH (2 mL) was added dropwise. The resultant reaction mixture was allowed to stir for 48 hrs. Subsequently, the formed precipitate was isolated via vacuum filtration, & washed with 3 x 2 ml portions of cold ethanol to afford the desired product as a yellow solid in a 21% yield. **^{1}H NMR** (500 MHz, DMSO-*d$_6$*) δ 9.23 (br, NH, 1H), 8.3 (br, NH, 1H), 7.45 (dd, J = 8.25, ArH, 1H), 7.43 (dd, J = 8.1, ArH, 1H), 7.26 (dt, J = 7.3, ArH, 1H), 7.05 (dt, J = 7.3, ArH, 1H), 2.05 (br s, CH, 3H), 1.94 (br s, CH_2, 6H), 1.59 (br s, CH_2, 6H). **^{13}C NMR** (126 MHz, DMSO) δ 182.5, 179.5, 169.0, 164.1, 138.9, 129.0, 122.5, 117.6, 52.7, 42.5, 35.0, 28.8. **IR** (ATR): ν_{max} (cm^{-1}) = 3220, 3154, 3029, 2902, 2852, 1786, 1682, 1597, 1564, 1532, 1518, 1438, 1359, 1300, 1191, 1120, 1110, 1094, 1063, 1040, 923, 825, 811, 746, 706, 656, 641, 613, 582, 568, 535, 459, 444. **HRMS** (ESI-TOF), calculated for $C_{20}H_{21}ClN_2O_2$, *m/z:* 356.13 [M+H]$^+$; found 357.1685, also found [M+Na]$^+$; 379.1186.

3-phenylamino-4-(tricyclo[3.3.1.1(3,7)]decyl-1-amino)-cyclobut-3-ene-1,2-dione (6):

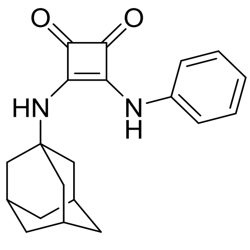

S7 (100 mg, 0.36 mmol, 1 eq), and $Zn(OTf)_2$ (26 mg, 0.072 mmol, 0.2 eq), were dissolved in EtOH (10 mL). To this was added, adamantylamine (53 mg, 0.4 mmol, 1.1 eq). The resultant mixture was allowed to stir overnight, whereupon the observed precipitate was isolated via vacuum filtration, followed by 3 x 2 ml washings of EtOH to afford the desired product as a white solid in a 67% yield. **^{1}H NMR** (500 MHz, DMSO) δ 9.66 (s, 1H), 7.87 (s, 1H), 7.48 (dd, *J* = 8.6, 1.0 Hz, 2H), 7.40 – 7.33 (td, *J* = 7.5, 2H), 7.08 – 6.98 (t, *J* = 7.6, 1H), 2.11 (s, 3H), 1.99 (d, *J* = 2.5 Hz, 6H), 1.66 (s, 6H). **^{13}C NMR** (126 MHz, DMSO) δ 182.7, 180.3, 169.5, 164.8, 139.4, 129.8, 123.1, 118.4, 53.2, 42.9, 35.7, 29.4. **IR** (ATR): ν_{max} (cm^{-1}) = 3231, 3031, 2906, 2847, 1789, 1671, 1620, 1603, 1573, 1532, 1500, 1461, 1358, 1304, 1297, 1257, 1153, 1123, 1094, 1063, 1020, 894, 808, 755, 712, 689, 656, 641, 612, 598, 559, 501, 463. **HRMS** (ESI-TOF), calculated for $C_{20}H_{22}N_2O_2$, *m/z:* 322.17 $[M+H]^+$, found 323.1754; also found $[M+Na]^+$, 345.1577.

$[Ru(Phen)_2(Phendione)]Cl_2$:

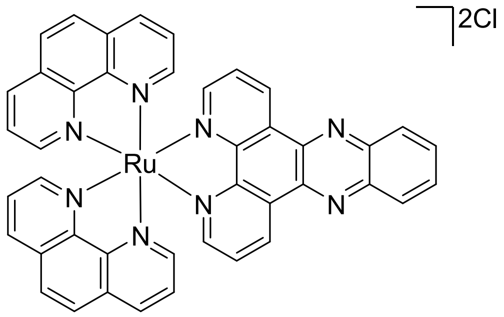

1,10-Phenanthroline-5,6-dione (105 mg, 0.5 mmol, 1 eq), 1,2-phenylene diamine (54 mg, 0.5 mmol, 1 eq) and a catalytic quantity of *p*-toluenesulfonic acid were dissolved in EtOH (10 mL), and stirred at reflux for 12 hr. The mixture was concentrated *in-vacuo*, recrystalised from minimal EtOH, and subsequently washed with cold EtOH (2 mL), and Et_2O (5 ml) before being dried using Schlenk technique to afford the desired dipyrido[3,2-a:2',3'-c]phenazine ligand intermediate as a beige solid, which was used immediately in the next step.

cis-Ru(Phen)$_2$Cl$_2$ (140 mg, 0.255 mmol, 1 eq from isolated yield of dipyrido[3,2-a:2',3'-c]phenazine), and dipyrido[3,2-a:2',3'-c]phenazine (81 mg, 0.3 mmol, 1.2 eq) were added to a microwave tube, dissolved in a 50:50 H_2O/EtOH mixture (5 mL), and sparged with N_2 for 10 mins. This tube was sealed, and subjected to microwave irradiation at 140 ^{o}C for 40 mins, whereafter the mixture was filtered through Agilent 0.45 μm PES filter membranes. To the filtrate was added a saturated solution of ethanolic NH_4PF_6 (2 mL), to precipitate the desired PF_6 salt of the complex. The suspension was centrifuged at 4000 RPM for 5 min to pellet the desired precipitate, which was subsequently washed with H_2O, EtOH, and Et_2O (5 mL) before being dried under a gentle stream of N_2. The precipitate was dissolved in a minimal amount of acetonitrile and transferred to a 50 ml RBF, which was then sealed in a vessel under atmospheric pressure containing Et_2O, and placed in the dark for 24 hr to facilitate the generation of crystals. After 24 hr the RBF was removed from the vessel, the mother liquor was decanted, and the crystals formed were washed with a minimal amount of Et_2O to afford the desired complex as its PF_6 form. To generate the Cl^- salt, the crystals were dissolved in MeOH (15 ml), and stirred over Amberlyst anion exchange resin (Cl form) for 1 hr, before being filtered and concentrated *in vacuo* to afford the title compound as a bright red crystalline solid in a 28 % yield. All spectral data is in good agreement with literature.[6] **^{1}H NMR** (500 MHz, DMSO) δ 9.62 (dd, *J* = 8.2, 1.3 Hz, 2H), 8.85 – 8.76 (m, 4H), 8.54 (dd, *J* = 6.6, 3.4 Hz, 2H), 8.41 (s, 4H), 8.28 (dd, *J* = 5.3, 1.2 Hz, 2H), 8.25 – 8.17 (m, 4H), 8.07 (dd, *J* = 5.2, 1.2 Hz, 2H), 7.91 (dd, *J* = 8.2, 5.4 Hz, 2H), 7.80 (ddd, *J* = 21.2, 8.3, 5.3 Hz, 4H). **^{13}C NMR** (126 MHz, DMSO) δ 154.4, 151.2, 148.9, 147.5, 140.7, 137.4, 133.1, 130.9, 129.9, 128.5, 117.7.

4-bromo-*N*-ethyl-1,8-naphthalimide (N1):

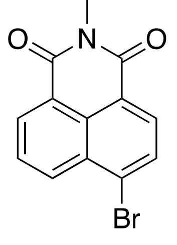

4-bromo-*N*-ethyl-1,8-naphthalimide was synthesised from 4-bromo naphthalic anhydride, ethylamine HCl, and triethylamine, according to literature. All spectral data is in good agreement with that reported.[7]

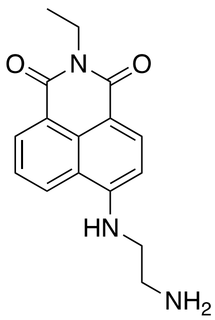

4-(2-aminoethyl)amino-*N*-ethyl-1,8-naphthalimide (N2):

4-(2-aminoethyl)amino-*N*-ethyl-1,8-naphthalimide was synthesised from **N1**, and ethylene diamine, according to literature. All spectral data is in good agreement with that reported.[7]

3-adamantylamino-4-(4-(2-aminoethyl)amino-*N*-ethyl-1,8-naphthalimido)-cyclobut-3-ene-1,2-dione (7):

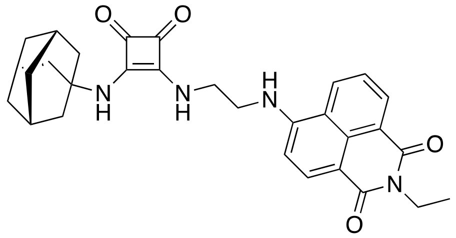

To a stirring solution of **5.9** (69 mg, 0.25 mmol, 1eq) in EtOH (5 mL), was added triethylamine (50 mg, 0.5 mmol, 2 eq), and **5.24** (71 mg, 0.25 mmol, 1eq). The resultant mixture was brought to reflux for 18 hr, whereafter the solvent was removed *in-vacuo* to afford a yellow crude mixture, which was triturated with Et_2O (3 x 10 mL), and washed with cold EtOH (5 mL), before being recrystallised from EtOAc to afford the title compound as a bright yellow solid in a 68% yield. **^{1}H NMR** (600 MHz, DMSO) δ 8.66 (d, *J* = 8.1 Hz, 1H), 8.44 (d, *J* = 7.2 Hz, 1H), 8.28 – 8.23 (m, 1H), 7.88 (s, 1H), 7.71 – 7.62 (m, 2H), 7.50 (s, 1H), 6.92 (d, *J* = 8.2 Hz, 1H), 4.05 (dd, *J* = 14.1, 7.0 Hz, 2H), 3.86 (s, 2H), 3.63 (d, *J* = 5.3 Hz, 2H), 2.06 (2s (rotameric) 3H), 1.79 (2s (rotameric, 6H), 1.69 – 1.55 (br q, 6H), 1.18 (t, *J* = 7.0 Hz, 3H). **^{13}C NMR** (151 MHz, DMSO) δ 182.3, 180.5, 168.7, 168.0, 163.5, 162.6, 150.7, 133.9, 130.7, 129.3, 128.4, 124.4, 121.9, 120.1, 108.3, 104.0, 51.9, 43.8, 42.4, 42.0,

35.3, 34.8, 33.9, 29.0, 28.1, 13.3. **HRMS** (ESI-TOF), calculated for $C_{30}H_{32}N_4O_4$, *m/z:* 512.24, found $[M+H]^+$; 513.2493, also found [M+Na]+; 535.2311.

3-(4-(2-aminoethyl)amino-*N*-ethyl-1,8-naphthalimido)-4-(3,5-bis(trifluoromethyl)phenyl amino)-cyclobut-3-ene-1,2-dione (8):

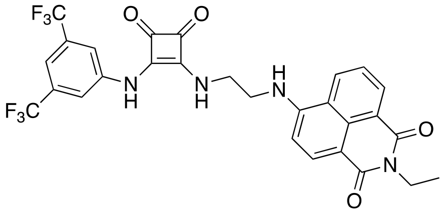

3-(4-(2-aminoethyl)amino-*N*-ethyl-1,8-naphthalimido)4-(3,5-bis(trifluoromethyl)phenylamino)-cyclobut-3-ene-1,2-dione was synthesised from **S2**, **N2**, and triethylamine, according to literature. All spectral data is in good agreement with that reported.[7]

Experimental procedures

Anion binding studies: All tetrabutylammonium halide salts (TBAX) and the receptors were lyophilised before use, and halide salts were stored under vacuum in a dessicator. Solutions of the TBA salts were made up in DMSO-*d6*, which was dried over 3Å molecular sieves before use, to a concentration of 300 mM. An aliquot of stock solution of receptor in DMSO-*d6* was diluted to 1 mL (2.5 mM). 600 µL of this solution was added to an NMR and the ^{1}H NMR spectrum was recorded. Subsequent additions of aliquots of TBAX solutions were added to the NMR tube and shaken vigorously to ensure homogenisation. This process was repeated up to 22 equivalents of halide was reached.

The ^{1}H NMR spectra were analysed and processed, and stackplots were generated using MestReNova 6.0.2 software. A global fitting analysis assuming a 1:1 binding model was employed to provide the binding constant (Ka/M^{-1}), by fitting of the chemical shift changes of the NH signals as function of anion concentration using the open access BindFit software program.

Lucigenin Cl^-/NO_3^- anion exchange assay

LUV preparation and Lucigenin assays were carried out following procedures outlined by Valkenier and co-workers.[8] Large unilamellar liposomes (LUVs) were synthesized using 1-palmitoyl-2-oleoyl-sn-glycero-3-phosphocholine (POPC)(11.2 µmol, soln. in $CHCl_3$) and Cholesterol (4.8 µmol) mixed in a ratio of 7:3. This mixture was brought to dryness under gentle vacuum and subsequently, the mixture was rehydrated in an aqueous Lucigenin solution (1 mL, 0.8mM lucigenin in 225 mM $NaNO_3$) and stirred for 1 h to obtain heterogeneous vesicles. After that, the vesicles underwent 9 freeze-thaw cycles and were extruded through a 0.2 mm polycarbonate membrane 29 times to obtain monodisperse LUVs. A Sephadex G-100 size-exclusion column was employed to remove any unencapsulated lucigenin. The collected liposomes were diluted to 40 mL with 225 mM $NaNO_3$ to obtain a homogeneous 0.4 mM liposome solution. Lucigenin fluorescence quenching was monitored in a time dependent manner using an Agilent Spectrofluorimeter to access Cl^- influx (λ_{ex} = 430 nm, λ_{em} = 505 nm). Cl^- transport behaviour was initiated at t=0 s by adding each compound as a solvate in DMSO followed by a 25 mM NaCl pulse (75µL, 1 M NaCl in 225 nm $NaNO_3$) to a 3 mL liposome solution. The data was collected for at least 600 s and then Triton X-100 (5% w/w in water) was added to lyse the liposomes, and normalize to 100% efflux. The recorded data was normalised and the fraction fluorescence intensity at 270 s of each tested concentration of transporters (mol%) was plotted against transporter concentration (mol%). The data obtained was fitted to the Hill equation to obtain the EC_{50} value at 270 s (the concentration to obtain the half maximum effect) by using Originlab 2024b or Graphpad Prism 10:

$$y = START + (END - START) * x^n/(k^n + x^n)$$

Where n is the Hill coefficient, k presents the EC_{50} value.

***Staphylococcus aureus* culture conditions**

S. aureus (ATCC33951) was cultured at 37 ^{o}C in Nutrient broth (Oxoid), in an orbital shaker at 200 rpm. Stocks were kept on nutrient agar, stored at 4 ^{o}C for up to 2 months, or as glycerol stocks (50/50 glycerol:culture), stored at -70 ^{o}C indefinitely.

Methicillin-resistant *Staphylococcus aureus* culture conditions

Methicillin-resistant *S. aureus* (clinical isolate – St. James' University Hospital, Dublin) was cultured at 37 ^{o}C in Nutrient broth (Oxoid), containing 25 µg/mL Ampicillin in an orbital shaker at 200 rpm. Stocks were kept on nutrient agar containing 25 µg/mL Ampicillin, stored at 4 ^{o}C for up to 2 months, or as glycerol stocks (50/50 glycerol:culture), stored at -70 ^{o}C indefinitely.

***Pseudomonas aeruginosa* culture conditions**

Pseudomonas aeruginosa (PA01) was cultured at 37 ^{o}C in Nutrient broth (Oxoid), in an orbital shaker at 200 rpm. Stocks were kept on nutrient agar, stored at 4 ^{o}C for up to 2 months, or as glycerol stocks (50/50 glycerol:culture), stored at -70 ^{o}C indefinitely.

***Klebsiella pneumoniae* culture conditions**

Klebsiella pneumoniae (*bla-Vim1*) was cultured at 37 ^{o}C in Nutrient broth (Oxoid), containing 50 µg/mL Ampicillin in an orbital shaker at 200 rpm. Stocks were kept on nutrient agar containing 50 µg/mL Ampicillin, stored at 4 ^{o}C for up to 2 months, or as glycerol stocks (50/50 glycerol:culture), stored at -70 ^{o}C indefinitely.

Growth Inhibition Assays

Bacterial cultures were brought to early stationary phase overnight, in nutrient broth (containing 25/50 µg/mL Ampicillin for MRSA/*K. pneumoniae,* respectively) at 37 °C. Cultures were diluted to an OD600 = 0.01. Aliquots of Culture (100 µl) were added to 100 µL serially diluted compound (200 – 0.78 µM) in nutrient broth in a 96-well plate (Sarstedt, Germany). Plates were incubated at 37 °C for 24 h and growth was measured at 600 nm, where growth was measured and represented relative to control.

***Galleria mellonella* toxicity studies**

To ascertain inherent compound toxicity, 10 healthy *G. mellonella* larvae were selected and weighed ensuring to keep larval weight approx. 0.25 g (± 0.05 g). This constituted one sample set for experiments. Larvae were injected into the left hind pro-leg with 20 µL of a 50% DMSO/PBS stock of the respective compound to bring the haemolymph concentration to that which is required. An injection of sterile PBS served as a negative control. Larvae were subsequently monitored for up to 96 hours, for signs of toxicity, melanisation and death.

MQAE chloride influx assay

From an overnight culture brought to stationary phase (OD_{600} = ~1.0) was taken samples, brought to an OD_{600} = 0.2, which were treated with *N*-Ethoxycarbonylmethyl-6-methoxyquinolium Bromide (MQAE) (10 mM) for 1 h at 37 ^{0}C. Subsequently, cells were washed with & resuspended in PBS. 2 mL of samples were taken & transferred to a fluorescence cuvette, whereupon fluorescence was read (λ_{exc} = 350 nm (slit width = 5 nm) λ_{em} = 460 nm (slit width = 5 nm)) using a Cary Eclipse Fluorescence Spectrophotometer (Agilent Technologies). Subsequently samples were treated with the Compound of interest for 5 mins whereafter fluorescence was measured as previous, with intensity plotted relative to control,

or measured over the course of 2 hrs, starting at T_0 (upon addition of compound). Alternatively, 200 µL aliquots of cell suspension were added to a 96-well plate, to the desired quantity, and were either untreated (control), or treated with the compound of interest for 5 mins, whereafter fluorescence was read using a BMG Labtech Clariostar Plus platereader, and plotted relative to control.

MTT assay

From an overnight culture of *S. aureus* brought to stationary phase (OD_{600} = ~1.0), was taken samples which were diluted to an OD_{600} = ~0.2 in either Hank's buffered salt solution, Cl^- free HBSS buffer, or Na^+ free buffer. Samples were treated with the respective compound for 5 h at 37 ^{0}C. Subsequently, Cell densities for each of the samples were standardised to an OD_{600} = 0.15, in the relevant buffer system. To each sample was added Triphenyl Tetrazolium Chloride (TTC), to a final concentration of 0.5 mg/mL. 100 µL of each sample was placed into the wells of a 96-well place in 8 replicates. Samples were incubated at 37 ^{0}C for 18 h, and subsequently supernatant was removed from each well, taking care as to not disturb the Formazan precipitate. To each well was then added 100 µL DMSO, with repeated pipetting to solubilise precipitate. Samples were read using a microplate reader at 550 nm & plotted as percentage cell viability relative to control.

Fluorescence microscopy

1 mL aliquots of an overnight culture brought to early stationary phase (OD600 = ~1.0) were centrifuged at 13000 rpm for 5 min to pellet cells. Supernatant was discarded & cells were washed with 2 x 1 mL PBS. Subsequently, cells were resuspended in 1 mL PBS, and treated with a the respective fluorophore or compound of interest at the specified concentration or

range thereof. Samples were incubated in the dark at 37 ^{o}C for 30 mins. Cells were pelleted by centrifugation at 13000 rpm for 2 mins. The afforded pellets were washed with 1 mL PBS, & finally resuspended in 100 μL PBS (confocal microscopy) or Prolong Gold antifade mountant solution (STED), of which 10 μL was mounted atop microscope slides, followed by the placement of an appropriate coverslip for STED or confocal and sealed with nail varnish, ensuring to minimise bleeding of the varnish into the sample. Slides were stored in the absence of light until processed and imaged.

Confocal and STED images were acquired using the 3D STED Falcon (objective: Leica HC PL APO CS2 100X/1.40 oil immersion). White light laser was used to excite the fluorophores with a max power of 0.00347 mW used close to the focal plane. STED DL 775 nm with a max power of 24.7 mW (30%) was used. Images were processed using LASX, LASX Falcon (FLIM) software, and ImageJ.

Scanning speed of 200 Hz was used for all images. Fluorophores were excited at λ_{exc} (nm) (power 0.4–1.4%), Fluorophores were imaged using CLSM, FLIM, FLIM-STED (DL 30%), and TauSTED mode (τ-strength 80, denoise 100, time gate 0.5–6 ns) was used for the HyD detector to filter pixels using the phasor signature produced for each STED image.

Quantification of fluorescence intensity was carried out upon deconvoluted images, using ImageJ. Regions of interest within images were identified based upon morphological considerations, and clearly identifiable markers, i.e. cytosolic and membrane regions. In certain instances, prior to quantification, brightfield microscopy was used to determine the specific location of desired ROI. All quantification, statistical analysis and graphics generation in this case was carried out using Graphpad Prism 1.8.0.1.

Propidium iodide assay

1 mL aliquots of *S. aureus* cultures brought to early stationary phase overnight were brought to an OD600 = 0.2 and 100 µL of this suspension was added to 96 well plates, whereafter 100 µL of PBS containing; no compound (- control), or compound (2x desired concentration) was added to the suspension. The resultant mixture was incubated at 37 ^{o}C for 2 hr whereafter fluorescence intensity from PI (DNA bound form) was quanitified ($\lambda_{exc/em}$ = 535/615 nm) using a Clariostar plate reader. In the case of time course experiments, Measurement cycles were set for two hours, following addition of the respective compound (T_0). Fluorescence was subsequently plotted as F/F_0.

DNA release luminescence assay

1 mL aliquots of *S. aureus* cultures brought to early stationary phase were standardised to an OD600 = 0.5, and treated with the respective compound at 37 ^{o}C for 1 hr in the absence of light, with agitation at 200 rpm. Subsequently, cells were centrifuged at 15000 rpm for 10 mins, and the supernatant was isolated, and placed on ice. To the supernatant was added $Ru(dppz)(phen)_2Cl_2$ (10 µM) and samples were incubated for 15 mins in the absence of light. Following incubation, luminescence intensity from the ^{3}MLCT band of the complex was quantified (λ_{em} = 620 nm), and plotted relative to untreated and cell-free media.

Statistical analysis of biological data: All biological experiments were carried out in triplicate, making use of cells of differing origins, on three independent occasions. With experiments concerning the use of 96-well plates, all experiments were carried out as three biological replicates of eight technical replicates. All data is presented as the mean $\pm$ SEM of each replicate, unless specified otherwise. All statistical analysis and significance determination was carried out using the Graphpad Prism 8.0.1 software package.

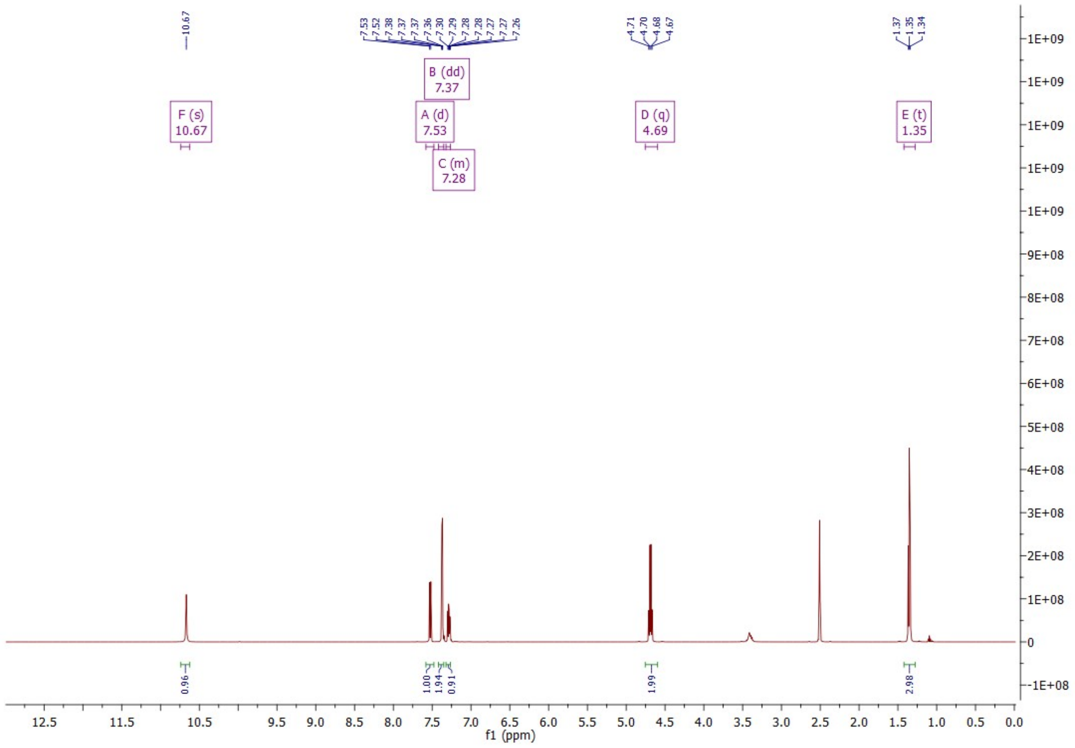

Figure S1: ^{1}H NMR spectrum of **S6** in DMSO-d_6.

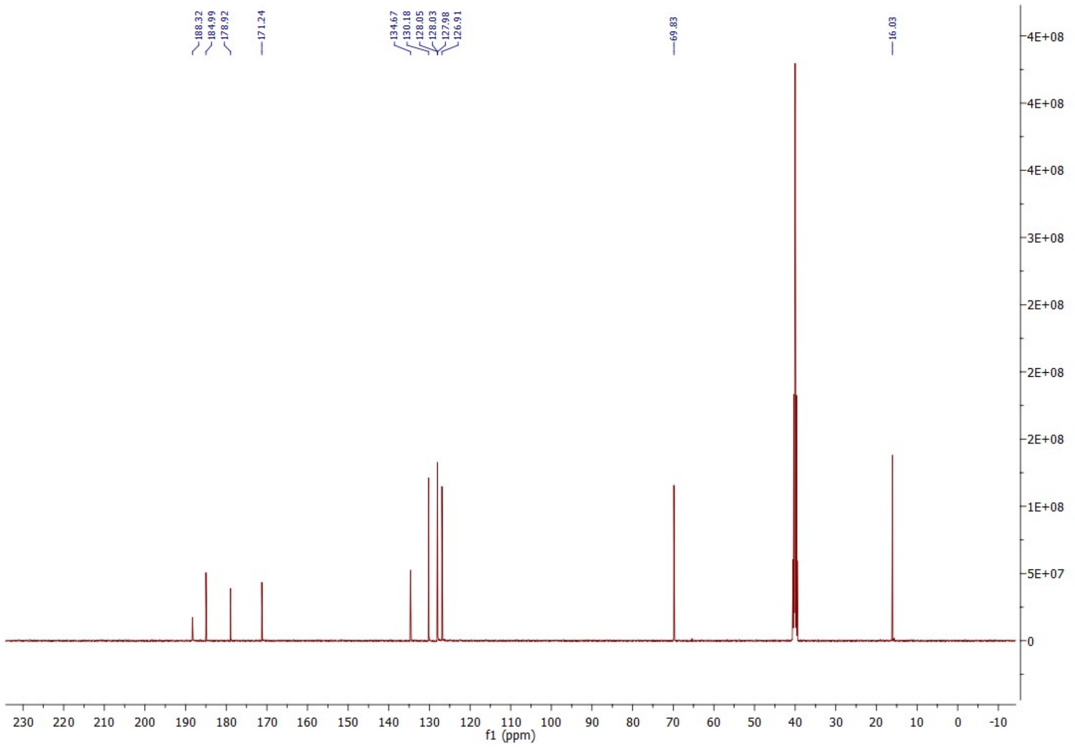

Figure S2: ^{13}C NMR spectrum of **S6** in DMSO-d_6.

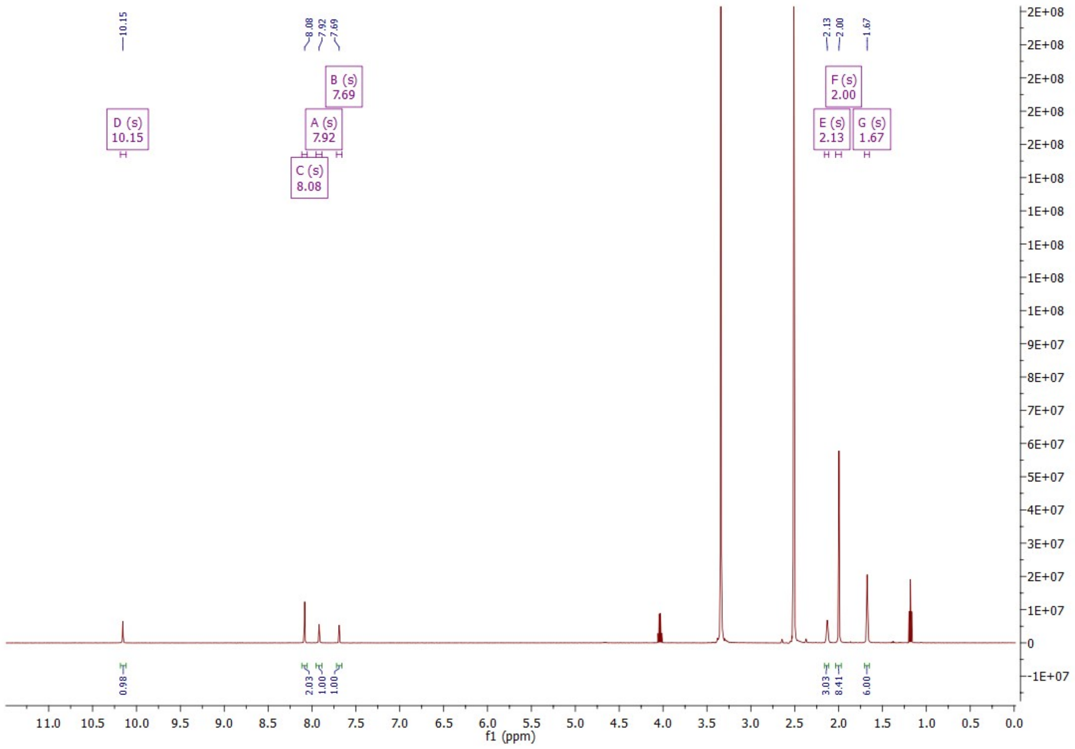

Figure S3: ^{1}H NMR spectrum of **1** in DMSO-d_6.

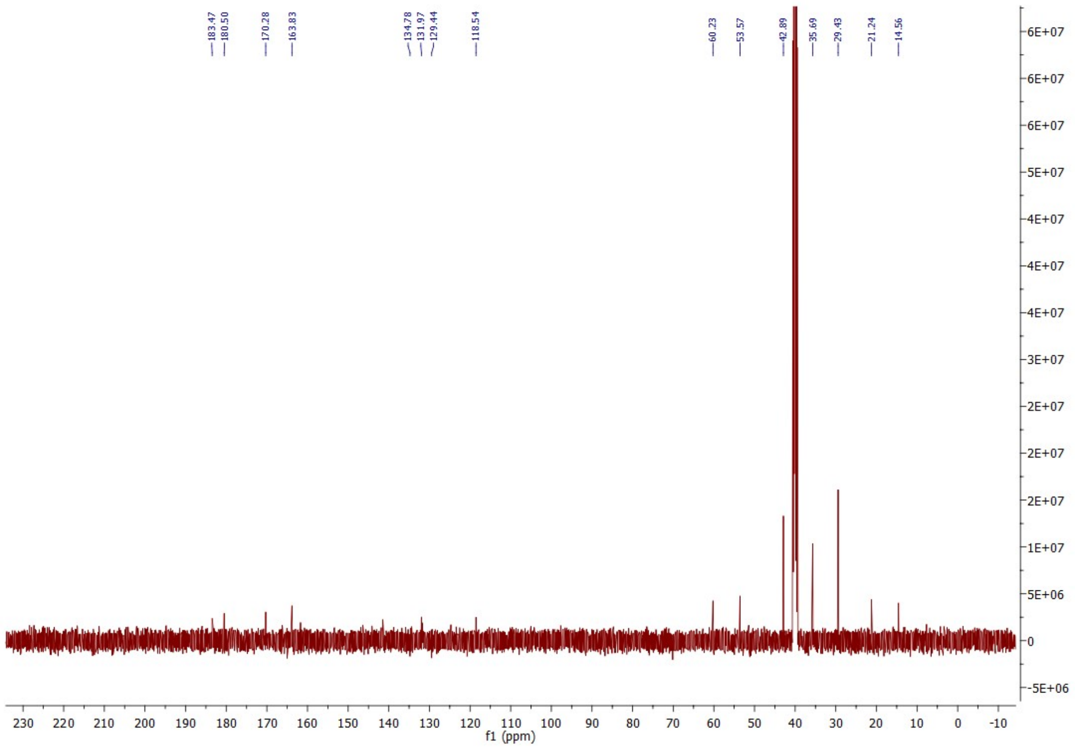

Figure S4: ^{13}C NMR spectrum of **1** in DMSO-d_6.

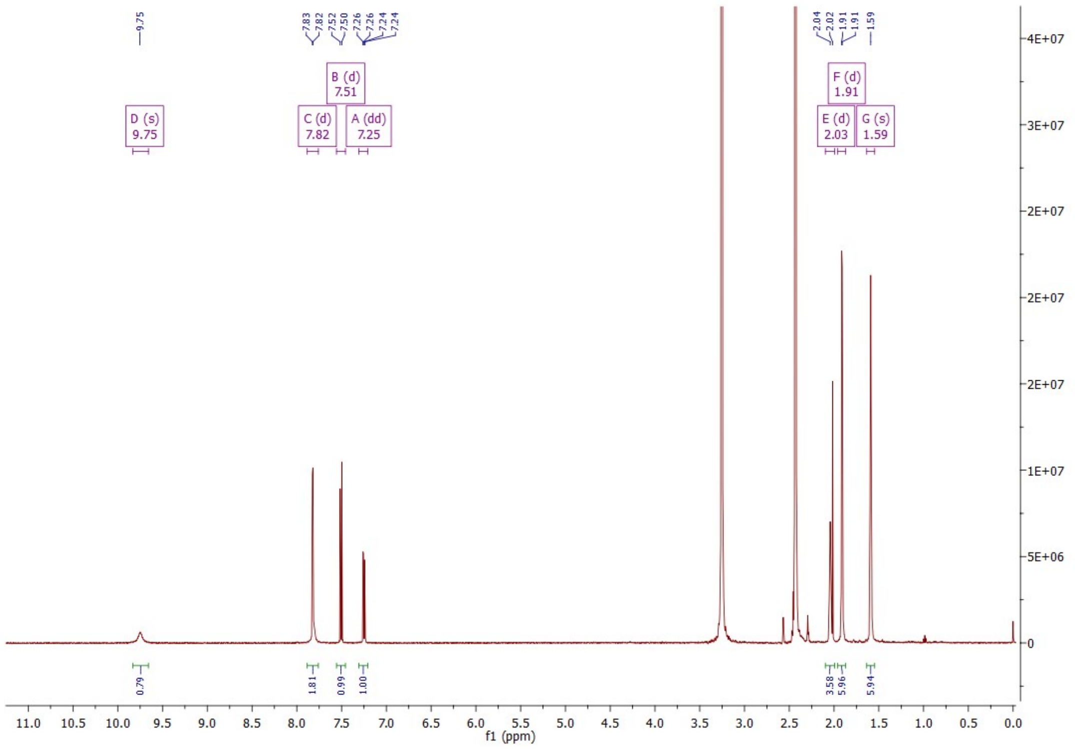

Figure S5: ^{1}H NMR spectrum of **2** in DMSO-d_6.

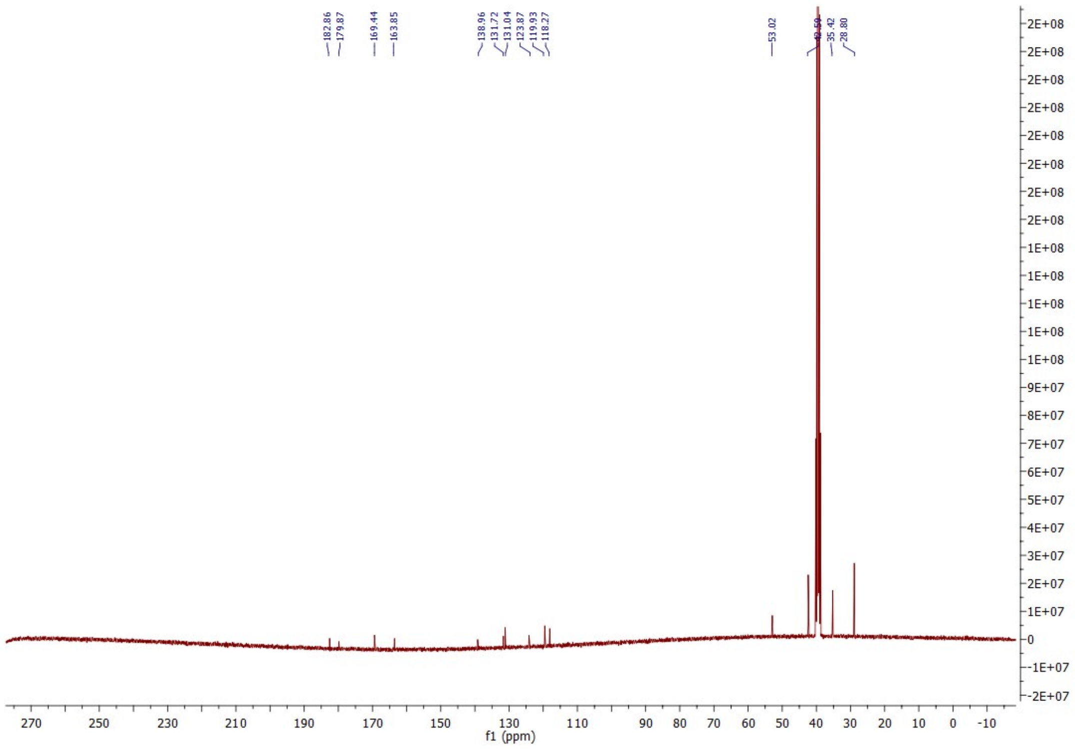

Figure S6: ^{13}C NMR spectrum of **2** in DMSO-d_6.

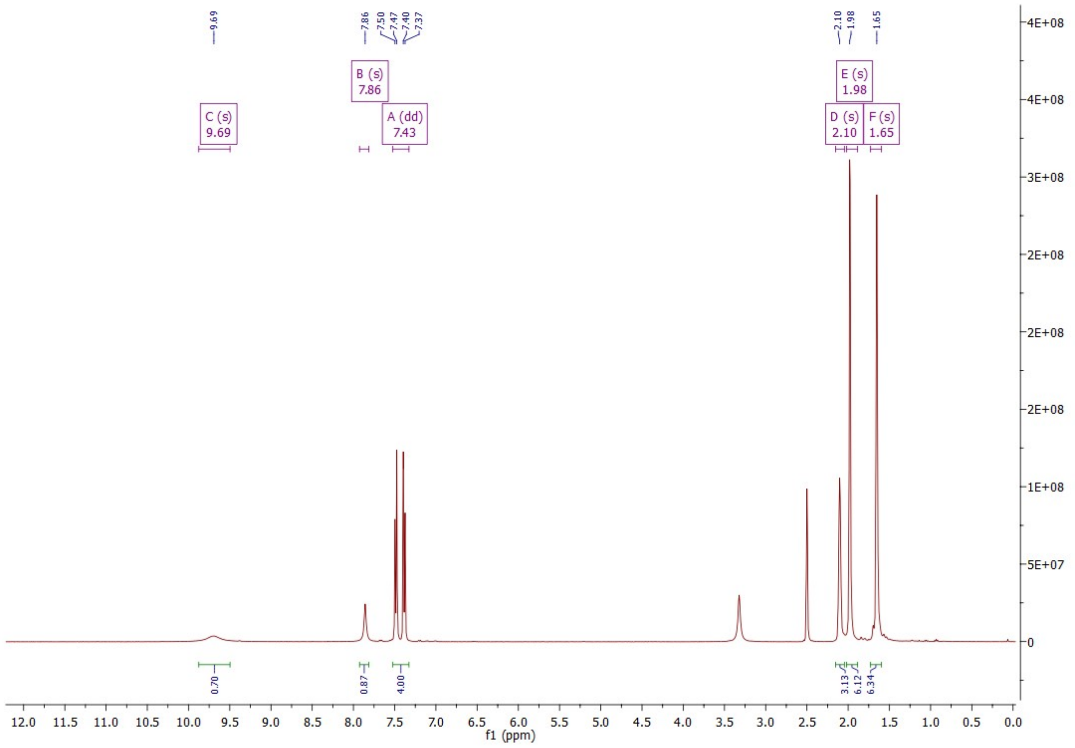

Figure S7: ^{1}H NMR spectrum of **3** in DMSO-d_6.

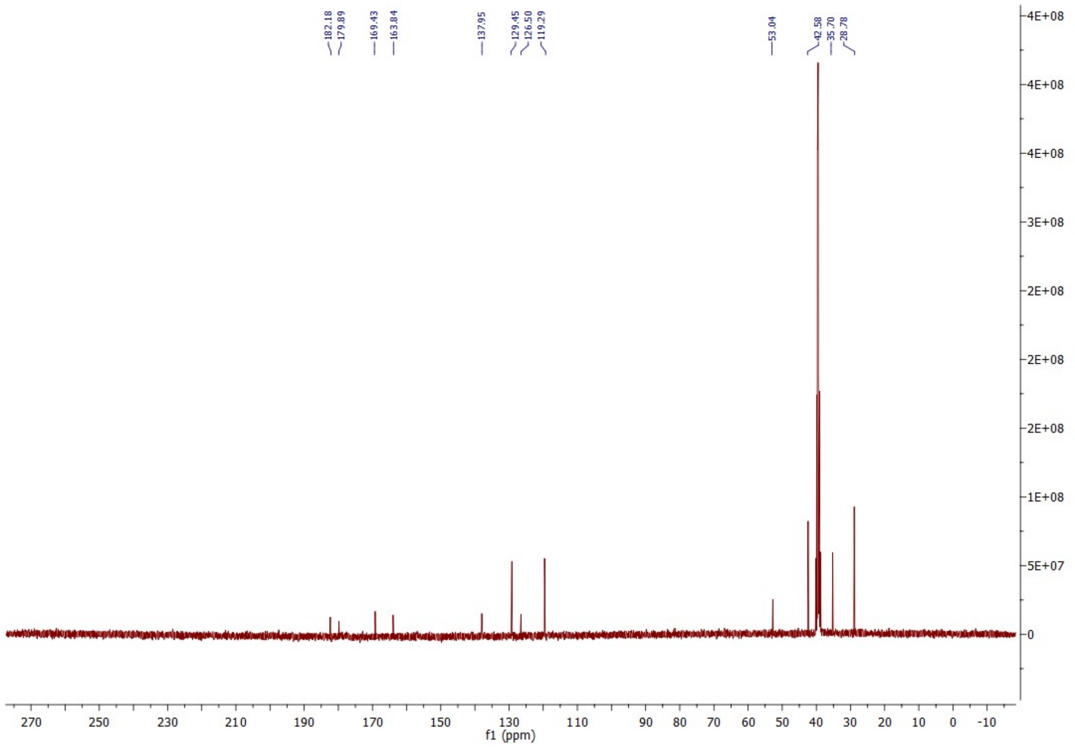

Figure S8: ^{13}C NMR spectrum of **3** in DMSO-d_6.

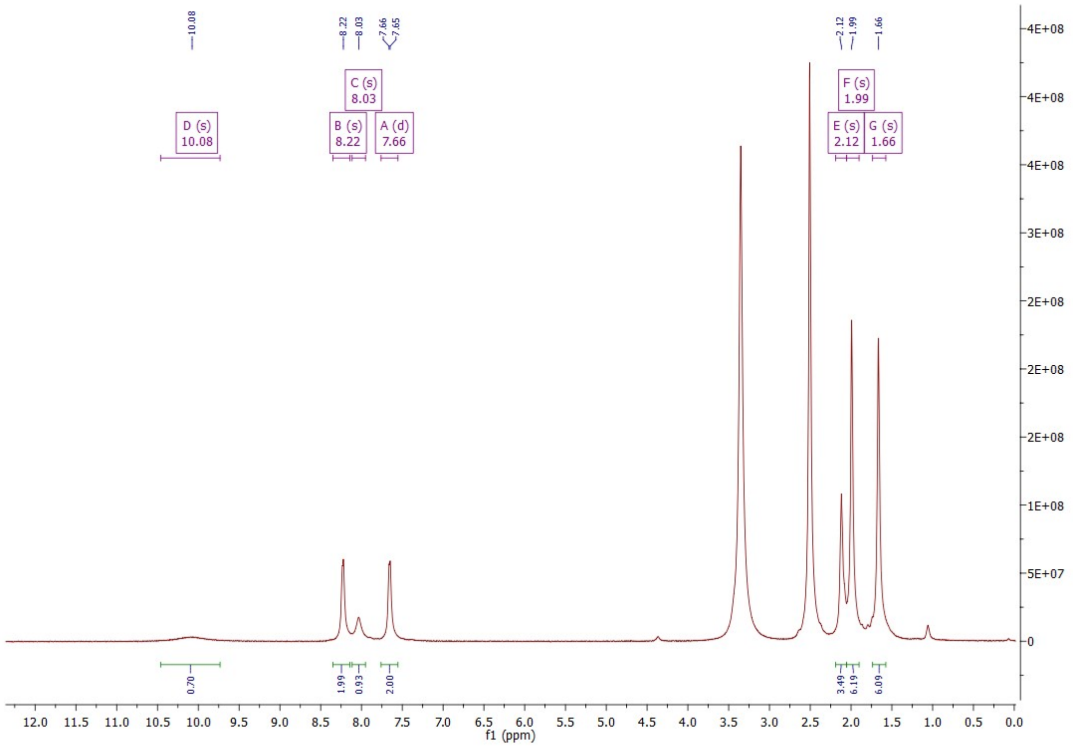

Figure S9: ^{1}H NMR spectrum of **4** in DMSO-d_6.

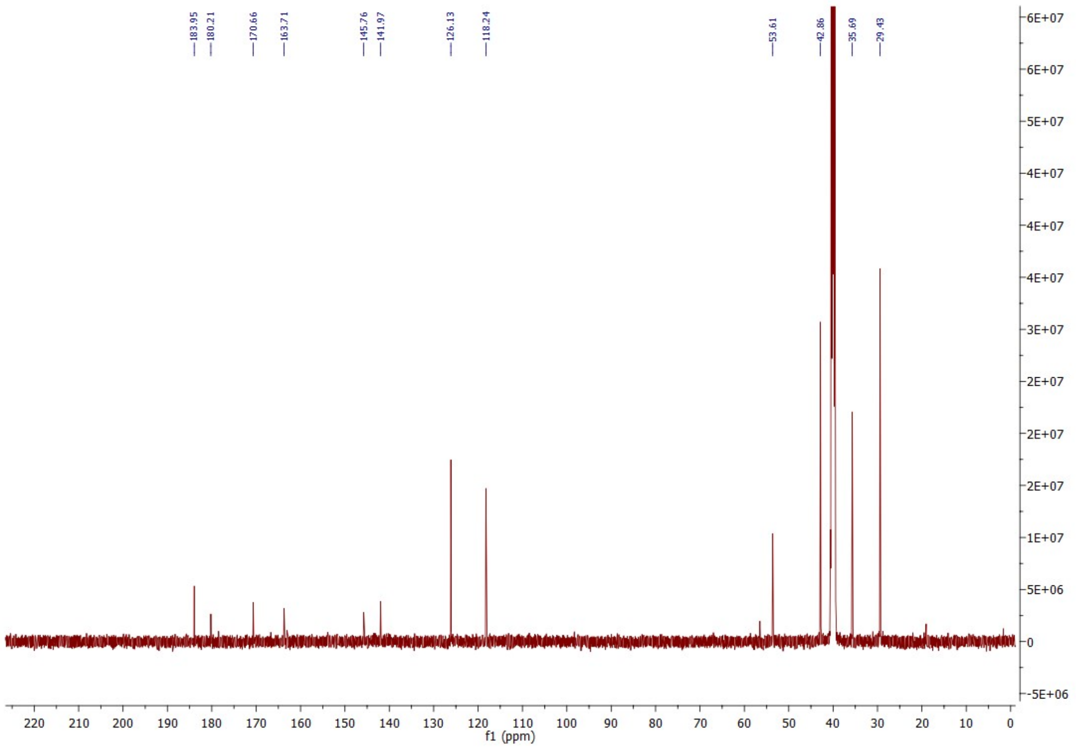

Figure S10: ^{13}C NMR spectrum of **4** in DMSO-d_6.

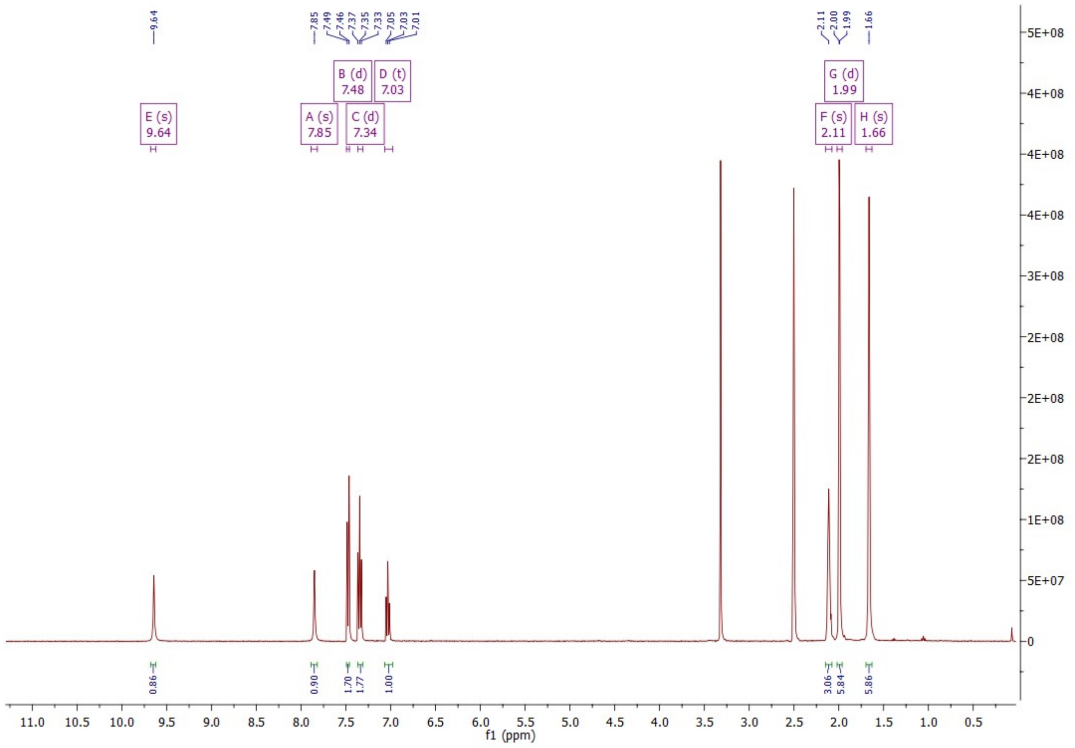

Figure S11: ^{1}H NMR spectrum of **5** in DMSO-d_6.

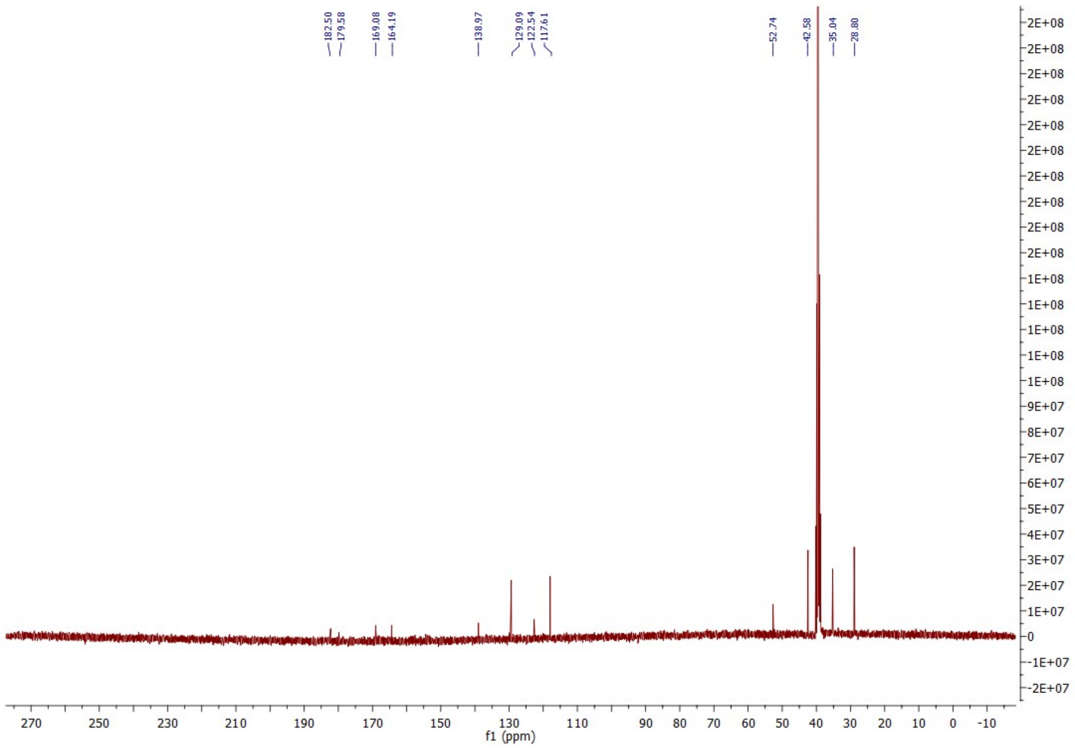

Figure S12: ^{13}C NMR spectrum of **5** in DMSO-d_6.

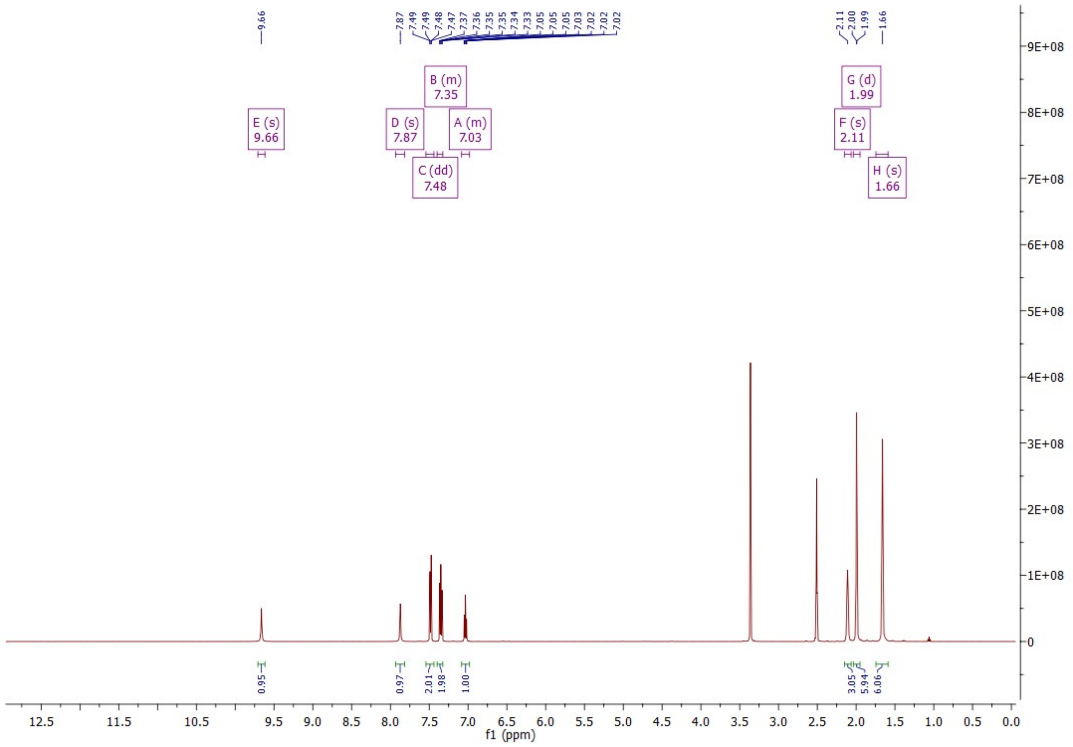

Figure S13: ^{1}H NMR spectrum of **6** in DMSO-d$_6$.

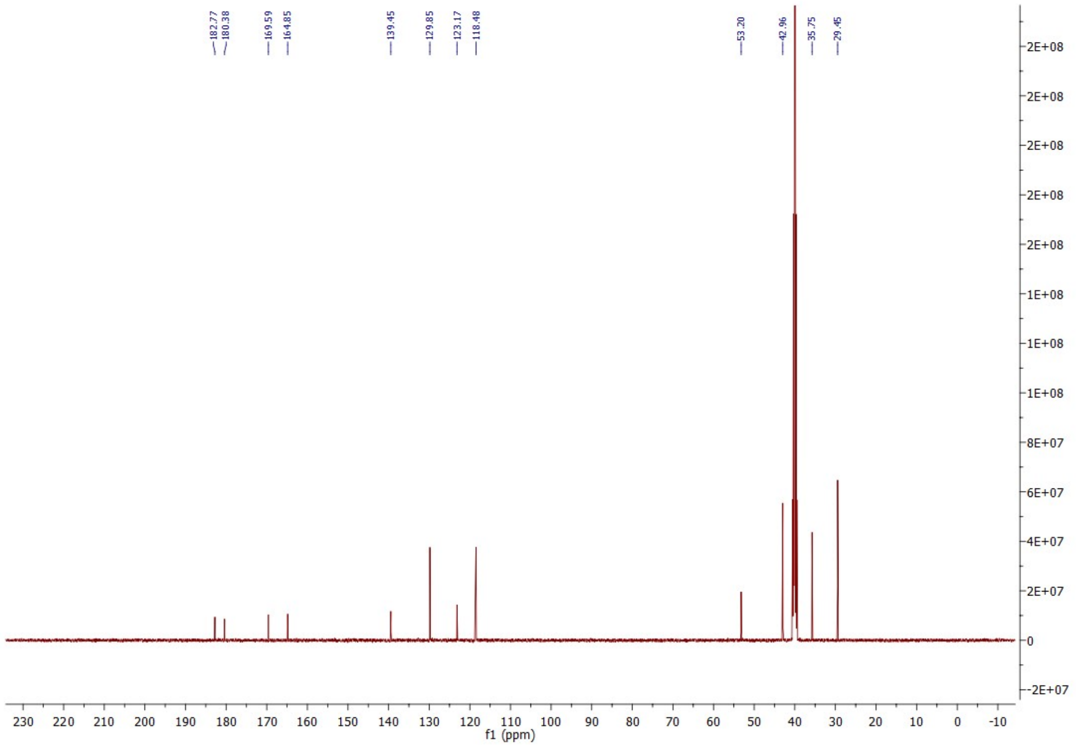

Figure S14: ^{13}C NMR spectrum of **6** in DMSO-d$_6$.

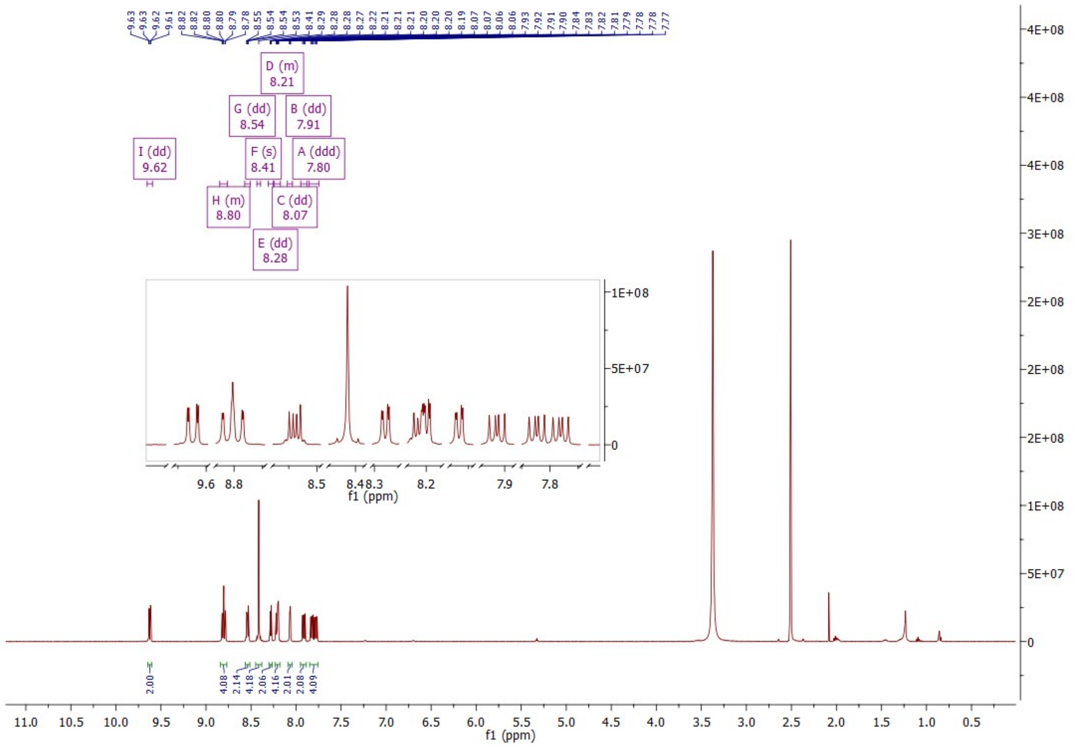

Figure S15: ^{1}H NMR spectrum of **$Ru(phen)_2(dppz)Cl_2$** in DMSO-d_6.

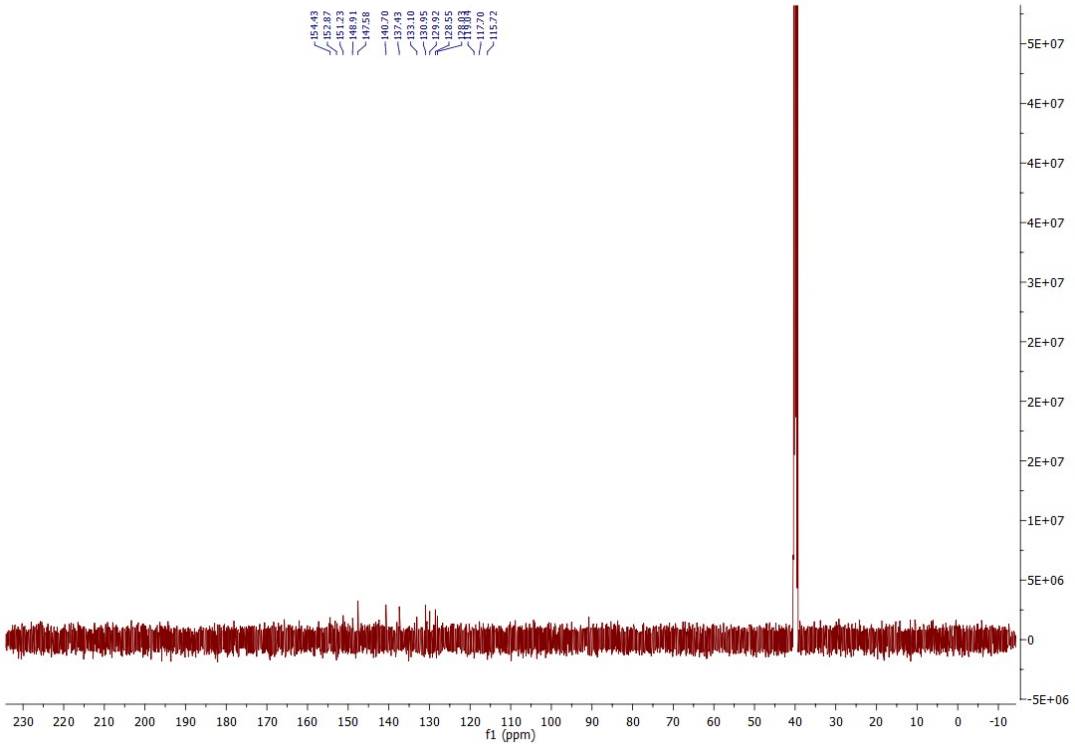

Figure S16: ^{13}C NMR spectrum of **$Ru(phen)_2(dppz)Cl_2$** in DMSO-d_6.

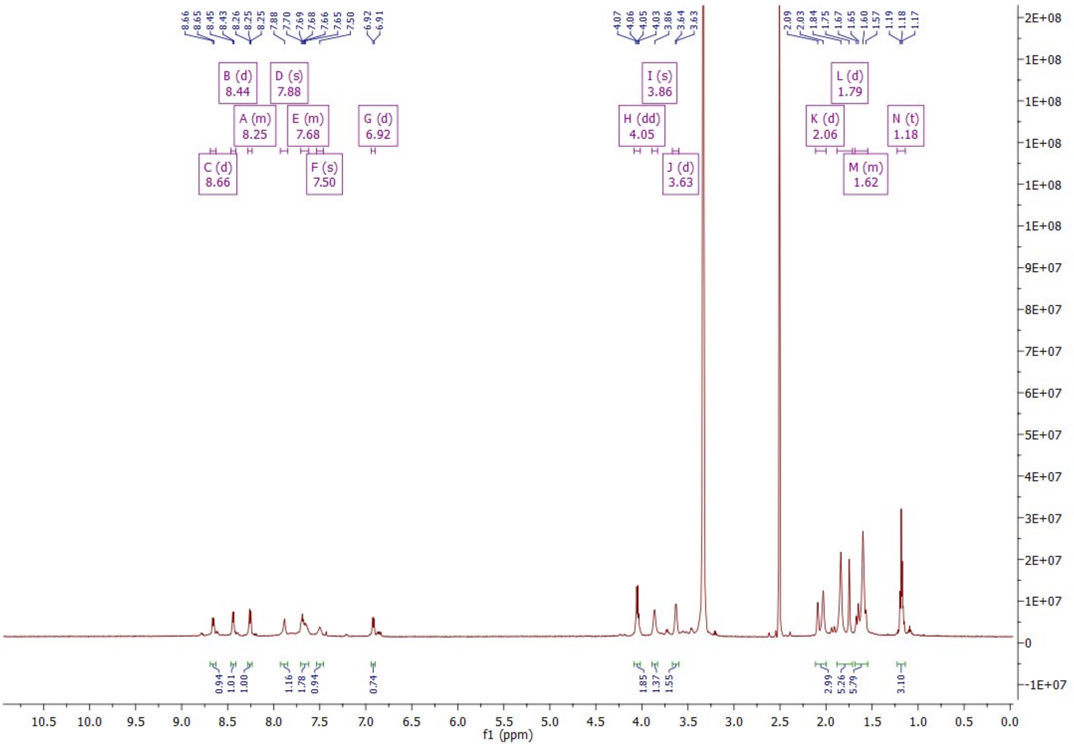

Figure S17: ^{1}H NMR spectrum of **7** in DMSO-d_6.

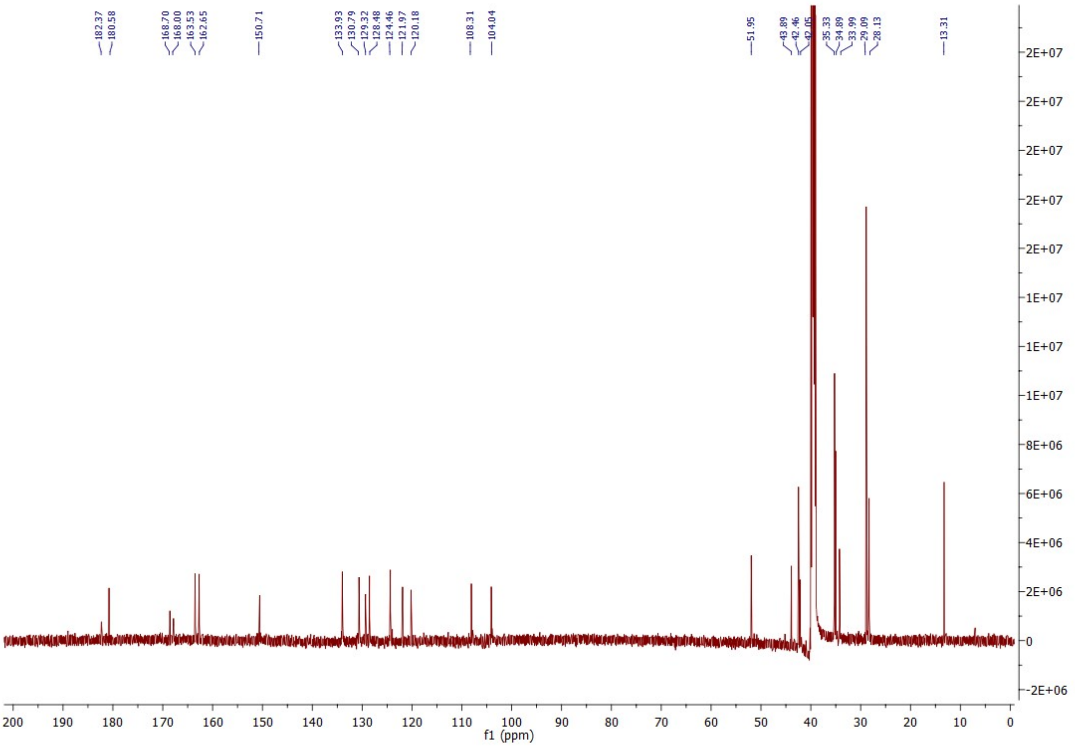

Figure S18: ^{13}C NMR spectrum of **7** in DMSO-d_6.

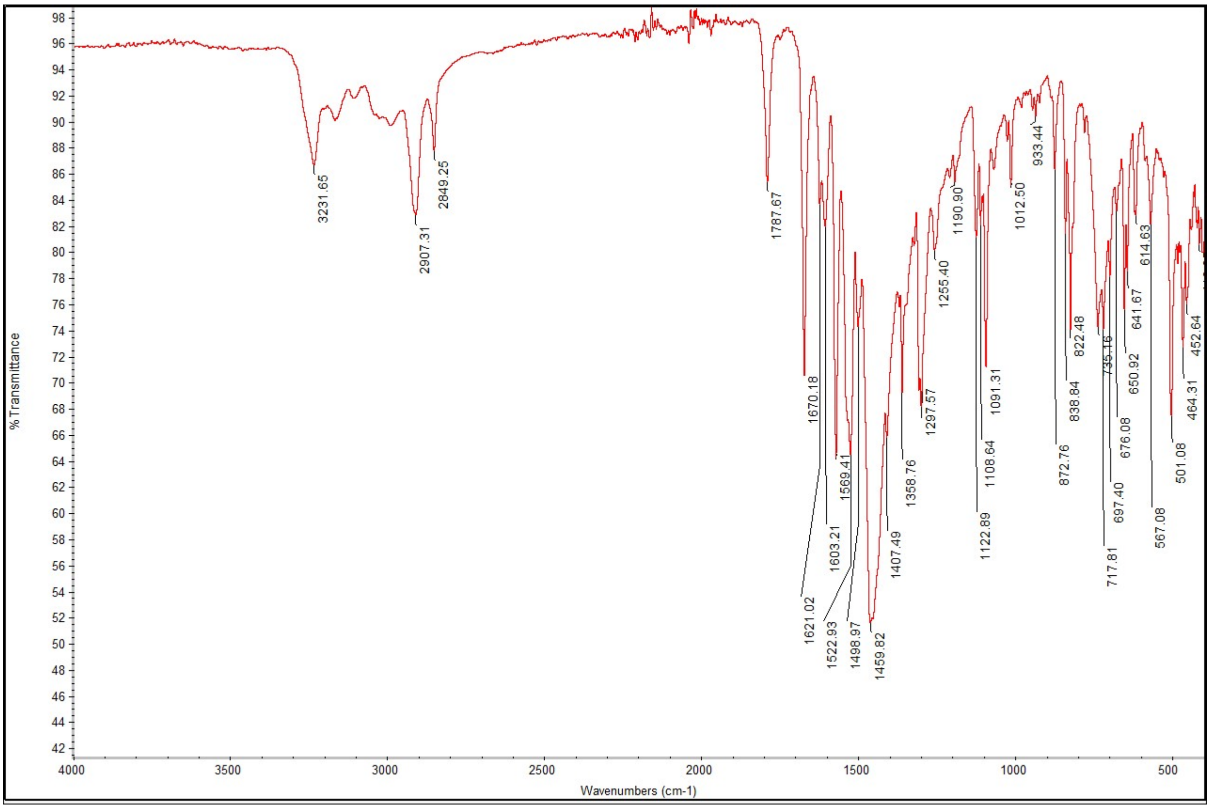

Figure S19: IR spectrum of **3**

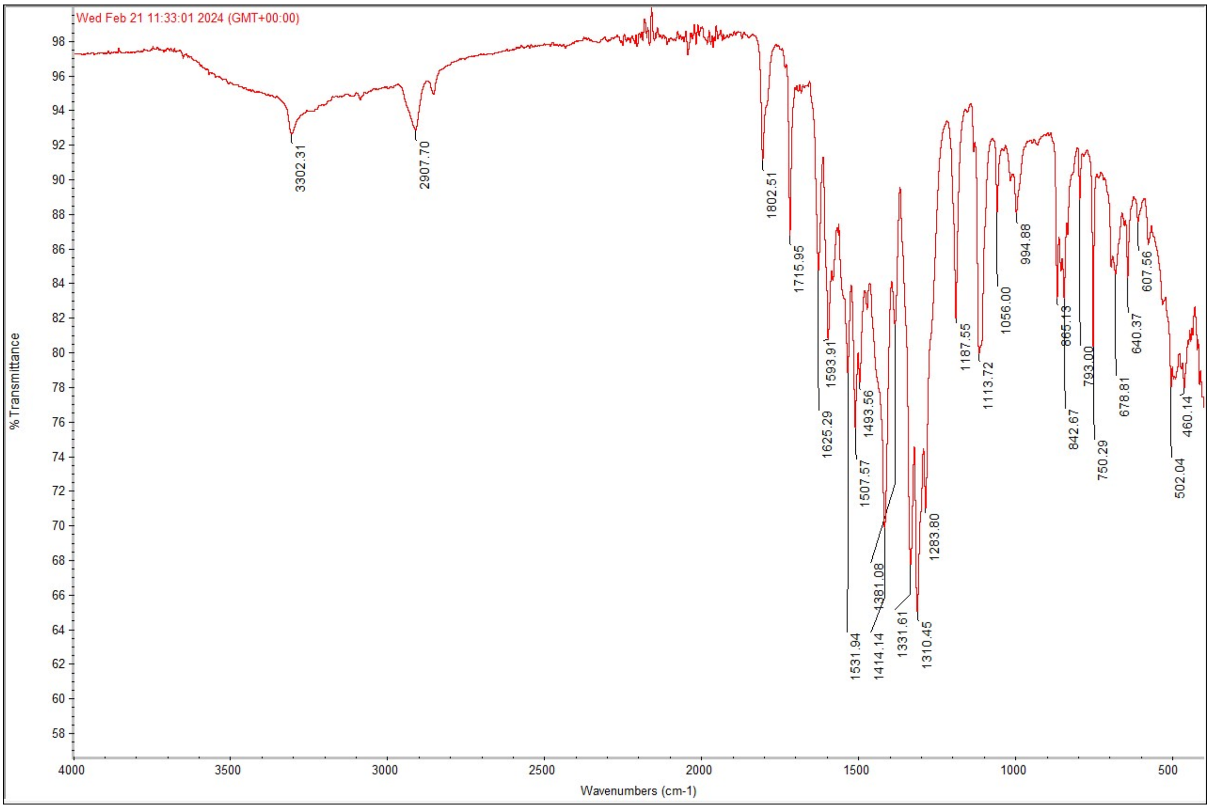

Figure S20: IR spectrum of **4**

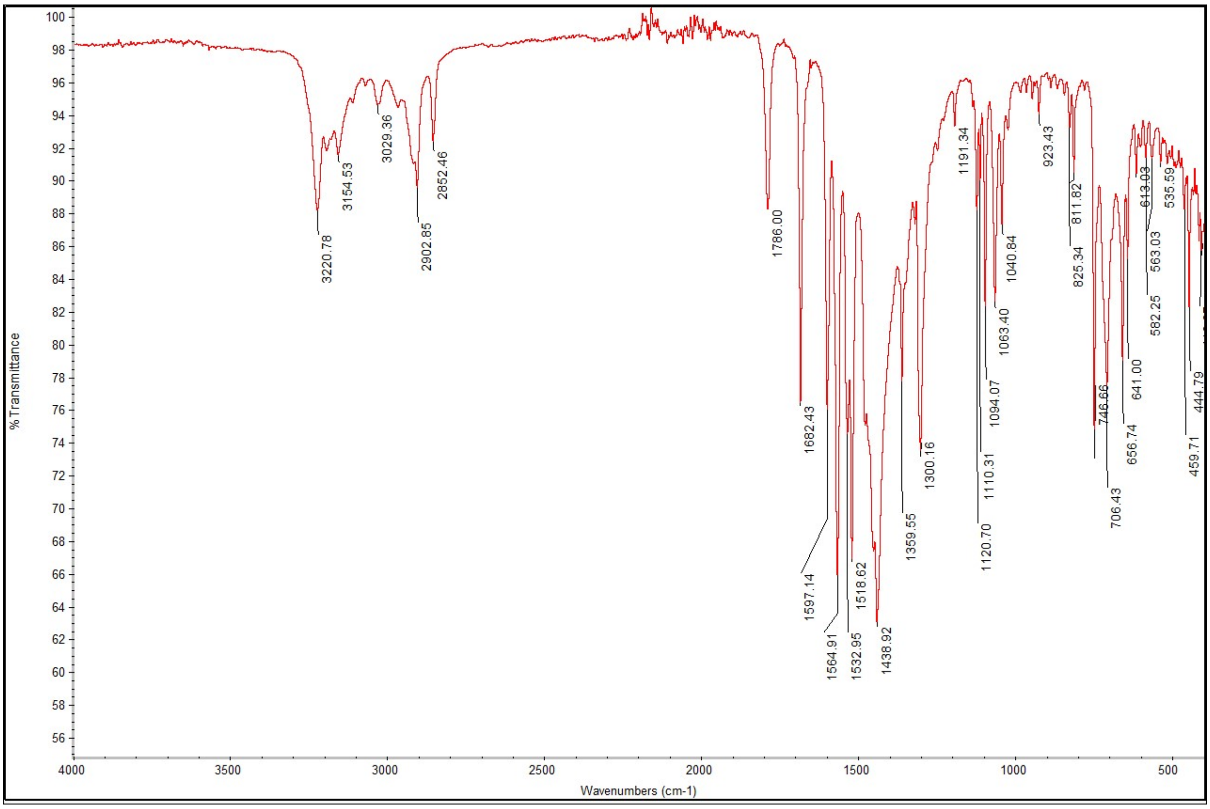

Figure S21: IR spectrum of **5**

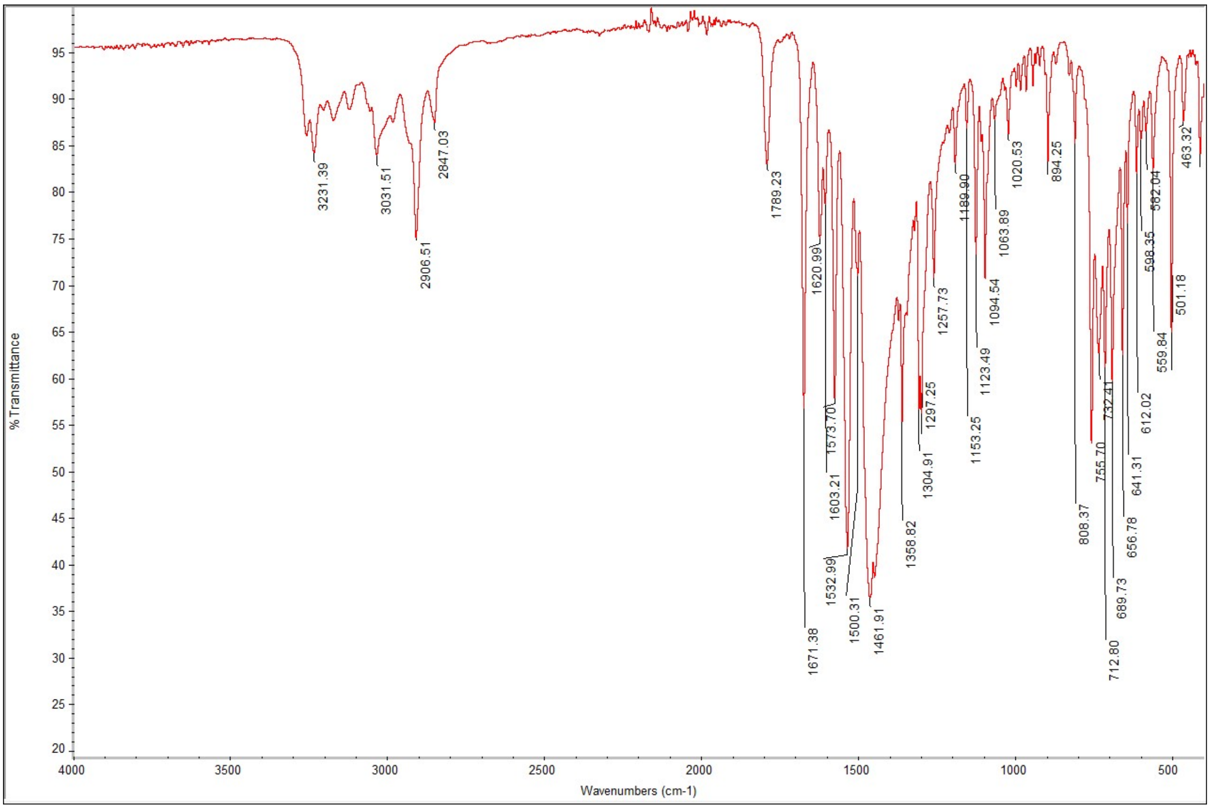

Figure S22: IR spectrum of **6**

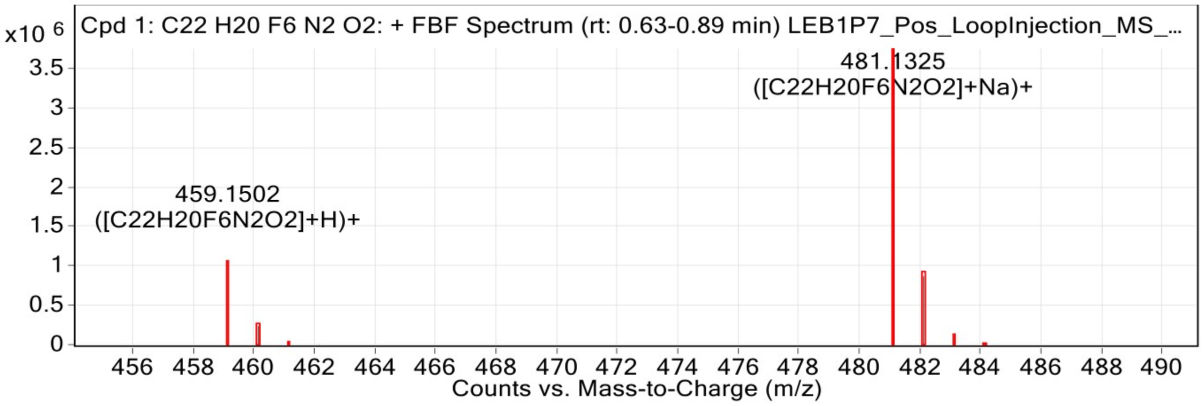

Figure S23: HRMS of **1.**

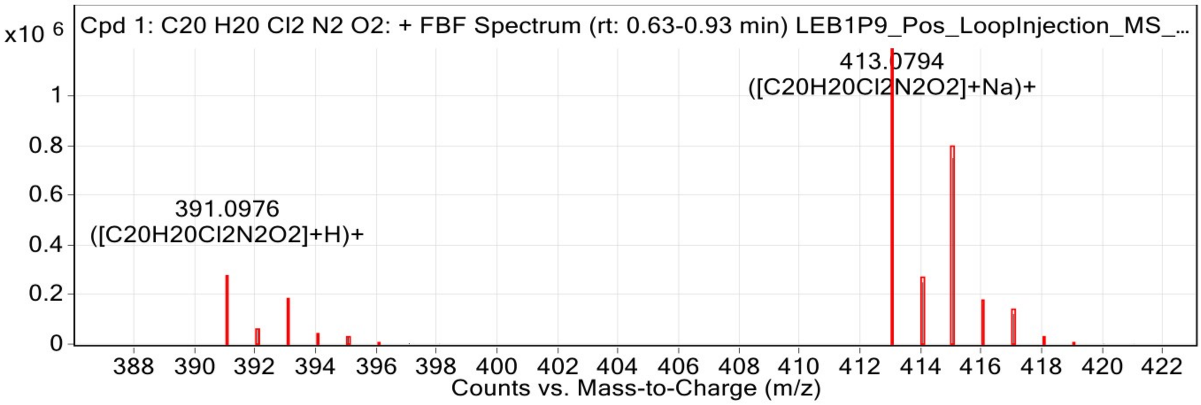

Figure S24: HRMS of **2.**

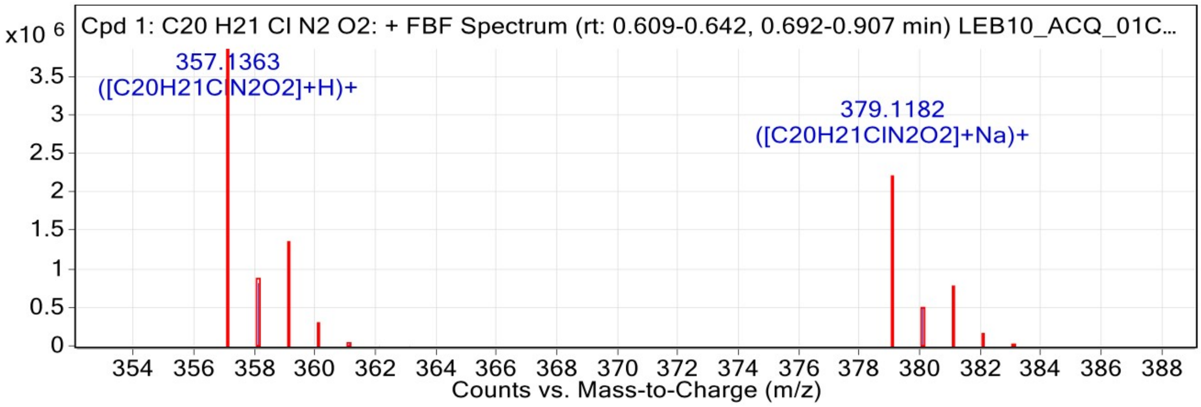

Figure S25: HRMS of **3.**

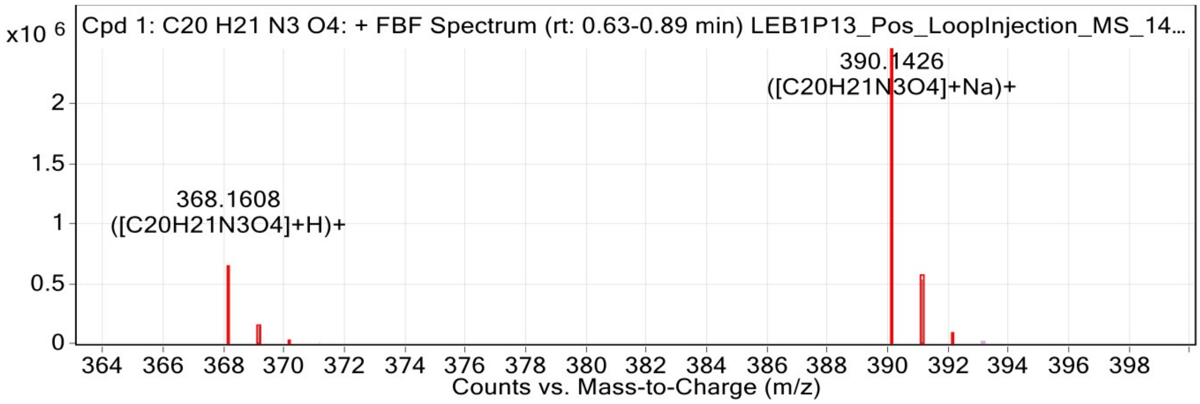

Figure S26: HRMS of **4.**

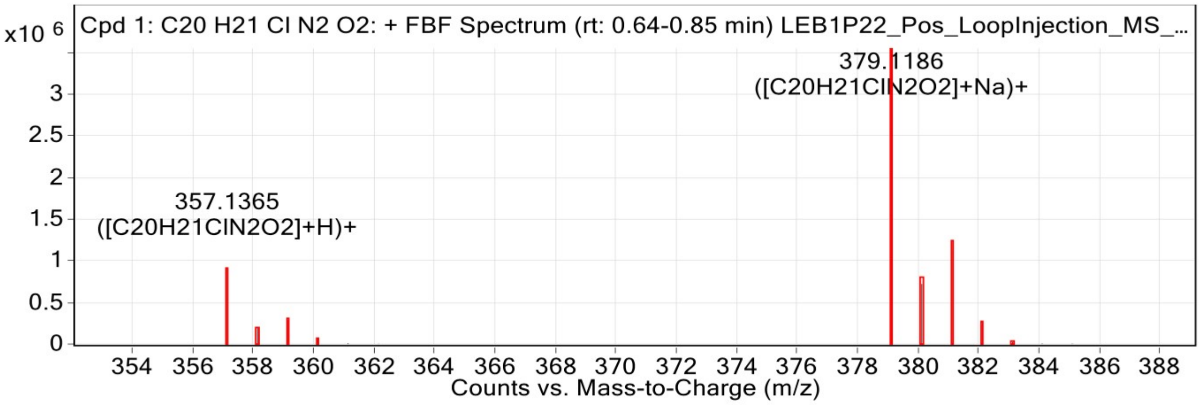

Figure S27: HRMS of **5.**

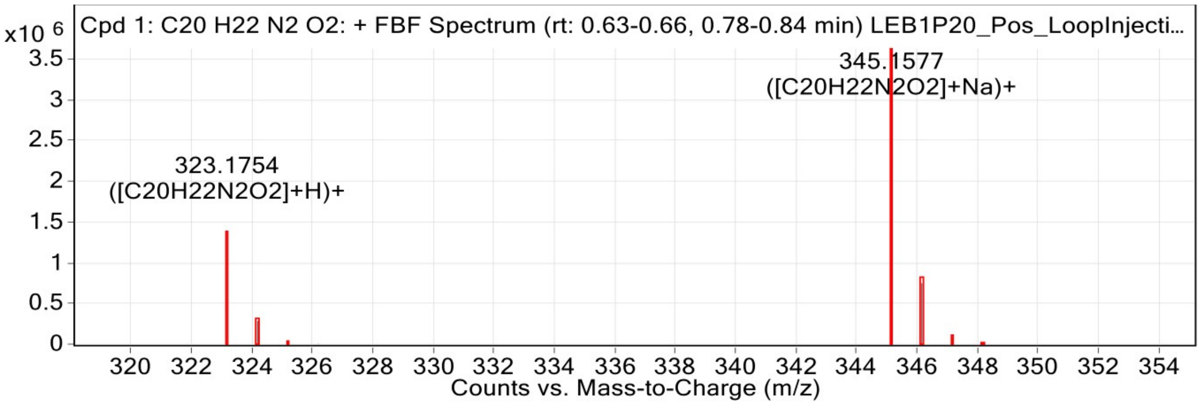

Figure S28: HRMS of **6.**

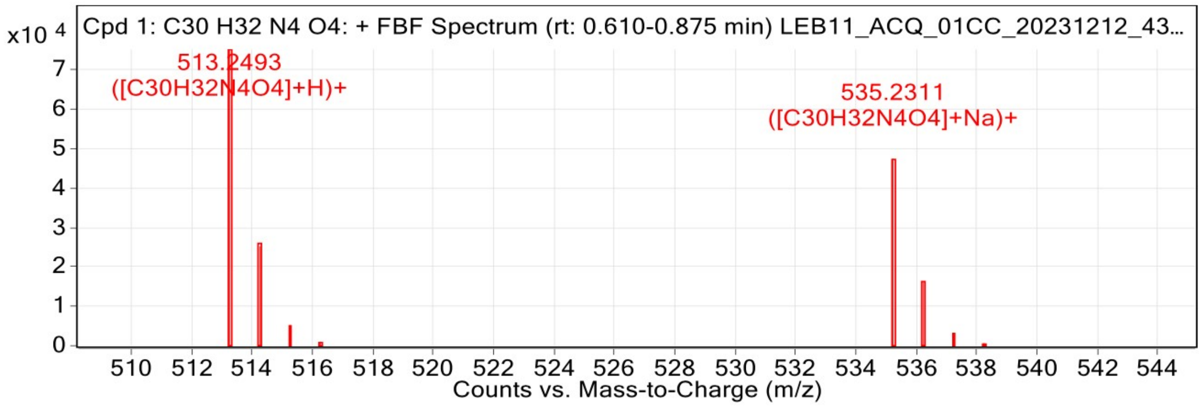

Figure S29: HRMS of **7**.

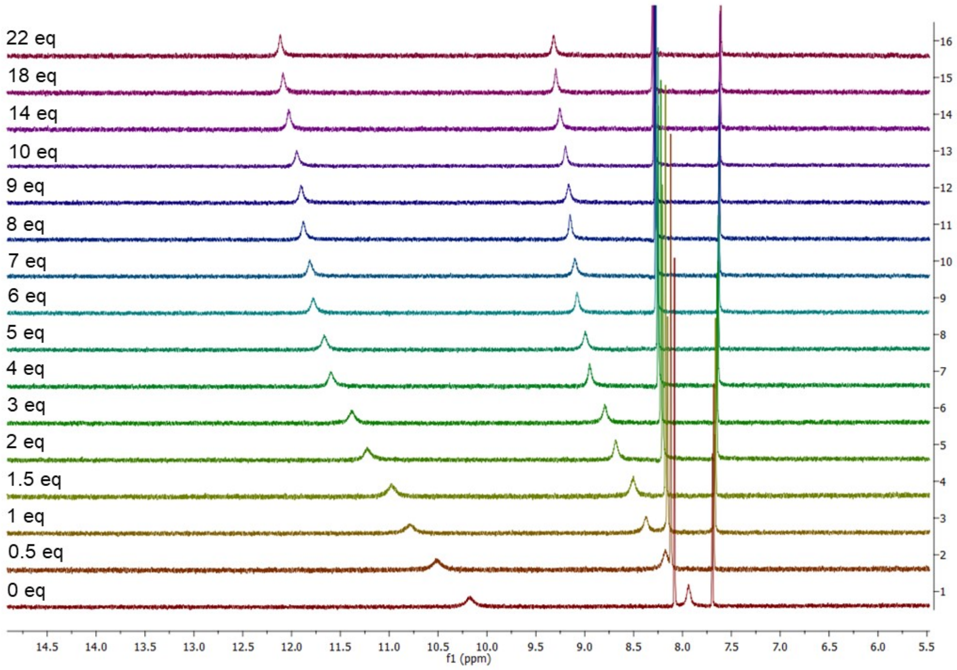

Figure S30: ^{1}H NMR stackplot, 5.5 ppm – 14.5 ppm, of receptor **1** with 0.0 – 22.0 equivalents of TBACl in DMSO-d_6/0.5 % H_2O.

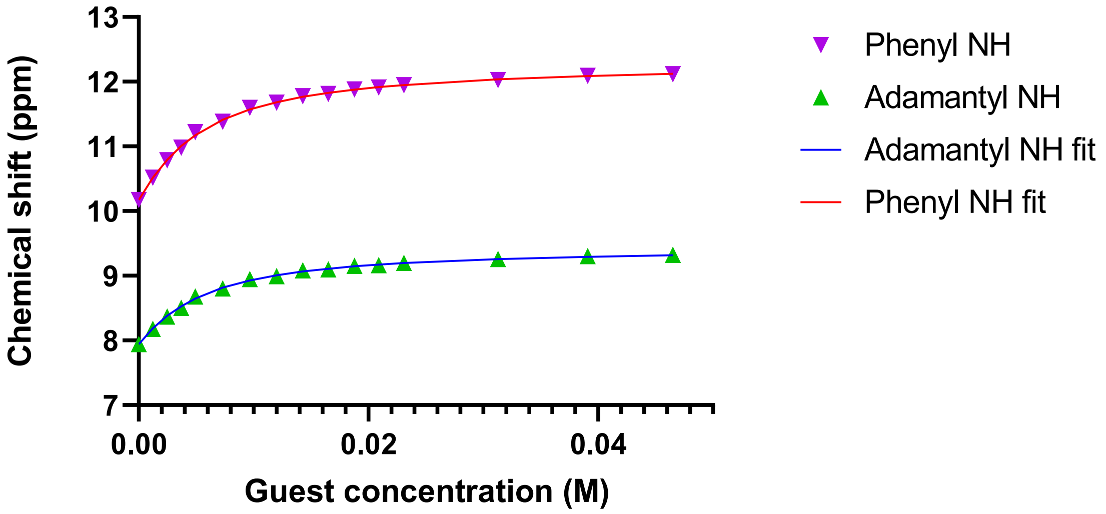

Figure S31: Fitted binding isotherm for the titration of **1** (2.5×10^{-4} M) in the presence of increasing concentrations of Cl^- in DMSO-d_6/0.5% H_2O. The data is fitted to a 1:1 binding model and shows the chemical shift of the NH signals throughout the titration. Ka = 234.7 M^{-1}, Error = $\pm$1.89 %.http://app.supramolecular.org/bindfit/view/262a813b-f466-4154-abda-596f9d67e794

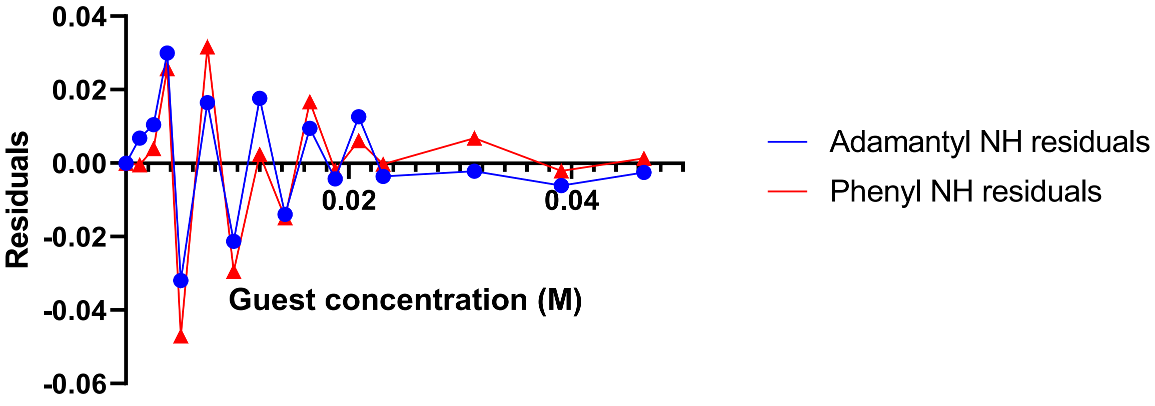

Figure S32: Residuals plot of **1**

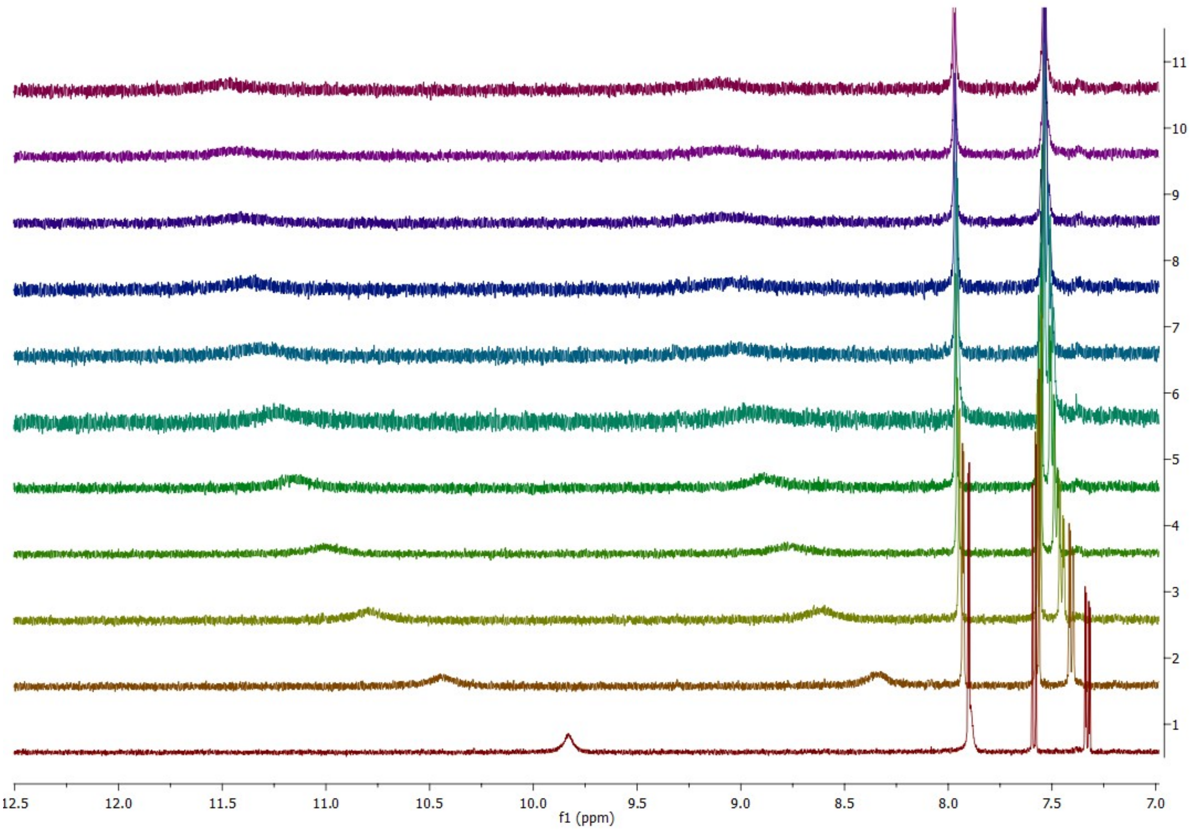

Figure S33: ^{1}H NMR stackplot, 7.0 ppm – 12.5 ppm, of receptor **2** with 0.0 – 10.0 equivalents of TBACl in DMSO-d_6/0.5 % H_2O.

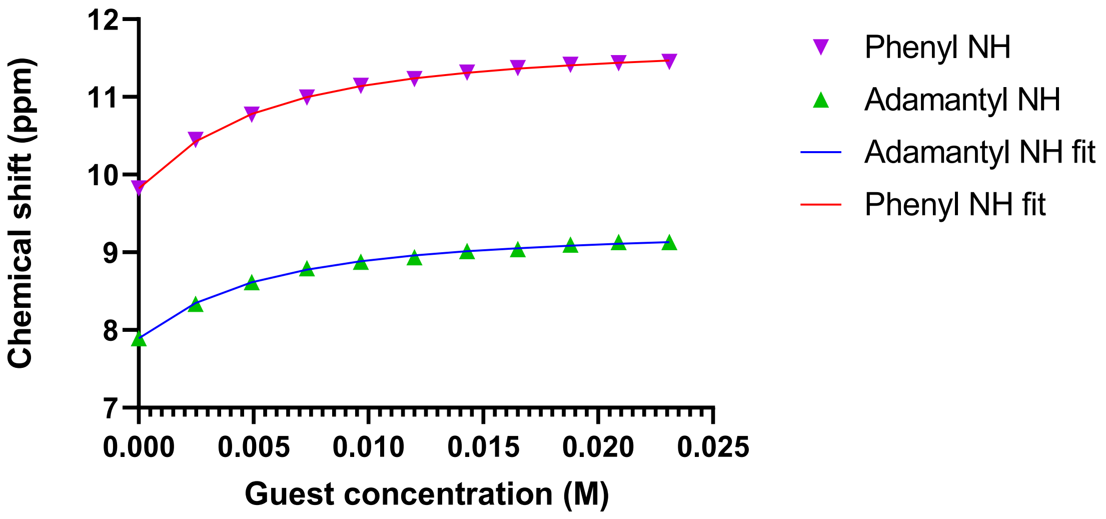

Figure S34: Fitted binding isotherm for the titration of **2** (2.5 X 10^{-4} M) in the presence of increasing concentrations of Cl^- in DMSO-d_6/0.5% H_2O. The data is fitted to a 1:1 binding

model and shows the chemical shift of the NH signals throughout the titration. Ka = 263.1 M^{-1}, Error = $\pm$2.19 %.http://app.supramolecular.org/bindfit/view/b7016002-416a-4050-96f8-64b3f8ec9571

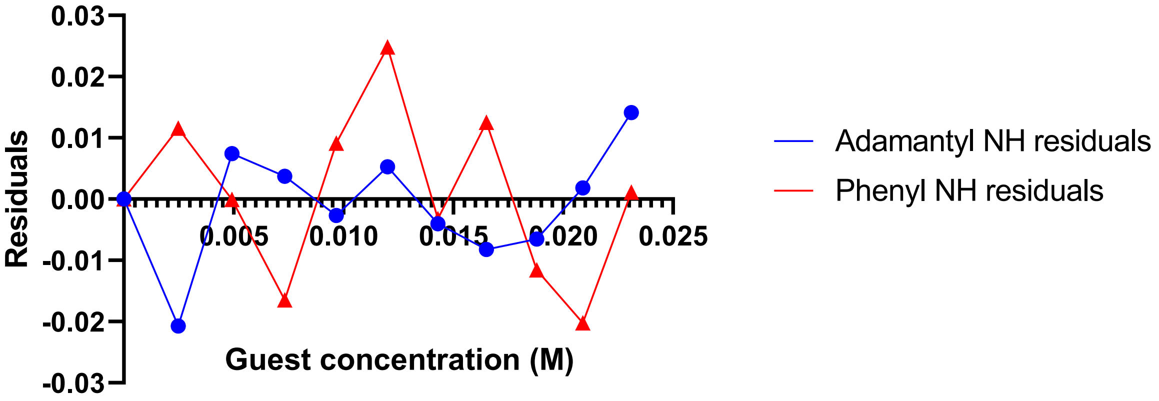

Figure S35: Residuals plot of **2.**

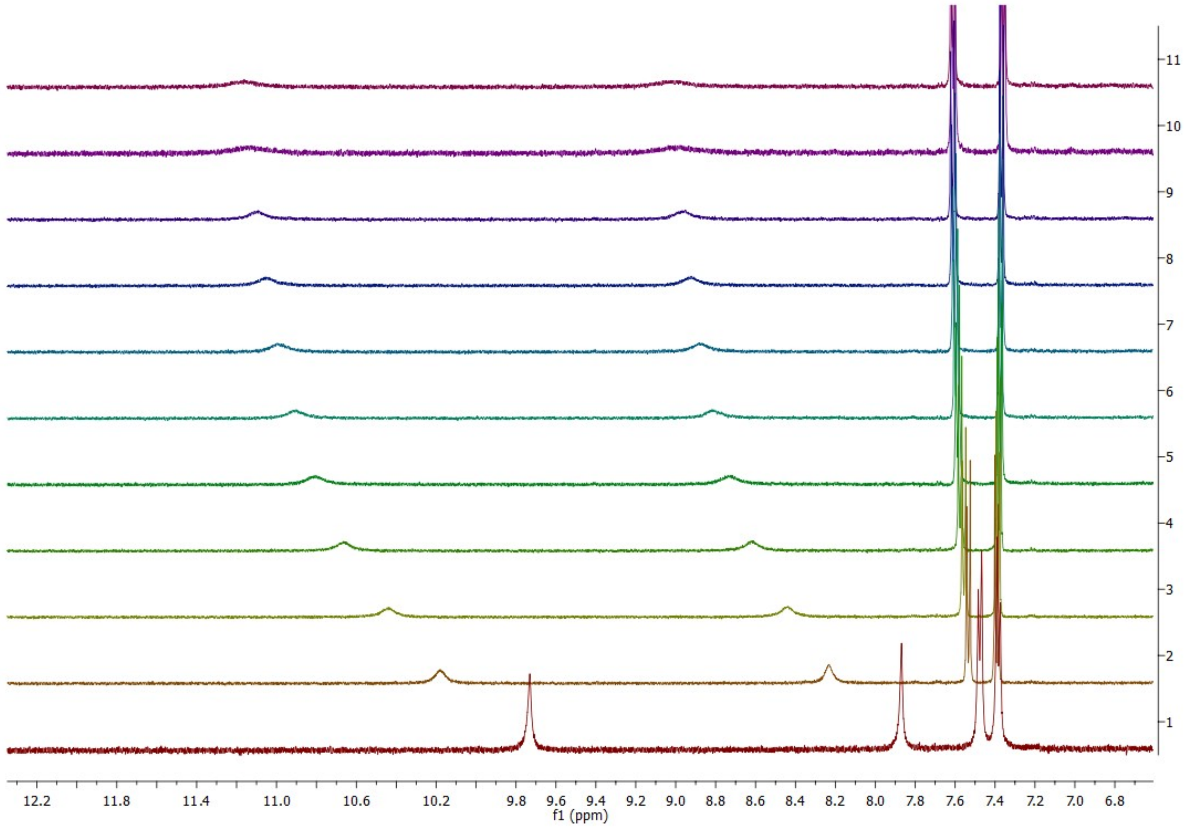

Figure S36: ^{1}H NMR stackplot, 6.5 ppm – 12.3 ppm, of receptor **3** with 0.0 – 10.0 equivalents of TBACl in DMSO-d_6/0.5 % H_2O.

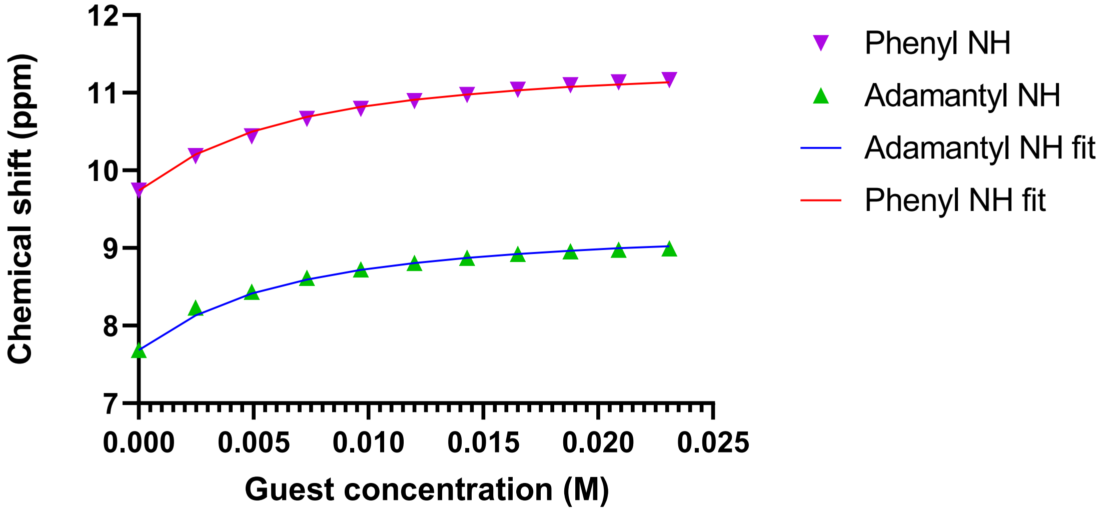

Figure S37: Fitted binding isotherm for the titration of **3** (2.5 X 10^{-4} M) in the presence of increasing concentrations of Cl^- in DMSO-d_6/0.5% H_2O. The data is fitted to a 1:1 binding model and shows the chemical shift of the NH signals throughout the titration. Ka = 206.7 M^{-1}, Error = ±4.83 %.http://app.supramolecular.org/bindfit/view/9b018c18-4c22-49d2-901f-88b13e9e2ed5

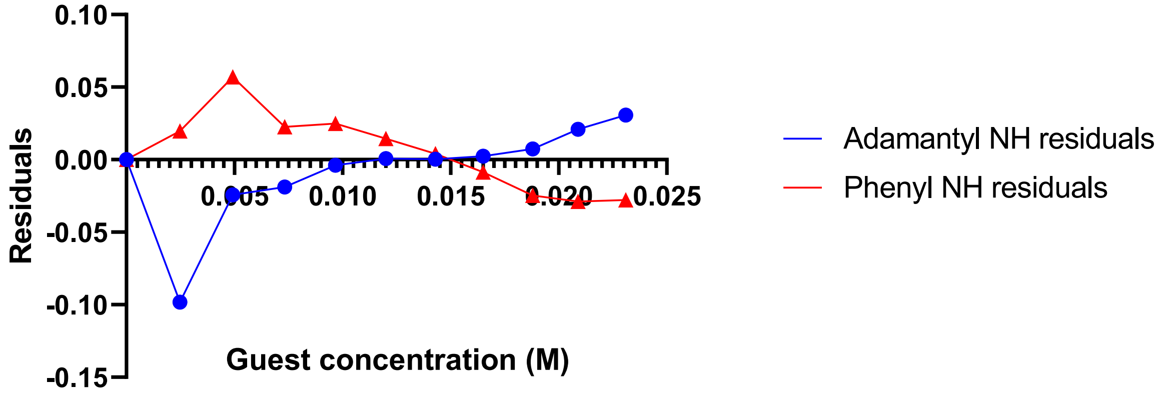

Figure S38: Residuals plot of **3**

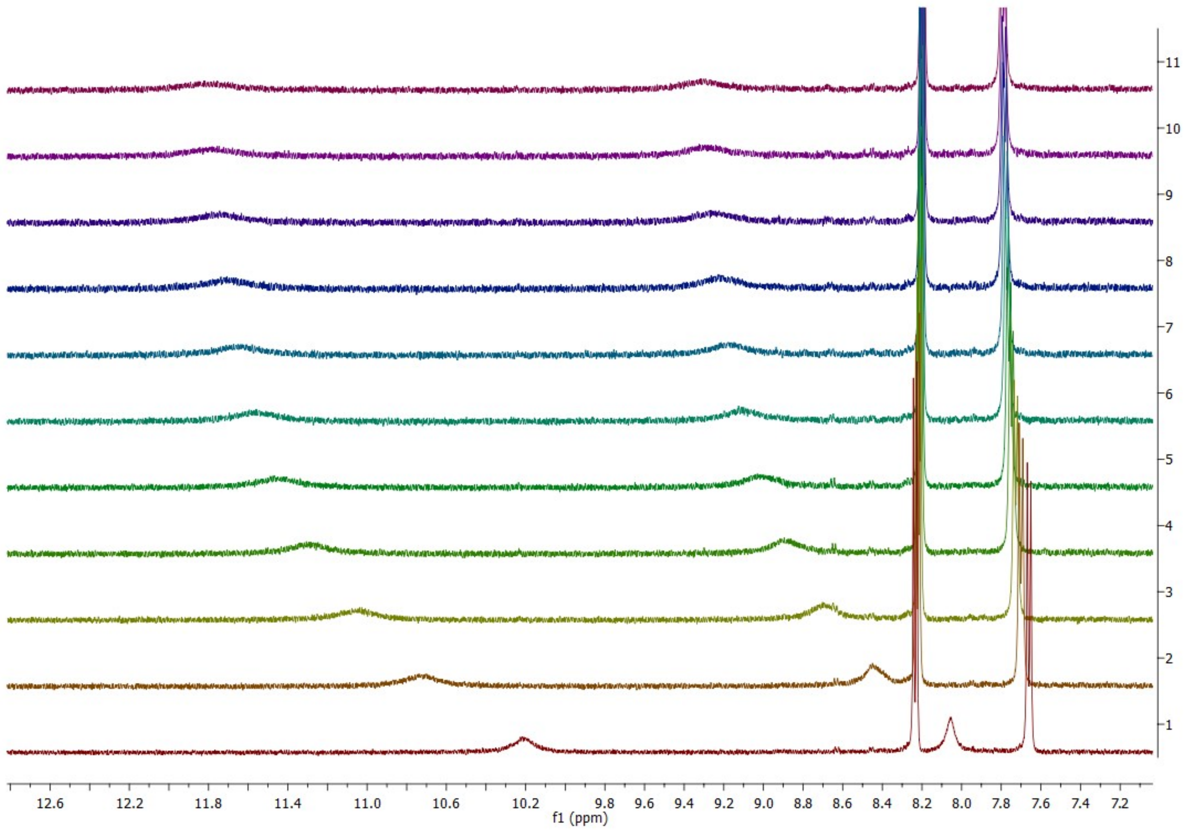

Figure S39: ^{1}H NMR stackplot, 7.0 ppm – 12.5 ppm, of receptor **4** with 0.0 – 10.0 equivalents of TBACl in DMSO-d_6/0.5 % H_2O.

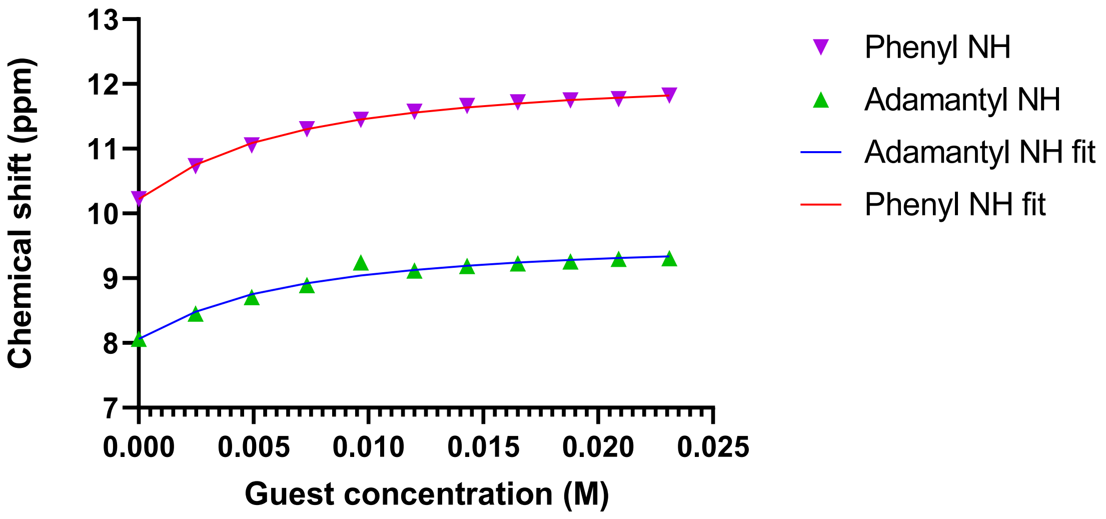

Figure S40: Fitted binding isotherm for the titration of **4** (2.5 X 10^{-4} M) in the presence of increasing concentrations of Cl^- in DMSO-d_6/0.5% H_2O. The data is fitted to a 1:1 binding

model and shows the chemical shift of the NH signals throughout the titration. Ka = 201.5 M^{-1}, Error = ±7.1 %. http://app.supramolecular.org/bindfit/view/5ded008c-1ca5-4e9e-bde1-562755489b61

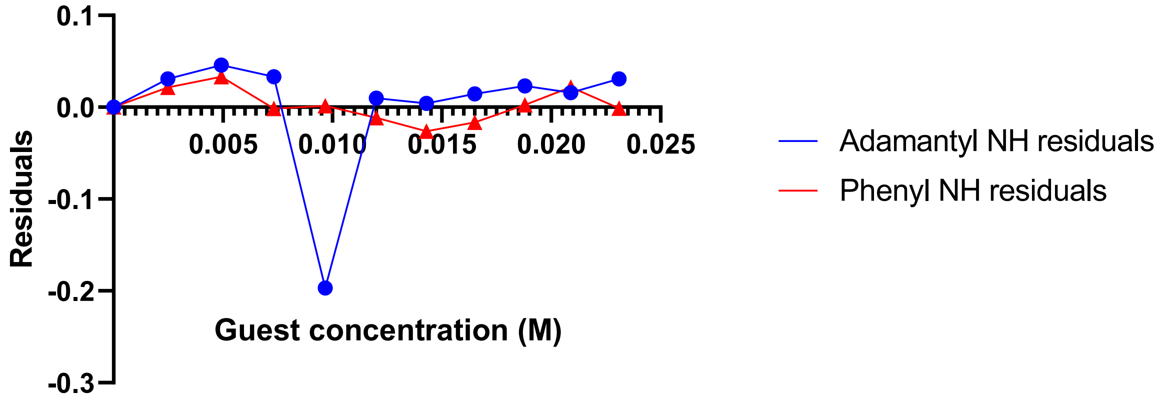

Figure S41: Residuals plot of **4.**

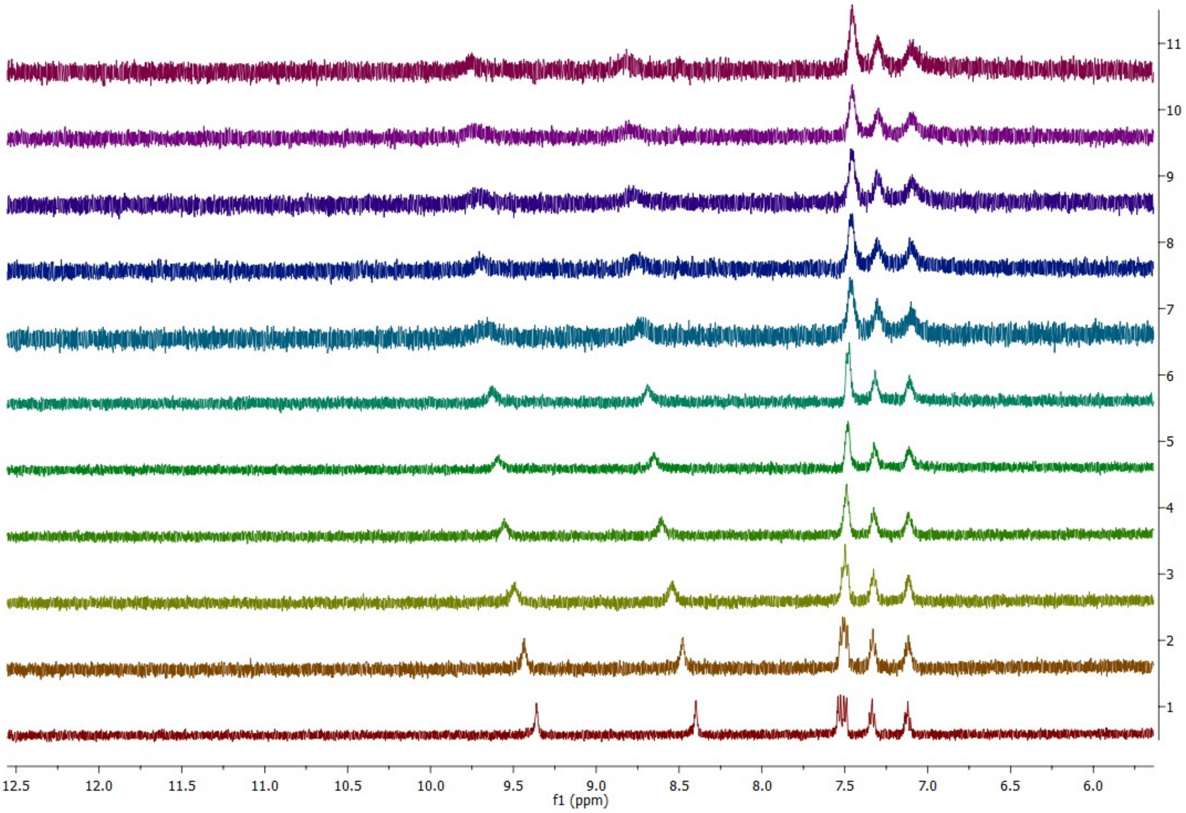

Figure S42: ^{1}H NMR stackplot, 5.5 ppm – 12.5 ppm, of receptor **5** with 0.0 – 10.0 equivalents of TBACl in DMSO-d_6/0.5 % H_2O.

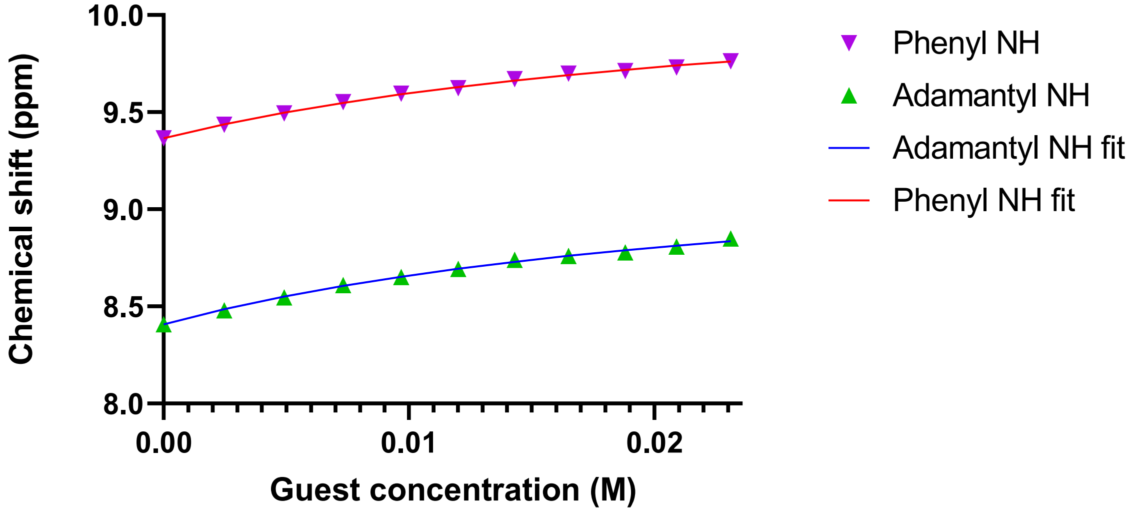

Figure S43: Fitted binding isotherm for the titration of **5** (2.5×10^{-4} M) in the presence of increasing concentrations of Cl^- in DMSO-d_6/0.5% H_2O. The data is fitted to a 1:1 binding

model and shows the chemical shift of the NH signals throughout the titration. Ka = 44.2 M^{-1}, Error = $\pm$1.91 %. http://app.supramolecular.org/bindfit/view/d9e050cf-a5e5-480b-bb26-f6f1a6ad4682

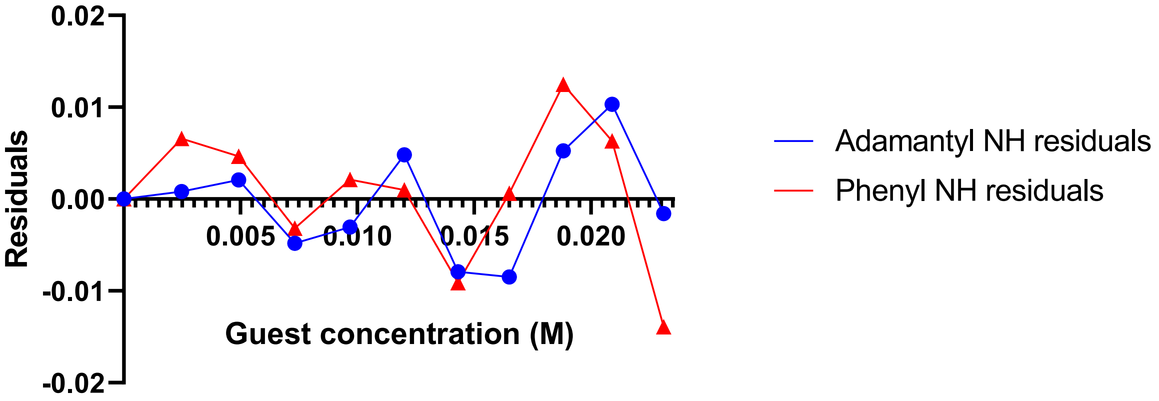

Figure S44: Residuals plot of **5.**

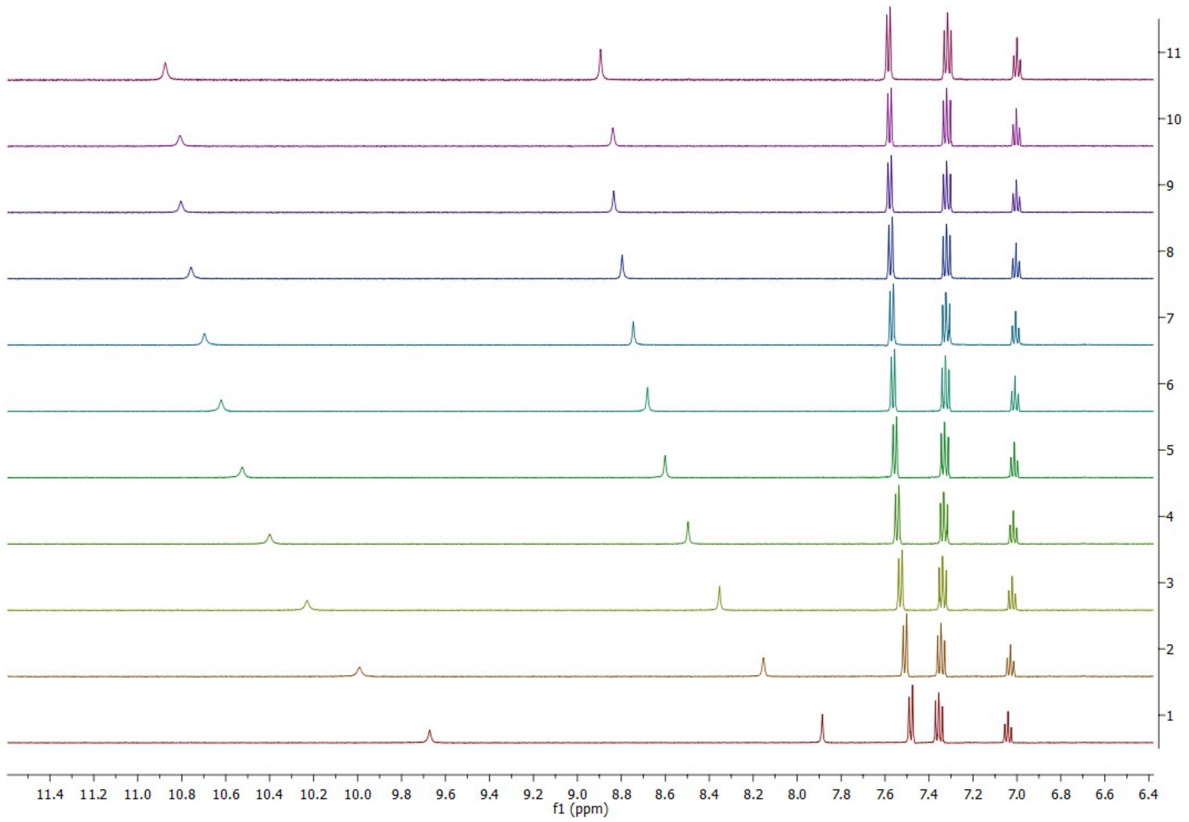

Figure S45: ^{1}H NMR stackplot, 6.4 ppm – 11.5 ppm, of receptor **6** with 0.0 – 10.0 equivalents of TBACl in DMSO-d$_6$/0.5 % H_2O.

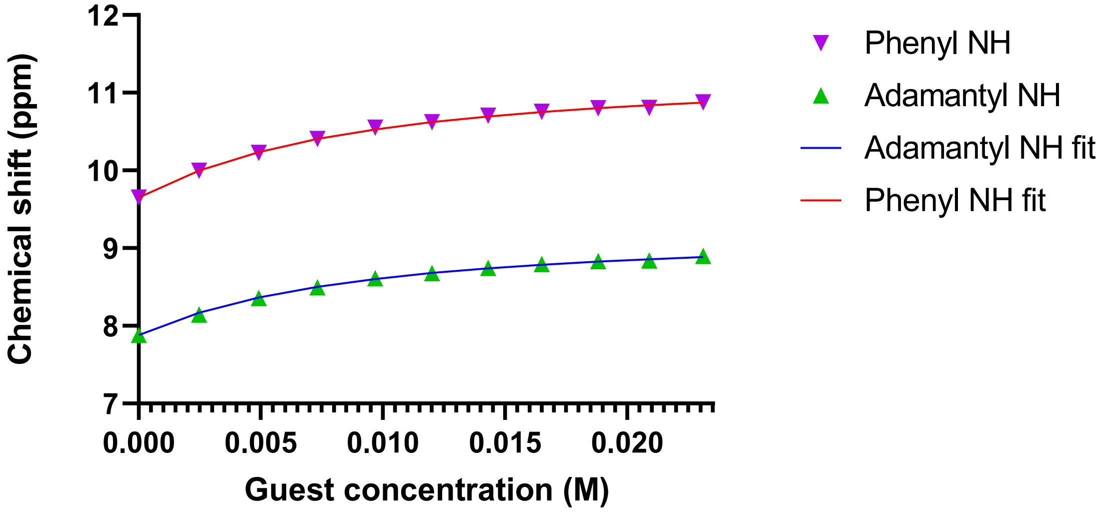

Figure S46: Fitted binding isotherm for the titration of **6** (2.5×10^{-4} M) in the presence of increasing concentrations of Cl^- in DMSO-d_6/0.5% H_2O. The data is fitted to a 1:1 binding model and shows the chemical shift of the NH signals throughout the titration. Ka = 138.3 M^{-1}, Error = $\pm$1.98 %. http://app.supramolecular.org/bindfit/view/e4711eb2-5254-4862-a90a-20d994c8018d

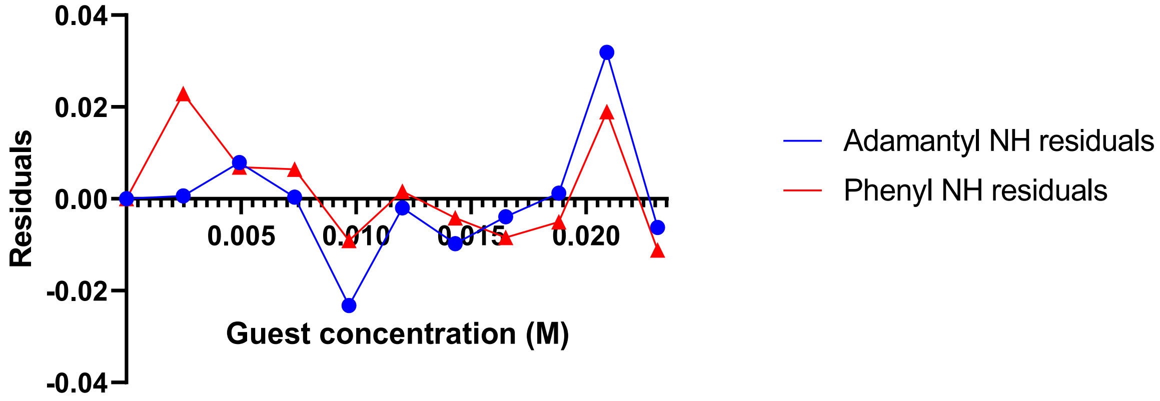

Figure S47: Residuals plot of **6.**

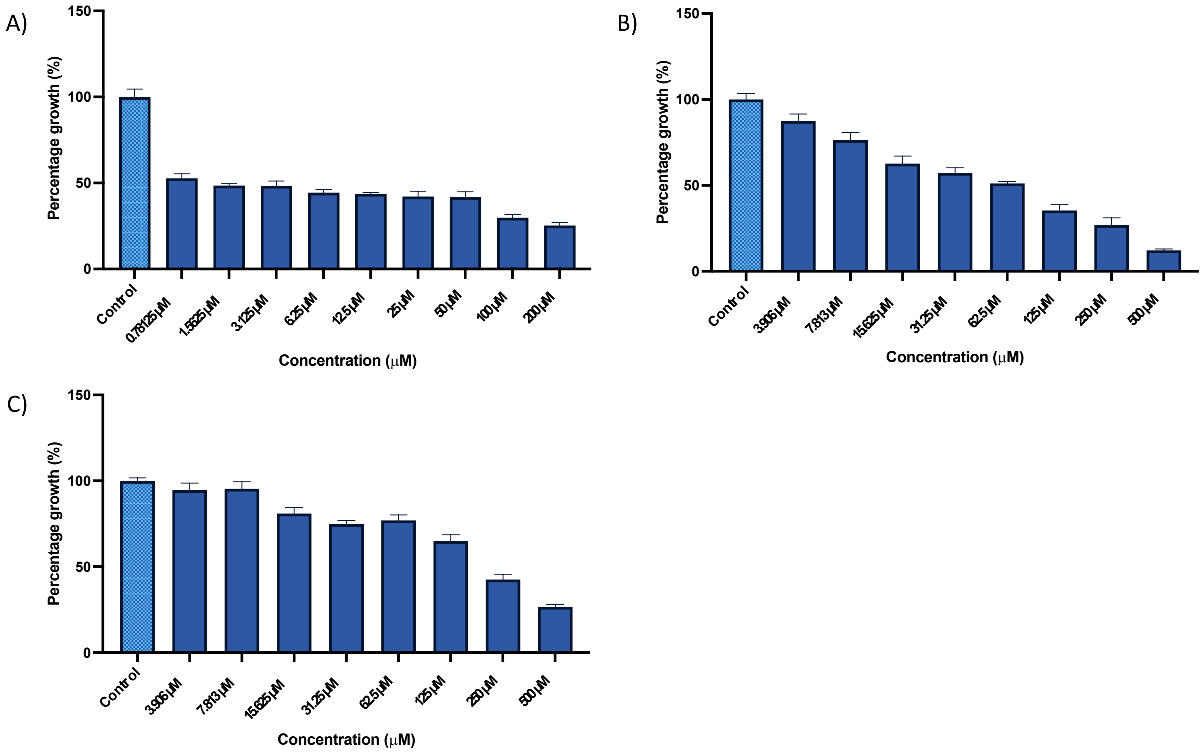

Figure S48. *S. aureus* Bacterial culture optical density represented as percentage growth (%) relative to control. All samples**; 1** (A), **2** (B**),** and **4** (C) were treated to a concentration range of either 500 – 3.9 µM or 200 µM – 781 nM. Upon treatment all samples were incubated at 37 ^{o}C for 24 h. All values represented as the mean of 8 replicates ± SEM.

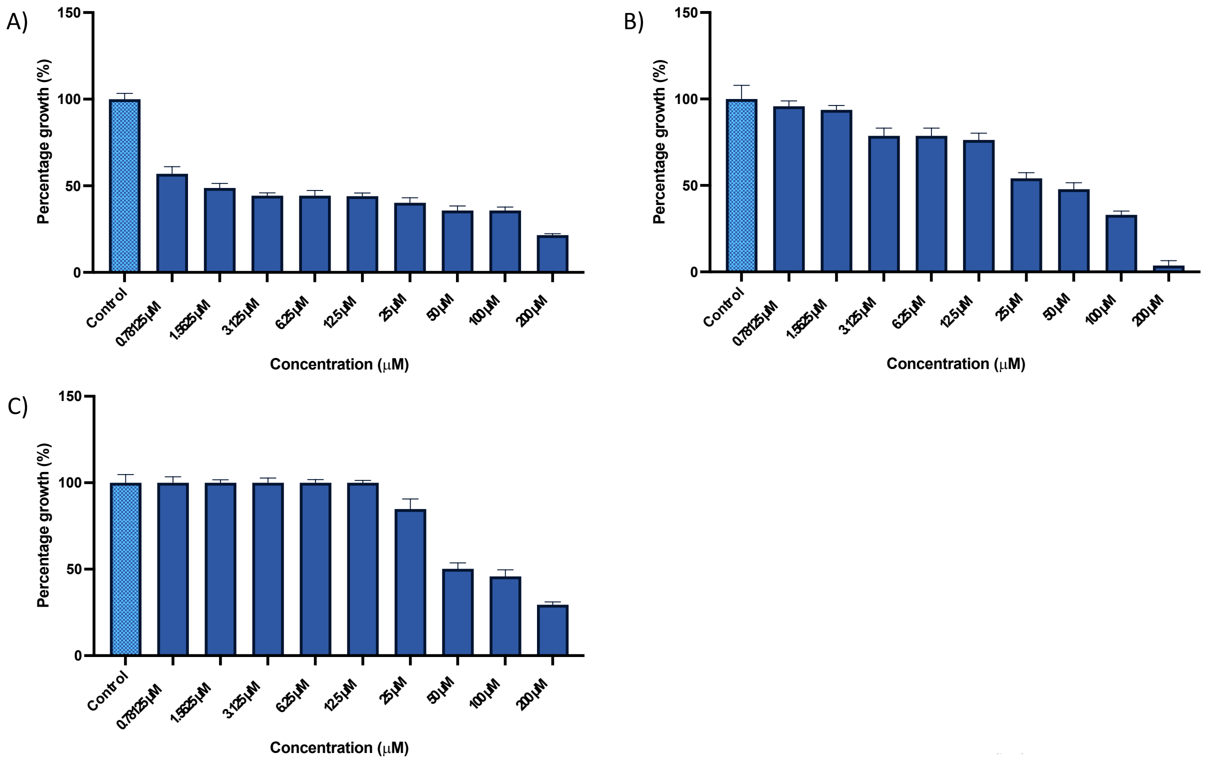

Figure S49. Methicillin-resistant *S. aureus* Bacterial culture optical density represented as percentage growth (%) relative to control. All samples**; 1** (A**), 2** (B**),** and **4** (C) were treated to a concentration range of either 500 – 3.9 µM or 200 – 781 nM. Upon treatment all samples were incubated at 37 ^{o}C for 24 h. All values represented as the mean of 8 replicates <u>+</u> SEM.

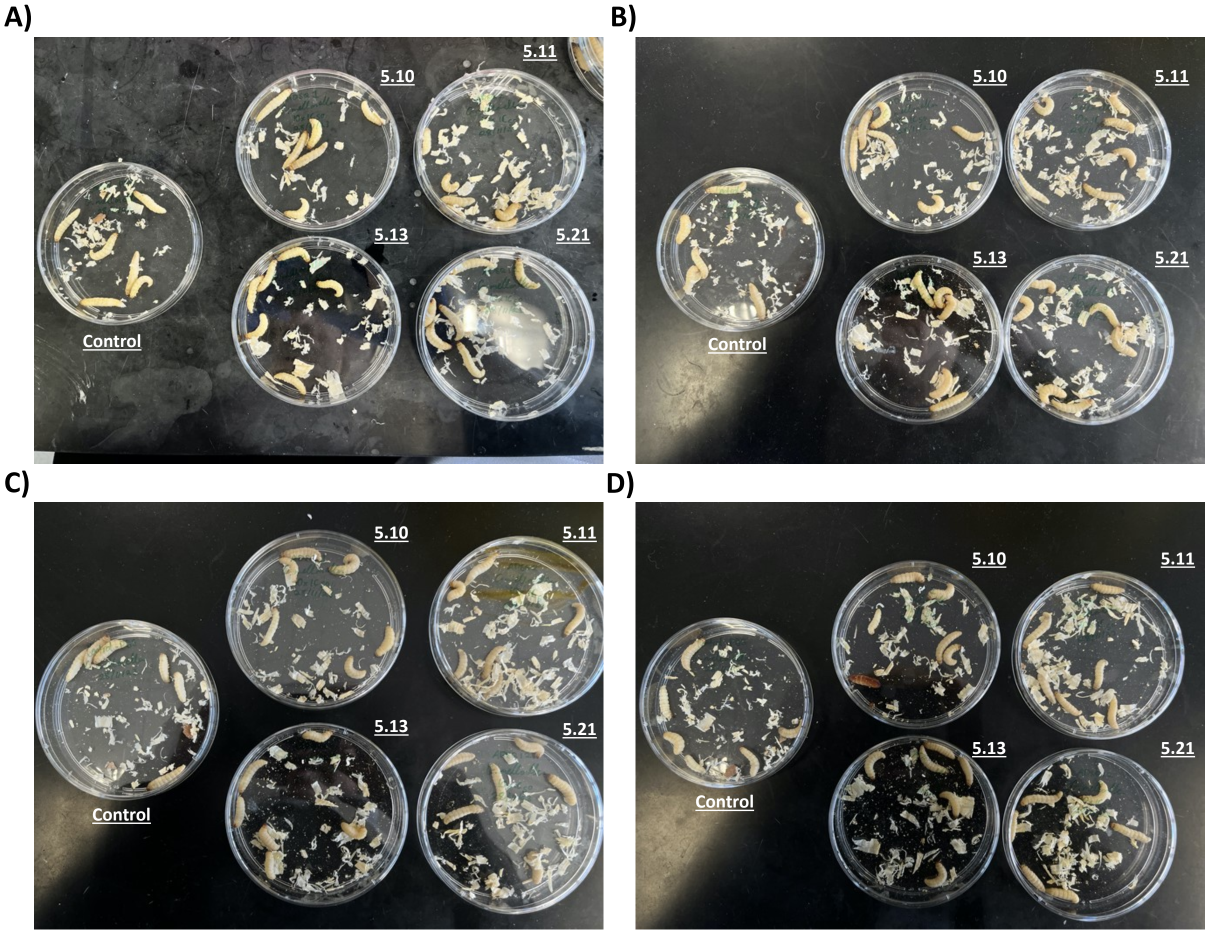

Figure S50. *In-vivo* toxicity analysis of **1**, **2**, and **4**, in the model organism *G. mellonella.* A) larval populations inoculated with 10x IC_{50} of **1**, **2**, and **4** or PBS (control) after 0 hrs; B) larval populations inoculated with 10x IC_{50} of **1**, **2**, and **4** or PBS (control) after 24 hrs; C) larval populations inoculated with 10x IC_{50} of **1**, **2**, and **4** or PBS (control) after 48 hrs; D) larval populations inoculated with 10x IC_{50} of **1**, **2**, and **4** or PBS (control) after 72 hrs. **5.10 = 1; 5.11 = 2, 5.13 = 4**.

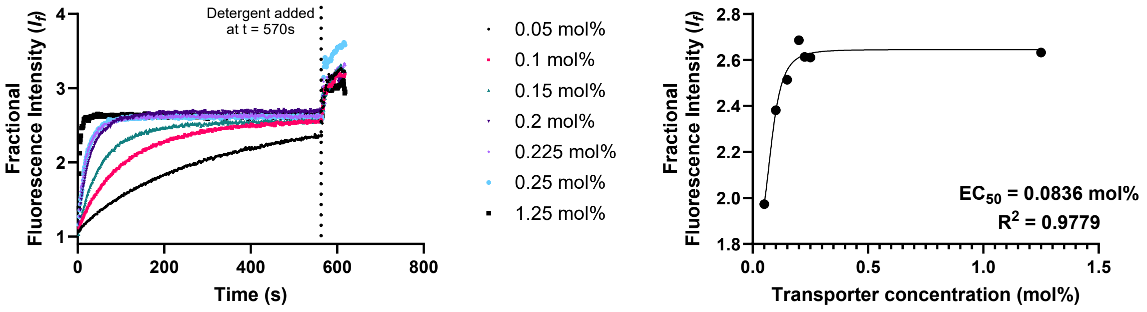

Figure S51. Results of Lucigenin Cl^-/NO_3^- anion exchange assay for Compound **1.**

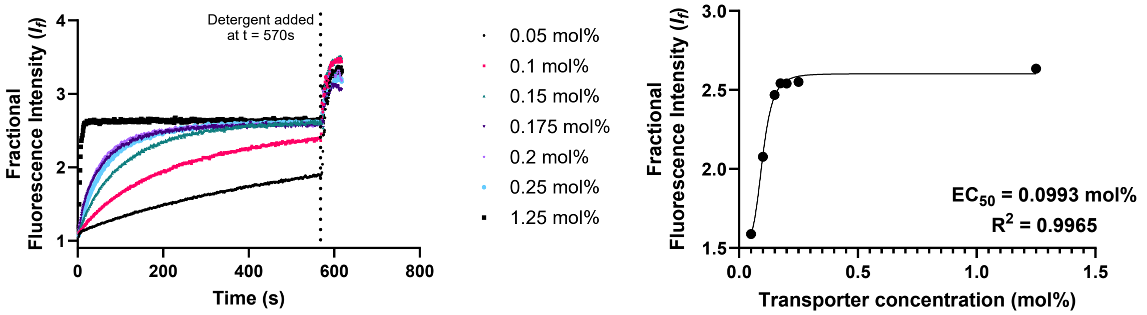

Figure S52. Results of Lucigenin Cl^-/NO_3^- anion exchange assay for Compound **2.**

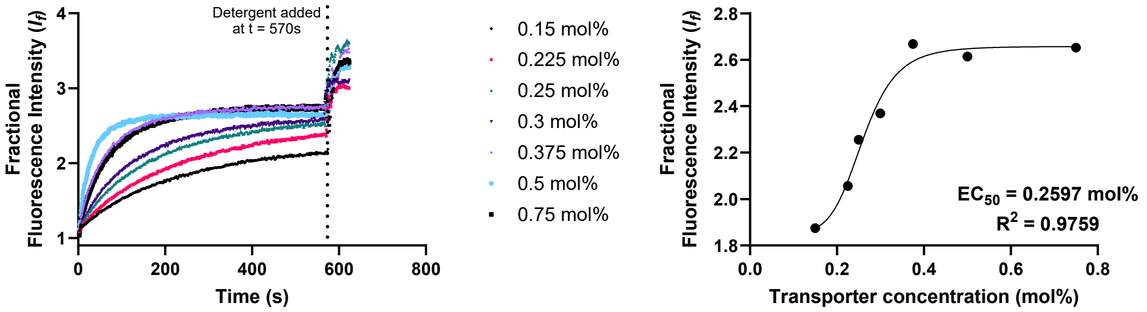

Figure S53. Results of Lucigenin Cl^-/NO_3^- anion exchange assay for Compound **3.**

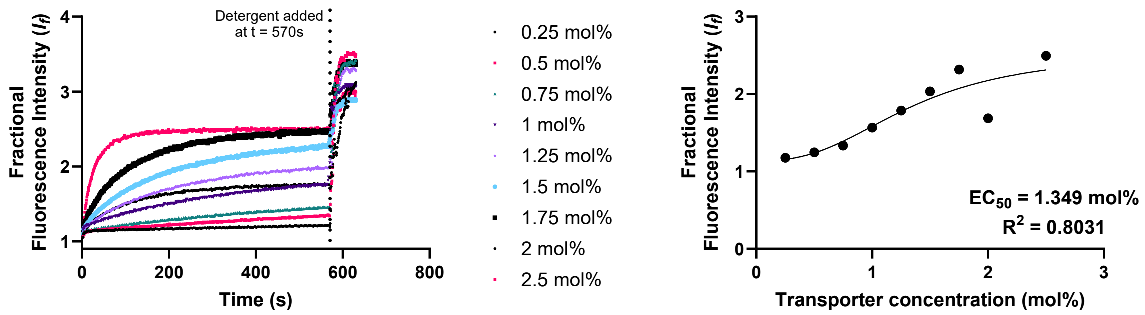

Figure S54. Results of Lucigenin Cl^-/NO_3^- anion exchange assay for Compound **5.**

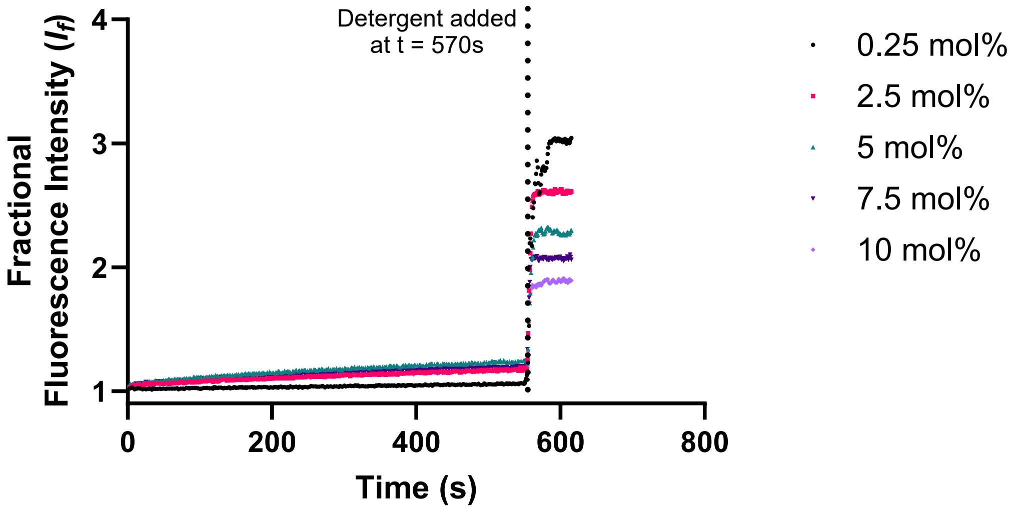

Figure S55. Results of Lucigenin Cl^-/NO_3^- anion exchange assay for Compound **6.**

References:

(1) Marchetti, L. A.; Krämer, T.; Elmes, R. B. P. Amidosquaramides – a new anion binding motif with pH sensitive anion transport properties. *Organic & Biomolecular Chemistry* **2022**, *20* (35), 7056-7066, 10.1039/D2OB01176J. DOI: 10.1039/D2OB01176J.

(2) Bujosa, S.; Castellanos, E.; Frontera, A.; Rotger, C.; Costa, A.; Soberats, B. Self-assembly of amphiphilic aryl-squaramides in water driven by dipolar π–π interactions. *Organic & Biomolecular Chemistry* **2020**, *18* (5), 888-894, 10.1039/C9OB02085C. DOI: 10.1039/C9OB02085C.

(3) Woodring, J. L.; Lu, S.-H.; Krasnova, L.; Wang, S.-C.; Chen, J.-B.; Chou, C.-C.; Huang, Y.-C.; Cheng, T.-J. R.; Wu, Y.-T.; Chen, Y.-H.; et al. Disrupting the Conserved Salt Bridge in the Trimerization of Influenza A Nucleoprotein. *Journal of Medicinal Chemistry* **2020**, *63* (1), 205-215. DOI: 10.1021/acs.jmedchem.9b01244.

(4) Zhang, T.; Hong, X.-Q.; Zhi, H.-T.; Hu, J.; Chen, W.-H. Synthesis and mechanism of biological action of morpholinyl-bearing arylsquaramides as small-molecule lysosomal pH modulators. *RSC Advances* **2022**, *12* (35), 22748-22759, 10.1039/D2RA02146C. DOI: 10.1039/D2RA02146C.
(5) Işık, M.; Unver, M. Y.; Tanyeli, C. Modularly Evolved 2-AminoDMAP/Squaramides as Highly Active Bifunctional Organocatalysts in Michael Addition. *The Journal of Organic Chemistry* **2015**, *80* (2), 828-835. DOI: 10.1021/jo5022597.
(6) Baptista, F. R.; Devereux, S. J.; Gurung, S. P.; Hall, J. P.; Sazanovich, I. V.; Towrie, M.; Cardin, C. J.; Brazier, J. A.; Kelly, J. M.; Quinn, S. J. The influence of loops on the binding of the [Ru(phen)2dppz]2+ light-switch compound to i-motif DNA structures revealed by time-resolved spectroscopy. *Chemical Communications* **2020**, *56* (67), 9703-9706, 10.1039/D0CC03702H. DOI: 10.1039/D0CC03702H.
(7) Kumawat, L. K.; Abogunrin, A. A.; Kickham, M.; Pardeshi, J.; Fenelon, O.; Schroeder, M.; Elmes, R. B. P. Squaramide—Naphthalimide Conjugates as “Turn-On” Fluorescent Sensors for Bromide Through an Aggregation-Disaggregation Approach. *Frontiers in Chemistry* **2019**, *7*, Original Research. DOI: 10.3389/fchem.2019.00354.
(8) Chvojka, M.; Singh, A.; Cataldo, A.; Torres-Huerta, A.; Konopka, M.; Šindelář, V.; Valkenier, H. The Lucigenin Assay: Measuring Anion Transport in Lipid Vesicles. *Analysis & Sensing* **2024**, *4* (2), e202300044. DOI: https://doi.org/10.1002/anse.202300044 (acccessed 2024/12/05).
